# A Scoping Review of the Relationship between Running and Mental Health

**DOI:** 10.3390/ijerph17218059

**Published:** 2020-11-01

**Authors:** Freya Oswald, Jennifer Campbell, Chloë Williamson, Justin Richards, Paul Kelly

**Affiliations:** 1 Edinburgh Medical School, The University of Edinburgh, Edinburgh EH16 4TJ, UK; s1604637@sms.ed.ac.uk; 2 Physical Activity for Health Research Centre (PAHRC), University of Edinburgh, Edinburgh EH8 8AQ, UK; chloe.williamson@ed.ac.uk (C.W.); p.kelly@ed.ac.uk (P.K.); 3 Faculty of Health, Victoria University Wellington, Wellington 6140, New Zealand; justin.richards@vuw.ac.nz

**Keywords:** exercise, mental health, psychology, physical activity, running, jogging

## Abstract

Poor mental health contributes significantly to global morbidity. The evidence regarding physical benefits of running are well-established. However, the mental health impacts of running remain unclear. An overview of the relationship between running and mental health has not been published in the last 30 years. The purpose of this study was to review the literature on the relationship between running and mental health. Our scoping review used combinations of running terms (e.g., Run* and Jog*) and mental health terms (general and condition specific). Databases used were Ovid(Medline), Ovid(Embase), ProQuest and SportDiscus. Quantitative study types reporting on the relationships between running and mental health were included. Database searches identified 16,401 studies; 273 full-texts were analysed with 116 studies included. Overall, studies suggest that running bouts of variable lengths and intensities, and running interventions can improve mood and mental health and that the type of running can lead to differential effects. However, lack of controls and diversity in participant demographics are limitations that need to be addressed. Cross-sectional evidence shows not only a range of associations with mental health but also some associations with adverse mental health (such as exercise addiction). This review identified extensive literature on the relationship between running and mental health.

## 1. Introduction

Poor mental health contributes significantly to the global health burden [1]. The strain of mental health and behavioural disorders is estimated to account for more years of lived disability than any other chronic health ailment [1,2]. The global proportion of disability-adjusted life years caused by mental ill-health has increased from 12.7% to 14% (males) and 13.6% to 14.4% (females) from 2007 to 2017 [3]. Due to the burden and increasing prevalence of mental ill-health, effective management of mental health disorders is vital [4].

There is substantial evidence to support the relationship between physical activity (PA) and various mental health outcomes across the lifespan [5,6,7]. There has been investigation of low-intensity PA on mental health; for example, Kelly et al. (2018) reported the positive relationships between walking and mental health in an earlier scoping review [8]. However, a similar synthesis for higher-intensity PA such as running has not been reported.

While the evidence base for the benefits of running on physical health is well-established, the mental health changes from running remain unclear. Addressing the gap within this knowledge is valuable as running is a form of PA popular among many population groups [9]. Inclusive organisations such as “Couch to 5k” [10], “Girls on the run” [11] and “Parkrun” can support running while promoting well-being and satisfaction with physical health, facilitating socialisation and community connectedness, and reducing loneliness [12,13,14]. In primary care settings, national initiatives such as “Parkrun-Practice” promote well-being through running [15].

In recent years, there has been a transition within healthcare to focus on disease morbidity rather than disease mortality, in particular with a drive to improve global mental health [16]. There is increasing prevalence of mental ill-health; therefore, effective management of mental health disorders is vital [4]. In order to investigate any differences in mental health effects between high and low intensities of running, all genres of running must be considered including jogging, sprinting, marathon running and orienteering.

To the best of the authors’ knowledge, no recent reviews of the relationship between running and mental health are available. The synthesis provided by this review will enable healthcare practitioners, psychologists and policy makers to better advise on running for mental health. It will also identify key gaps in the literature for future research. The aims of this scoping review are the following:

(1) to provide an overview of what is known regarding the relationship between running and mental health outcomes in all age groups and populations
(2) to highlight current knowledge gaps and research priorities

## 2. Materials and Methods

A scoping review was concluded to be the most appropriate to address the research aims as it provides an overview of the volume and distribution of the evidence base as well as highlights where more research is warranted. The review followed the five-stage scoping review framework proposed by Arksey and O’Malley and was guided by the Preferred Reporting Items for Systematic Reviews and Meta-Analyses (PRISMA) scoping review extension checklist (Appendix A) [17,18].

### 2.1. Identify Research Question

Research questions were developed to address the research aims: “What is known about the effects of running on mental health outcomes?” and “What are the current knowledge gaps?”. Research question formulation was guided by item 4 in the PRISMA scoping review extension checklist (Appendix A). The definition of running included jogging, sprinting, marathon running, orienteering and treadmill running. A wide range of intensities were included as the aim of the scoping review was to provide an overall picture of the relationship between running (of various intensities) and mental health.

### 2.2. Identify Relevant Outcomes

Mental health outcomes were informed by Kelly et al. (2018) [8], who reviewed the relationships between walking and mental health (Table 1). Measures or disorders of cognitive dysfunction were considered neurological and thus outside the scope of this review. Eating disorders were included as they significantly impair physical health or psychosocial functioning. Health-related quality-of-life was excluded as it was considered to incorporate physical, social, emotional and mental factors.

### 2.3. Identify Relevant Studies

Studies were included based on the following criteria:

- Any geographical location
- All years between 1970 and 2019
- Quantitative effects of running on predetermined mental health outcomes
  ○ Preventive effects (negative)
  ○ Health promotion effects (positive)
  ○ Intervention effects
- Any age group or sex
- Human studies
- Designs including primary research (cross-sectional, longitudinal, interventions and natural experiments with pre-post measures with or without non-running comparisons)
- Studies that mentioned walking as well as running were included because it is not possible to differentiate walkers from runners in events such as Parkrun.

Studies were excluded based on the following criteria:

- Specialist groups including elite, professional or competitive athletes.
- General physical or aerobic activity, rather than exclusively running
- Qualitative and ethnographic designs
- Systematic and scoping reviews (individual studies from identified reviews were included if relevant)
- Editorials, opinion pieces, magazine/newspaper articles, case reports and papers without primary data
- Focus on secondary mental health within clinical groups with specific physical or mental conditions that is not the condition being treated with running (e.g., effects on depression in patients with cancer)
- Evidence types including guidelines, unpublished and ongoing trials, annual reports, dissertations and conference proceedings
- Animal studies
- Unavailable in English
- Running intervention was part of a wider study where differentiating the individual effect of running was not possible (e.g., combined with weight management).
- Conference abstracts that were not published as full articles

#### Search Strategy and Databases

Databases searched were Ovid (Medline), Ovid (Embase), ProQuest and SportDiscus. Databases were searched for titles and abstracts that included at least one running term with one mental health outcome term. Appropriate truncation symbols were used to account for search term variations. Common running terms were combined. Search terms and the full search syntax can be found in Appendix B. Searches were conducted for papers published up to August 2019.

### 2.4. Study Selection

All identified records were uploaded to Covidence (https://www.covidence.org), and duplicates were automatically removed. Titles and abstracts were screened, with 20% cross-checked early in the process to assess agreement between authors. Full texts were reviewed by 2 authors.

### 2.5. Charting the Data

Data extraction was completed by the lead author (F.O.) with 5% double screened by a second author (J.R.). The data extraction form was pilot tested with the first 20 studies and informed the following standardised extraction agreed upon by all authors:

(1) Author(s), year of publication and geographical location of study
(2) Mental health conditions examined
(3) Sample size and population details
(4) Study design
(5) Measures used to quantify any change in mental health outcome(s)
(6) Running dose (if applicable) and compliance (if applicable)
(7) Whether running was beneficial and the main findings

In studies that used “Profile of Mood States” (POMS) as a measurement of mood state, total mood disturbance was used in this review if reported by the authors. If the authors only reported one/some of the POMS subdimensions, these data were extracted instead.

### 2.6. Collating, Summarising and Reporting Results

Included studies were organized into 3 categories: cross-sectional studies, acute (single, double or triple) bouts of running, and long-term running interventions. For each of these 3 categories, the results were presented in two ways: (a) a descriptive numerical analysis to highlight the prevailing domains of research regarding geographical location, mental health outcomes and research methods and (b) a narrative summary of the key findings.

## 3. Results

### 3.1. Included Studies

**Figure 1:**
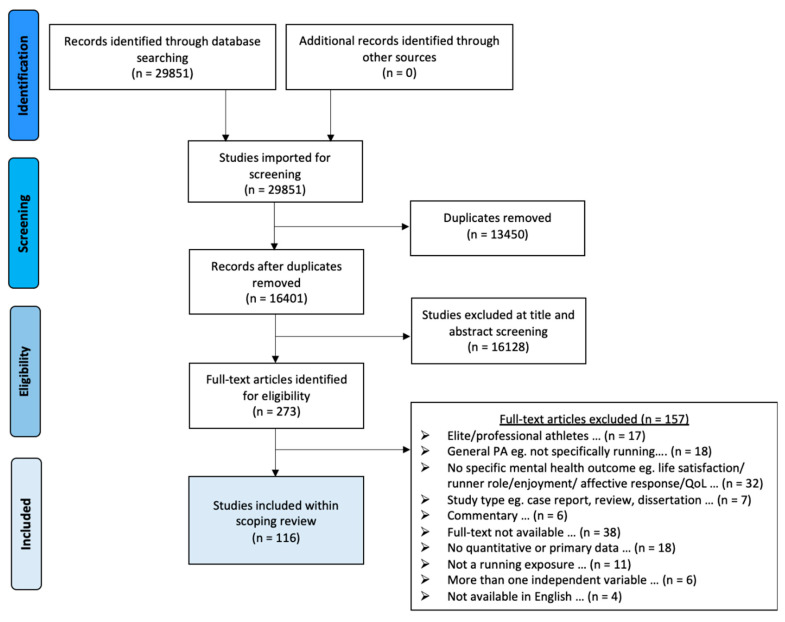
Preferred Reporting Items for Systematic Reviews and Meta-Analyses (PRISMA) depicting the identification, screening, eligibility and inclusions of texts within the scoping review.

From initial searches, 29,851 papers were identified. Following removal of duplicates, 16,401 were screened at the title and abstract levels and 273 papers were retained for full-text assessments. Ultimately, 116 papers met the inclusion criteria for this review. Figure 1 displays the PRISMA study flowchart. The results are presented in the following 3 categories: cross-sectional studies, acute bouts of running and longer-term interventions.

### 3.2. Category 1: Cross-Sectional Studies

Forty-seven studies utilised cross-sectional designs (with and without non-running comparison groups) (Table 2) [29,30,31,32,33,34,35,36,37,38,39,40,41,42,43,44,45,46,47,48,49,50,51,52,53,54,55,56,57,58,59,60,61,62,63,64,65,66,67,68,69,70,71,72,73,74,75]. These studies assessed exposure to regular running by questionnaire. Narrative description of findings of the 47 cross-sectional studies are included within Table S1 within the supplementary material.

#### 3.2.1. Runners Versus Non-Running Comparisons

Sixteen of the 47 studies directly compared measures of mental health in runners and non-running comparisons [29,33,36,37,40,41,42,45,46,47,57,61,62,63,64,73]. They found that runners had lower depression and anxiety [33,36,37,40,41,45,46,47,62], lower stress [64], higher psychological well-being [63,73], and better mood [29] compared to sedentary controls. In these studies, there was no evidence of increased prevalence of eating psychopathology in non-elite runners [42,57,61].

#### 3.2.2. Runners Only

Nineteen studies only included runners [30,31,34,35,39,44,48,49,51,55,58,65,66,67,70,74,75,76] and compared different levels and types of running. Some studies found a positive association with higher self-identity runners and low levels of depression and high self-efficacy [30,65,66,67,74]. Studies investigating marathon training found a positive relationship of marathon training with self-esteem and psychological coping [55,71]. Two questionnaires of long-distance runners found a correlation between long-distance running and disordered eating behaviours, with obligatory runners (obsessive runners who sacrificed commitments and relationships for running and suffered withdrawal symptoms if they missed a run) exhibiting traits characteristic of anorexia nervosa patients [39] and risk factors for eating disorders identified within male high school cross-country runners [70]. One study of runners training for a marathon suggested that running did not directly impact stress [72]. There were conflicting results from papers investigating negative addiction; one indicated that with more years spent running came a greater risk of negative addiction [34], while another found no relationship between years of running and addiction [58] and another found a sex difference in that commitment to running can occur without addiction in female runners but not in males [49]. Another paper found that five variables significantly predicted risk of exercise addiction in runners: weekly time spent running, childhood PA, lower educational attainment, anxiety and loneliness [75]. The remaining four cross-sectional studies of runners only found that, since participating in running, they had better emotional well-being, relief of tension, self-image and self-confidence, mood, depression, aggression and anger, anxiety and happiness, but not all reported significance or effect size [31,35,44,48,51].

A further eight studies compared groups of runners [32,38,50,53,56,60,68,69]. One paper found that females jogging with greater intensity had significantly less anxiety than those jogging at lower intensities [38]. The results from these studies showed that obligatory runners had significantly higher anxiety [53] and eating disorder measures [60,69] than non-obligatory runners and that female obligatory runners are most at risk of eating pathophysiology [60]. Non-elite marathoners showed significantly higher exercise dependence scores [56] but had more self-sufficient personalities compared to recreational runners who did not run marathons [32]. One paper did not find that exercise dependence was linked to social physique anxiety [68], while another found that runners classified as pain runners (pushed themselves until they felt pain) experienced significantly more death thoughts and death anxiety than non-pain runners [50].

#### 3.2.3. Runners Compared to Individuals with Eating Disorders

Two studies compared runners to individuals with diagnosed eating disorders but neither indicated that habitual running led to development of disordered eating or body-image problems [52,59].

#### 3.2.4. Prevented Runners

One study found that habitual runners prevented from running by illness or injury had significantly greater overall psychological distress, depression and mood disturbance than continuing runners as well as significantly lower self-esteem and body-image [43].

#### 3.2.5. Runners Compared to Gym Exercisers

A study comparing negative addiction in runners versus gym exercisers found significant association between years of participation in running and gym exercise with negative addiction, regardless of activity type [54].

#### 3.2.6. Summary of Cross-Sectional Evidence

Consistent evidence was found for a positive association between positive mental health outcomes and habitual or long-term recreational running compared to non-runners. In contrast, there was evidence that high or extreme levels of running (high frequency and long distance including marathon running) were associated with markers of running ill-health compared to levels of moderate running.

### 3.3. Category 2: Acute Bouts of Running

Narrative description of findings of the 35 studies with an acute bout of running are included within Tables S2–S4 within the supplementary material.

#### 3.3.1. Single Bouts

Twenty-three studies incorporated a design using a single bout of running to compare pre-post measurements of mood and short-term measures of mental health (Table 3*)* [77,78,79,80,81,82,83,84,85,86,87,88,89,90,91,92,93,94,95,96,97,98,99]. Twenty-two of these found positive improvement in measures of mental health (including anxiety, depression and mood); however, one found a decrease in self-efficacy of children following participation in gymnasium PACER (progressive aerobic cardiovascular endurance run) running challenge [95].

Eleven studies used a single bout of treadmill running, and all found positive pre-post differences in mental health outcomes [84,85,86,88,89,90,91,92,93,97,99]. Results found significant reductions in state-trait anxiety; total mood disturbance; and POMS subscales of anxiety, depression and confusion. A single bout of treadmill running also significantly improved self-esteem; psychological well-being; children and adolescent self-efficacy; state anxiety, depression and totally mood disturbance; adult self-efficacy; and general affective response. One study found that mood improvements were not evident until 40 min of running [88], while another found that depressed individuals participating in a treadmill run with increasing gradient improved depressed mood immediately post-run but that depressed mood increased at 30-min postexercise [93].

Three studies used a single bout of track running and found significant decreases in anxiety [78,87] and total mood disturbance [81]. Two studies found that a single outdoor run significantly improved depression scores and that even a 10-min jog caused significant mood enhancement [80,94]. Two studies found that a single bout of self-paced running significantly reduced all but one of the POMS subscales and had significant positive changes in all measures of states of affect [82,96].

There were significant improvements for self-esteem, stress and total mood disturbance following a 5-km Parkrun [98], while a 3-mile “fun-run” increased positive mood and decreased negative mood [83]. Two studies used longer runs as exposures: one found that a 1-h run significantly reduced anxiety and nonsignificantly reduced depression [79], while the other found that a 12.5-mile jog significantly improved pleasantness; decreased trait anxiety; nonsignificantly increased activation; and reduced state-anxiety, sadness, anxiety, depression and relaxation subscales [77].

#### 3.3.2. Double Bouts

There were nine studies that had a double-bout design [100,101,102,103,104,105,106,107,108] (Table 4). Eight of the nine studies were primarily designed to compare conditions rather than to compare the impact of running on mental health, including green/park versus urban, solo versus group, different pacing and different durations of running [101,102,103,104,105,106,107,108]. Seven of the eight studies found that markers of mental health improved significantly after running [101,102,103,104,105,106,107]. Only one study was designed to primarily assess the impact of running on mental health, and although there was no control, they found higher mood and feelings of pleasantness post-run but these “did not reach significance” [100].

Four studies compared park/rural versus urban running, and all found measures of mental health including anxiety, depression, mood and self-esteem improved post-run [103,104,105,107]. No paper reported a statistically significant difference in emotional benefit between park and urban conditions. Two studies compared solo versus group running: one found that anxiety reduced following both group and solo running [101], while the other found that children’s anxiety levels increased nonsignificantly following individual and group running [108]. One study compared 10- and 15-min runs and found that they produced similar psychological benefits to mood [102]. Another compared a self-paced versus prescribed-paced run and found higher self-efficacy before the prescribed-paced run compared to the self-paced run [106].

#### 3.3.3. Triple Bouts

Three studies used three bouts of running (Table 5) [109,110,111]. One study found that, while two indoor runs had a positive effect on mood, the outdoor run had an even greater benefit to mood with subjects feeling less anxious, depressed, hostile and fatigued and feeling more invigorated [109]. Another study also used 3 runs of varying intensities and found significant overall mood benefits postexercise but no significant differences between intensities [110]. One study compared 3 intensities of treadmill exercise to a sedentary control condition and found that state anxiety improved following running at 5% below and at the lactate threshold but that anxiety increased after running at 5% above the lactate threshold [111]. Overall, these studies suggest that running improves mood, that outdoor running has a greater benefit to mood and that most intensities of running improve mood, with the exception of an intensity markedly above the lactate threshold. However, only one study included a control condition [111].

#### 3.3.4. Summary of Acute Bouts

Overall, these studies suggest that acute bouts of running can improve mental health and that the type of running can lead to differential effects. The evidence suggests that acute bouts of treadmill, track, outdoor and social running (2.5–20 km and 10–60 min) all result in improved mental health outcomes. There were few differences between high and low intensities. Studies consistently show that any running improves acute/short-term mood markers, but the lack of inactive comparison conditions is a limitation to the strength of the evidence. Little variation in the demographics of participants and small sample sizes limit generalizability and precision of findings.

### 3.4. Category 3: Longer-Term Interventions

Thirty-four studies investigated the effects of more than three bouts of running on measures of mental health ranging from 2-week interventions to 1-year marathon training programmes (Table 6) [112,113,114,115,116,117,118,119,120,121,122,123,124,125,126,127,128,129,130,131,132,133,134,135,136,137,138,139,140,141,142,143,144]. Narrative description of 34 studies are available in Table S5 within the supplementary material.

Eight studies used 2–8 week running interventions [121,122,125,127,128,132,137,139]. Male regular runners deprived of running for 2 weeks had increased anxiety and depression symptoms compared to continuing runners [125]. Two 3-week interventions both found that mood improved while amateur runners had lesser anxiety on running days compared to non-running days; perceived stress in adolescents did not significantly change [132,137]. A 4-week intervention of regular treadmill running at set paces in moderately trained male runners found that an increase in intensity of runs was associated with significant increase in total mood disturbance while running at a pace with more economical values was associated with more positive mental health profiles [127]. A 7-week non-controlled intervention of weekly 40-min fixed distance outdoor rural runs increased mood in both male and female regular exercising university students, with faster runners scoring higher than slower runners [128]. An 8-week intervention of a combination of weekly group and solo jogging in middle-aged chronically stressed, sedentary women found lower anxiety and greater self-efficacy than baseline and compared to relaxation group controls [121]. Two studies used a 8-week intervention of walking/running with non-treatment controls and found significant improvements in mood and decrease of depression, including in outpatients diagnosed with mild to severe depression [122,139].

Eleven studies used 10–20 week running interventions [114,115,116,119,123,126,129,130,131,140,143]. Three 10-week walking/jogging interventions found reductions in anxiety measures, improvement of well-being and conflicting results for changes in depression measures compared to controls [115,119,129]. Another 10-week running intervention found that depression, trait anxiety and state anxiety all decreased significantly while mood improved significantly [114]. A further 10-week running intervention found that, although the exercise group was more likely to use exercise to cope with stress, there were no significant differences in stress or coping measurements between the running and comparison group [123]. Three 12-week interventions found significantly reduced stress and improvements in mood in college students compared to controls, with more mood improvement in males and in females with higher masculinity [126,130,143]. One 12-week intervention of self-directed running in recreational runners found that well-being was significantly higher during weeks when individuals ran further and ran more often while self-efficacy was related to distance ran but not to frequency of running [143]. Running interventions of 14–20 weeks improved mood and self-esteem and lowered emotional stress reactivity in college/university students compared with controls [116,131,140].

A number of studies looked at specific populations. One investigated the impact of 10 organised runs on homeless people and found significant positive correlation with perceived self-sufficiency [138]. Two investigated the effects in children and found that running significantly improved creativity and higher self-esteem subscales [117,141]. Three looked at marathon training programmes: one found a positive correlation between the trend in running and self-efficacy but was not significant [136], while another found a significant increase in self-efficacy over the programme [76]. The remaining study used participants who were already self-enrolled in a marathon, and researchers found that, while anxiety decreased initially during training, anxiety increased as marathon day approached [135].

Nine studies used subjects with known psychiatric disorders and found that longer-term interventions generally improved markers of mental health in psychiatric populations, particularly markers of depression [112,113,118,120,124,133,134,142,144]. Running interventions from 2 to 12 weeks all resulted in significant positive effects on mental health [112,118,120,124,133,142,144]. While an anti-depressive effect of exercise was apparent in patients with minor to moderate psychiatric problems, one study found that this was not reflected in patients with major depressive disorder due to issues with compliance and motivation towards the intervention [144].

#### Summary of Longer-Term Interventions

Overall, running interventions of 2–20 weeks generally show improved markers of a range of mental health outcomes compared to non-running controls, including mental health outcomes in psychiatric and homeless populations. The risk of longer-term running interventions on adverse mental health outcomes remains unclear.

### 3.5. Summary of Key Findings

The key findings of the each of the three categories of studies are summarised in Table 7. 

### 3.6. Evidence Gaps

As well as reporting the available evidence, this review also aimed to identify key gaps in the evidence base for running and mental health. Consideration of sample demographics in the *n* = 116 included studies resulted in the following gaps being identified:

- lack of studies in those aged under 18 (Only four acute bout studies [89,95,107,108] and two longer term interventions [117,141] looked directly at children under age 15, while a further 2 studies looked specifically at adolescents [70,137]);
- lack of studies in those aged over 45;
- lack of gender-specific approaches;
- few studies investigating clinical populations; and
- limited diversity in patient demographics.

## 4. Discussion

### 4.1. Principal Findings

There is a growing body of literature exploring the relationships of running on certain mental health outcomes. There were variations in methods and outcomes studied, but there were similar overall beneficial trends. Generally, evidence supported positive effects of a range of lengths and intensities of running on mental health. However, there was limited diversity in participant demographics. Attribution was also compromised by the limited number of studies with comparisons/control groups. Synthesis of quantified effects is made challenging by large variations in reporting methods. Consistency and appropriateness of mental health measures was also varied throughout the literature.

The review identified a smaller evidence-base focused on clinical populations. Behaviour change and compliance can be challenging in populations with clinical depressive disorders [145], and there is limited evidence regarding the long-term impact of PA in the treatment of depression [7,146,147]. Further investigations of the effects of running in populations with prior diagnoses of mental health disorders may help to address the global burden of mental illness.

### 4.2. Plausible Explanations for Findings

Our findings suggest that, throughout cross-sectional evidence, acute bouts of running and longer-term running interventions are associated with improvements in a range of mental health outcomes. This is likely explained by running supplying a sufficient dose of moderate to vigorous PA to stimulate the known mental health benefits associated with PA. These benefits are thought to be mediated by neurobiological, psychosocial and behavioural mechanisms, all of which an effective running intervention of any genre has the potential to influence [148]. The differential effects of these mechanisms remain unclear and may explain the variation in findings by running duration, intensity, setting, and social or individual participation.

### 4.3. Comparison to Literature

This review does not present running and mental health as a novel idea. As early as 1979, scholars discussed the relationship between psychotherapy and running [149]. An early review by Vezina et al. (1980) reported that regular running causes positive mood changes, increases self-esteem and decreases anxiety [150]. Another review by Hinkle (1992) found positive psychological effects in both adults and children including reductions in depressive mood and anxiety, and enhanced self-esteem [151]. However, a review by Weinstein et al. (1983) found that the volume of literature examining running and depression was scarce, and while running appeared to improve a sense of well-being, there was minimal evidence to strongly support reductions in depression and anxiety [152].

Studies from 1986 [153] and 1991 [154] warned that long-distance running had the potential to trigger development of eating disorders in people who were psychologically or biologically at risk. Early research also highlighted that runners should be aware of the possibility of addiction [155] and that women may be linked more strongly to negative addiction than men [156].

This review agrees with these earlier findings but is the first to use systematic scoping review methods. This means that it presents a transparent search and inclusion strategy and is less prone to bias in terms of included studies and resulting findings. As such, this review has contributed to the evidence base by demonstrating that the weight of evidence up to 2019 favours positive mental health relationships with running.

### 4.4. Strengths and Limitations

The authors acknowledge the limitation that this review was designed to assess the behaviour of running but that there are fields of studies including treadmill-based exercise which our review may not have picked up. However, the strength of this review is that the review does not focus on laboratory-based exercise but instead on what a healthcare professional may recommend to a free-living patient or the general public for mental health benefits. However, subjective measures of running intensity were not considered in detail, which may impact the conclusions of the review. The authors acknowledge that the results were not separated by means of running type due to the method of prioritization used to report the results, and thus, this remains a research gap. As with any scoping review, it is possible that the search and inclusion strategy led to omission of some key research.

Synthesis of quantified effects was also made challenging by the large range of reporting methods used within the studies. This scoping review did not attempt to undertake quality appraisal of the included studies. The wide range of study designs and methods included within the review does not allow a statistical synthesis of the effectiveness of the studies.

### 4.5. Implications

Pharmacological management is often used as a first-line of defence for mental health disorders [157]; however, it is not always effective due to poor adherence and relapse [158]. Ineffective management adds to the global burden of poor mental health [159]^,^ With increasing pressures on healthcare budgets, PA offers an augmentative therapeutic option for mental health management [160]. It is likely that using a cost-effective therapy such as running to improve mental health would prove economical as well. An integrated lifestyle intervention (i.e., iterative process) may be more feasible than a single add-on exercise intervention (i.e., addition of an individual behaviour) for patients with major depressive disorder who are deemed suitable for running therapy by clinicians.

This review presents the effects of running on mental health and can inform healthcare professionals and psychologists who advise on management of mental health conditions. The authors’ interpretation of the evidence base is that, with appropriate clinical judgement, practitioners may identify patients with an interest in running or previous history of running as an ideal candidate for running as a form of psychotherapy. Findings from this review indicate that characteristics of running to be recommended may include self-pacing, distance and time feasibility to the individual, and being within the lactate threshold. There were consistent trends within findings despite a variety of running interventions, which suggest that it would be appropriate to recommend track running, outdoor urban and rural running, and treadmill running to improve mental health. However, a large number of studies used healthy, active college-aged participants, which may limit the relevance of these recommendations to other population groups. It is acknowledged that running will not be a suitable recommendation for everyone and that prescription of running is not as simple as just instructing people to run; it will require clinical expertise with regard to mental health in the way it is prescribed [161].

### 4.6. Future Research

This review identifies research gaps regarding patient demographics, but we have further recommendations about increasing sample sizes, quantitative study design and more coherent mental health outcomes. There was great variability in mental health outcome measures, particularly within the acute bout studies, where short-term measures of mental health could have equally been defined as mood and affect. We recommend that future research seeks more clarity on appropriate outcome measures. A comparison of types, settings and intensities of running is needed to better inform running and mental health recommendations.

Recommendations for future research include addressing the effect of running on mental health of those under 18, those over 50s and clinical populations. A meta-analysis of the subset of study types such as interventions should be carried out. While the appropriateness of running interventions in those over 50 may be questioned, there is evidence that older adults do also benefit from the anti-depressive effect of exercise [162]. We know that children running can be used as a population intervention, for example, in “The Daily Mile” [163], which signifies the importance of addressing this gap around the mental health impact of running in those under 18. Future systematic reviews and meta-analyses are needed to quantify the benefits of running on specific outcomes.

## 5. Conclusions

This review is the most recent to comprehensively report the breadth of literature on the relationship between running and mental health. We conclude that running has important positive implications for mental health, particularly depression and anxiety disorders, but synthesis of quantified effects is made challenging by variation in reporting methods and remains a gap. This scoping review may have consequences for researchers, practitioners and relevant organisations and may inform the practice of healthcare professionals. Knowledge gaps concerning running on the mental health of children, older adults and clinical populations provide guidance for future research

## Figures and Tables

**Table 1:** Definitions of the mental health outcomes included within the review: the outcomes were informed by Kelly et al. (2018) [8].

Outcome	Description
Depression	Depression is a mood disorder with prolonged periods of low mood and a lack of interest and/or pleasure in normal activities most of the time. This includes major depressive disorder [19].
Anxiety	Anxiety is characterised by uncomfortable or upsetting thoughts and is usually accompanied by agitation, feelings of tension and activation of the autonomic nervous system. It is important to note the distinction between transient anxiety symptoms (state anxiety), persistent symptoms (trait anxiety) and anxiety disorders: a collection of disabling conditions characterised by excessive, chronic anxiety. Examples of anxiety disorders are specific phobias, social phobia, generalised anxiety disorder, panic disorder, obsessive–compulsive disorder and post-traumatic stress disorder [20].
Self-efficacy	Self-efficacy is a situation-specific form of self-confidence. Self-efficacy beliefs influence how people think, feel, motivate themselves and act [21].
Psychological stress	Psychological stress or distress can be defined as the unique discomforting, emotional state experienced by an individual in response to a specific stressor or demand that results in harm, either temporary or permanent, to that person [22].
Eating pathology	Eating pathology or disorder can be described as persistent disturbance of eating behaviours or behaviours intended to control weight, which significantly impairs physical health or psychosocial functioning. This disturbance should not be secondary to any recognised general medical disorder, e.g., hypothalamic tumour. This definition includes anorexia nervosa and bulimia nervosa [23].
Self-esteem	Self-esteem is the feelings of value and worth that a person has for oneself. It contributes to overall self-concept as a construct of mental health [24].
Addiction	Addiction designates a process whereby a behaviour that can function both to produce pleasure and to provide escape from internal discomfort is employed in a pattern characterized by (1) recurrent failure to control the behaviour (powerlessness) and (2) continuation of the behaviour despite significant negative consequences (unmanageability) [25].
Psychological well-being	Psychological well-being links with autonomy, environmental mastery, personal growth, positive relations with others, purpose in life and self-acceptance. This is often referred to as eudemonic well-being [26].
Self-concept	Self-concept is the organisation of qualities that the individual attributes to themself, which in turn guides or influences the behaviour of that individual [27].
Mood	Mood is a transient state of a set of feelings, usually involving more than one emotion. Seen as a conscious summative recognition of feelings that can vary in intensity and duration [28].

**Table 2:** Summary of data extraction from the 47 cross-sectional studies.

Author	Year	Country	Design	Population	Mental Health Outcome (Measurement)	Study Aim	Main Findings
Wilson et al. [29]	(1980)	Canada	Cross-sectional	*n* = 30, all male; age range 20–45; 10 marathoners, 10 regular joggers and 10 non-exercisers	Mood (Profile of Mood States)	Comparing mood states of marathon runners, regular joggers and non-exercisers	Marathoners and joggers reported less depression (F_(2,28)_ = 7.51, *p <* 0.003), anger (F = 10.11, *p* < 0.001) and confusion (F = 12.41, *p* < 0.001) and more vigour (F = 103.21, *p* < 0.001) than non-exercisers. Marathoners reported less fatigue (F = 10.26, *p* < 0.001) and tension (F = 7.51, *p* < 0.003) than non-exercisers. Marathoners and joggers did not significantly differ on reported fatigue and tension; however, marathoners had significantly less depression, anger and confusion but more vigour than joggers.
Joesting [30]	(1981)	USA	Controlled cross-sectional	*n* = 100 runners; 79 males, mean age 18.36; 21 females, mean age 16.53	Depression (Depression Adjective Checklist)	Investigating the relationship between running and depression	Significantly (*p* < 0.01) decreased depression in males and female runners compared to Lubin’s data for nonpsychiatric patients: male and female runners mean depression scores were 4.59 and 4.33, respectively, while the normative nonpsychiatric sample means were 8.02 and 7.32, respectively.
Jorgenson et al. [31]	(1981)	USA	Cross-sectional	*n* = 454 regular runners; 390 males and 64 females; majority aged 30–39	Emotional well-being (structured questionnaire consisting of 55 items designed by the author)	Investigating the relationship between emotional well-being and running	Of the runners, 92.3% (*n* = 419) indicated an increase in emotional well-being (*p* < 0.01) but no report on the scale of improvement. Age and emotional well-being were significantly correlated (gamma value = 0.42, *p* < 0.001), with the older runner having the greater perception of emotional well-being resulting from running. There was a significant inverse relationship between average hours per week running and emotional well-being (gamma value = −0.43, *p* < 0.001).
Valliant et al. [32]	(1981)	Canada	Cross-sectional	*n* = 68 male runners; 30 marathon runners, mean age 34.4; 38 recreational runners, mean age 20.6	Self-sufficiency and personality profiles (a 1-h “Sixteen Personality Factor Questionnaire”)	Comparing self-sufficiency and personality profiles in marathon runners vs. recreational joggers	Marathon runners had a more self-sufficient personality compared to joggers who were less assertive and more conscientious and had controlled personality types: On average, marathoners more reserved (F = 17.07, df = 1,66, *p* < 0.001), intelligent (F = 12.69, df = 1,66, *p* < 0.001), tender-minded (F = 11.79, df = 1,66, *p* < 0.001), imaginative (F = 11.09, df = 1,66, *p* < 0.005) and self-sufficient (F = 19.84, df = 1,66, *p* < 0.001) than joggers. Conversely, joggers were more happy-go-lucky (F = 10.05, df = 1,66, *p* < 0.005), apprehensive (F = 10.51, df = 1,66, *p* < 0.005) and controlled (F = 7.09, df = 1,66, *p* < 0.01).
Francis et al. [33]	(1982)	USA	Cross-sectional	*n* = 44 male participants; mean age 32; non-running controls who ran 0 miles weekly (*n* = 16), 20 miles (*n* = 10), 30–40 miles (*n* = 8) and 50–60 miles (*n* = 10)	Anxiety, depression and hostility (State-Trait Anxiety Inventory and the Multiple Affect Adjective Check List)	Comparing anxiety, depression and hostility in various groups of runners vs. sedentary controls	Compared to sedentary controls, runners had lower anxiety (4.2 vs. 7.2, *p* < 0.01), depression (8.6 vs. 12.3, *p* < 0.01) and hostility (4.8 vs. 6.8, *p* < 0.01).
Hailey et al. [34]	(1982)	USA	Cross-sectional	*n* = 60 male runners; aged 13–60; Those who ran for less than 1 year (*n* = 12), those who ran for 1–4 years (*n* = 32) and those who ran for over 4 years (*n* = 16)	Negative addiction (Negative addiction scale)	Investigating the relationship between running and negative addiction	The more years that a male had been running, the greater the risk of developing negative addiction (F_(2,58)_ = 3.48, *p* < 0.05). Runners with a running history of <1 year scored a mean of 3.84 (scale of 1–14), those running for 1–4 years scored 5.63 and those running for 4+ years scored 6.38. Addiction scores for runners of 4+ years was greater than the addiction score for runners of <1 year (t_(59)_ = 2.72, *p* < 0.005). Likewise, the addiction score for runners of between 1–4 years was greater than the score for runners <1 year (t_(59)_ = 2.52, *p* < 0.01). No statistically significant difference in addiction scores were found between the 1–4-year group and the 4+ year group.
Callen [35]	(1983)	USA	Cross-sectional	*n* = 424 non-professional runners who ran on average more than 28.8 miles per week; 303 males and 121 females; mean age 34	Mental and emotional aspects (a questionnaire designed by the author)	Investigating mental and emotional aspects associated with long-distance running in non-professional runners, including depression, tension, mood, happiness, self-confidence and self-image	Ninety-six percent of subjects noticed mental/emotional benefits from running, but none reported the size of benefits. Benefits included relief of tension (86% of all respondents, *n*.s.), improved self-image (77%, *n*.s.), better mood (66%, *p* < 0.05), improved self-confidence (64%, *n*.s.), relieved depression (56%, *p* < 0.05) and improved happiness (58%, *n*.s.). However, 25% stated they had experienced emotional problems associated with running, with almost every instance being a problem of depression, anger or frustration associated with not being able to run due to injury, but no details of size or significance were reported. Sixty-nine percent of runners experienced an emotional “high” while running.
Galle et al. [36]	(1983)	USA	Controlled cross-sectional	*n* = 391 female subjects; aged 15 to 50; runners (*n* = 102), infertility patients (*n* = 103), fertile subjects (*n* = 139) and Clomid study patients whose only infertility abnormality was ovulation dysfunction (*n* = 47)	Anxiety and depression (Hopkins Symptom Checklist-90)	Comparing psychologic profiles including anxiety and depression in runners, infertility patients, fertile subjects and Clomid study patients whose only infertility abnormality was ovulation dysfunction	Emotional distress scores of runners were not significantly different from the fertile control subjects (F = 1.19, ns), but both groups of infertility patients showed greater distress on items in the depression subscale than the runners and fertile control subjects (F = 3.42, *p* < 0.025). The only significant difference between runners and fertile control subjects was that control subjects had higher hostility (*p* < 0.05). Regarding just runners, there was significant differences in depression between amenorrhoeic (*n* = 15) and regular cycling runners (*n* = 87), with amenorrhoeic runners scoring higher in the depression factor than regular ovulation cycle runners (F = 3.0, *p* < 0.10).
Lobstein et al. [37]	(1983)	USA	Pre-post controlled between subject design	*n* = 22 medically healthy men; 11 physically active men and 11 sedentary men; aged 40–60	Depression (Minnesota Multiphasic Personality Inventory)	Assessing the impact of a treadmill run with increasing gradient on depression	Sedentary men were significantly more depressed than men who ran (mean = 61.36 vs. 50.73, respectively, *p* < 0.01), but both groups were within clinical limits for normal, mentally healthy, middle aged men.
Rudy et al. [38]	(1983)	USA	Cross-sectional	*n* = 319 female regular runners; aged between 16 and 60	Anxiety and self-esteem (Rosenberg Self-esteem Scale and Zuckerman’s Anxiety Adjective Checklist)	Investigating how levels of anxiety and self-esteem related to intensity of jogging	Female runners jogging with great intensity had significantly less anxiety than lower intensities (x^2^ = 22.83; *p* < 0.001). Results indicate that intensity of jogging influences self-esteem but was not significant: 89% of women scored in the range of high self-esteem, and in the open-ended answers, 29% of responses stated that they feel better about themselves, 12% had increased self-confidence and 6% stated a sense of accomplishment.
Goldfarb et al. [39]	(1984)	USA	Cross-sectional	*n* = 200 distance runners; 136 males and 64 females	Anorexia nervosa traits (Goldfarb Fear of Fat scale and Activity Vector Analysis)	Investigating anorexia nervosa traits within distance runners	Runners had a mean score of 2.91 (on a 10-point scale), indicating a low–normal fear of fat, and only 29 (14.5%) participants reported a high fear-of-fat score (score between 6 and 10). Fear-of-fat scores did not correlate significantly with measures of running zealousness: miles run per week (r = −0.04), number of workouts per week (r = 0.09), number of road races (r = 0.05), marathons completed (r = −0.05) or degree of importance placed on running (r = −0.03). Runners who demonstrated the greatest zealousness demonstrated Activity Vector Analysis profiles that clustered around one particular profile (r = 0.64, *p* < 0.05) indicating assertive, obsessive, perfectionistic and anxious individuals. Results do not support a correlation between running and fear of fat; however, runners most closely resembling “obligatory runners” exhibited traits characteristic of anorexia nervosa patients.
-Guyot et al. [40]	(1984)	USA	Controlled cross-sectional	*n* = 126 participants; 64 runners (44 males and 20 females) vs. 62 non-runners (37 males and 25 females)	Death anxiety (Death Concern Scale)	Comparing death anxiety in runners vs. non-runners	Runners experienced more death thoughts (F_(1,122)_ = 4.49, *p* < 0.05) but less death anxiety (F_(1,122)_ = 6.35, *p* < 0.05) than non-runners.
Rape [41]	(1987)	USA	Controlled cross-sectional	*n* = 42 male participants; aged 18–25; 21 runners vs. 21 non-exercisers	Depression (Beck Depression Inventory)	Comparing depression scores in runners vs. non-exercisers	Runners were significantly less depressed (M = 4.38, SD = 3.88) than the non-exercisers (M = 9.55, SD = 5.40) (t_40_ = 3.55, *p* < 0.001). Overall results suggest that running reduces depression.
Weight et al. [42]	(1987)	South Africa	Controlled cross-sectional	*n* = 135 female participants consisting of marathon runners (*n* = 85) vs. cross country runners (*n* = 25) vs. non-running controls (*n* = 25); aged 18–56	Eating attitudes and disorders (Eating Attitudes Test and the Eating Disorder Inventory)	Comparing eating attitudes and disorders in marathon runners vs. cross country runners vs. non-running controls.	No significant differences were found between groups on any of the Eating Attitudes Test scores (mean scores = 8.4, 14.3 and 11.8). Eating Disorder Inventory scores also did not follow a definite pattern (mean scores for marathoners, cross country runners and non-running controls were 24.8, 27.1 and 32.0, respectively), indicating that abnormal eating attitudes and the incidence of anorexia was no more common among competitive female runners than among the general population, with a low incidence of anorexia in the total group (2 out of 135 participants).
Chan et al. [43]	(1988)	USA	Cross-sectional	*n* = 60 runners who ran at least 3× per week for a minimum of a year; 28 males and 32 females; prevented runners *n* = 30 vs. continuing runners *n* = 30; aged 15–50.	Depression, self-esteem and mood (Zung depression Scale, Rosenberg Self-esteem Scale and Profile of Mood States)	Comparing depression, self-esteem and mood in prevented runners vs. continuing runners	Prevented runners reported significantly greater overall psychological distress (Wilks’s = 0.63, *p* < 0.01: X_9_^2^ = 24.38, *p* < 0.01), depression (F_(1,58)_ = 11.57, *p* < 0.01) and overall mood disturbance (F_(1,58)_ = 11.03, *p* < 0.01) than continuing runners. Prevented runners reported significantly lower self-esteem (F_(1,58)_ = 3.17, *p* < 0.05), less satisfaction with the way their bodies’ present looks (F_(1,58)_ = 4.17, *p* < 0.05) and had greater desire to change something about the way their bodies look (F_(1,58)_ = 4.54, *p* < 0.05) compared to continuing runners.
Frazier [44]	(1988)	USA	Post only, nonrandomised long-term observational study	*n* = 86 regular, distance runners who had all completed a marathon; 68 males with mean age of 33.7 and 18 females with a mean age of 32.2	Mood (Profile of Mood States)	Investigating the relationship between running and mood in regular distance runners	Results suggest that regular, distance running improves mood in both males and females. Running subjects had lower mean scores on tension, anger, depression, fatigue and confusion and a higher mean of vigour compared to scores for test norms; however, statistical significance was not reported here. The only significant difference between males and females was on the confusion subscale: female mean = 7.8 vs. male = 5.5 (F_(1,84)_ = 5.33, *p* < 0.05).
Lobstein, Ismail et al. [45]	(1989)	USA	Controlled cross-sectional	*n* = 36 male participants; aged 40–60; runners (*n* = 21) vs. sedentary controls (*n* = 15)	Anxiety and depression (Minnesota Multiphasic Personality Inventory and Eysenck Personality Inventory)	Comparing anxiety and depression levels in runners vs. sedentary controls	Overall, running reduced anxiety (mean = 48.95 vs. 61.48 respectively, *p* < 0.05, standardised canonical coefficient = −1.07) and depression (mean = 50.76 vs. 57.93, respectively, *p* < 0.05, standardised canonical coefficient = 0.00) compared to being sedentary.
Lobstein, Rasmussen et al. [46]	(1989)	USA	Controlled cross-sectional	*n* = 20 psychologically normal, medically healthy men; aged 40–60; physically active joggers (*n* = 10) vs. sedentary (*n* = 10)	Depression and stress (Eysenck Personality Inventory and Minnesota Multiphasic Personality Inventory)	Comparing depression and stress in sedentary men to physically active joggers	The findings suggest that regular jogging increases emotional stability (t = −2.84, *p* < 0.01) and decreases subjective depression with MMPI subscales of depression and Wiggins depression both being significantly lower in the joggers (*t* = 3.70, *p* < 0.01; *t* = 2.40, *p* < 0.05; respectively).
Nouri et al. [47]	(1989)	USA	Cross-sectional	*n* = 100 male participants; aged 18–62; non-exercisers (*n* = 28), drop-out joggers (*n* = 21), beginning joggers (*n* = 15), intermediate joggers (*n* = 16) and 20 advanced joggers (*n* = 20)	Anxiety and addiction/commitment (State-Trait Anxiety Inventory, Commitment to Running Scale, and The Buss–Dutkee Inventory measuring hostility and aggression)	Investigating the relationship between various levels of jogging vs. non-exercising on anxiety and addiction/commitment	Running reduced anxiety levels compared to physical inactivity (F_(4,89)_ = 4.43, *p* < 0.01), with advanced joggers scoring significantly lower on trait anxiety than beginner and intermediate joggers (1.42 vs. 1.77 vs. 1.69, respectively, *p* < 0.01) and commitment to running significantly higher for the joggers than the non-exercisers (F_(4,89)_ = 14.30, *p* < 0.01).
Chan et al. [48]	(1990)	Hong Kong	Cross-sectional	*n* = 44 male runners of track clubs who ran a mean of 57.2 km per week; mean age 27.8	Depression, stress, tension and personality profiles (Chinese version of the Personality Research Form)	Investigating the relationship between running and depression, stress, tension and personality profiles	Running increased mood, happiness and outlook, while relieving anger, depression and aggression, but none reported the size of changes or significance; 36.4% of participants reported “improving mental health” as a reason to start running. Emotional benefits from running reported were more self-confident (59.1% of respondents), happier (56.8%), better mood (50.0%), relieved tension (45.5%), better self-image (36.4%), relieved depression (36.4%), more aggression (36.4%), improved outlook (34.1%), more content (31.8%) and better family relationship (15.9%). However, when participants stopped running, 38.6% experienced low mood and 25.0% experienced anxiousness. More experienced runners, compared to less experienced runners, were less aggressive or easily angered (t = 2.92, df = 42, *p* < 0.01), less guarded or defensive (t = 2.13, df = 42, *p* < 0.005) and more likely to present themselves favourably (t = 2.68, df = 35, *p* < 0.05).
Chapman et al. [49]	(1990)	USA	Cross-sectional	*n* = 47 runners; 32 males aged 34–57 and 15 females aged 35 to 59	Running addiction, psychological characteristics and running (Running Addiction Scale, Commitment to Running Scale, Symptom Checklist (SCL-90-R) and Levenson’s Locus of Control Scale)	Investigating the relationship between running addiction, psychological characteristics and running	Results suggest a sex difference in the relationship between addiction and commitment, in that commitment to running can occur without addiction in females. Running Addiction Scale (RAS) scores correlated strongly for both sexes with self-rated addiction (*p* < 0.05) and moderately with discomfort (*p* < 0.05). However, the Commitment to Running Scale (CR) did not significantly correlate with self-rated addiction in females (0.246, ns) while RAS did (0.753, *p* < 0.05) (z = 2.00, *p* < 0.05). Running addiction was associated with a high frequency of running (*p* < 0.05) and longer duration of running (males = *p* < 0.05; females = ns). The CR score correlated significantly with run frequency for male (0.59, *p* < 0.05) but not female runners (0.14, ns), while CR and run duration did not correlate significantly for either sex (males = 0.16, females = 0.28, *n*.s.). Duration of running was associated with mood enhancement, implying that the benefits of running to mood may be obtained without addiction. Males were above the norm for obsessive–compulsive tendencies (SCL-90 score) and significantly higher than for females (*p* < 0.05), with running addiction associated with male-positive personality characteristics (*p* < 0.05) but not with mood enhancement. There were no significant correlations with personality traits for females.
Guyot [50]	(1991)	USA	Cross-sectional	*n* = 370 regular long-distance runners; 289 males, mean age 38; 81 females, mean age 35	Addiction and death anxiety (Dickstein Death Concern Scale and author-created questionnaires for pain running, running motives, risk taking and medical symptoms)	Investigating the relationship between addiction and death anxiety between pain runners and non-pain runners	Of the 370 runners, 56% pushed themselves during running until they felt pain. Compared to non-pain runners, pain runners were more likely to be male, taller (F_(1,366)_ = 11.45, *p* < 0.05), heavier (F_(1,366)_ = 9.19, *p* < 0.05) and younger (F_(1,366)_ = 5.75, *p* < 0.05). Overall, results suggest that runners classified as pain runners experienced significantly more death thoughts (F_(1,364)_ = 5.04, *p* < 0.05) and death anxiety (F_(1,364)_ = 8.86, *p* < 0.05) than non-pain runners.
Maresh et al. [51]	(1991)	USA	Cross-sectional	*n* = 29 male distance runners; mean age 40.1	Psychological characteristics including anxiety, depression and stress (Myers–Briggs Type Indicator Form and Multidimensional Anger-Inventory)	Investigating psychological characteristics including anxiety, depression and stress in distance runners	Results suggest that long-term involvement in running is associated with low levels of self-reported anxiety (m = 2.5 on a 6-point scale), depression (M = 1.8) and stress (m = 2.5). Runners’ personality profiles differed from the normative sample, suggesting that running is associated with more introverted personalities compared to men in the general population. Compared to a normative sample of male control students, runners were less angry overall, less frequently angry, and angry across fewer situations. However, 82% of runners reported withdrawal symptoms when forced to be inactive, with a self-reported addiction average of 4.4 (“moderately” to “very”) on a 6-point scale.
Gleaves et al. [52]	(1992)	USA	Controlled cross-sectional	*n* = 60 female participants; runners (*n* = 20), bulimia patients (*n* = 20) and a non-exercising, non-dieting control group (*n* = 20)	Depression, body image and bulimia nervosa symptomology (Beck’s Depression Inventory, Body Image Assessment Procedure, subscales from the Eating Disorder Inventory, Automatic thoughts Questionnaire and dieting/weight loss questionnaire)	Comparing depression, body image disturbance and bulimia nervosa symptomology in runners, bulimia patients and a non-exercising, non-dieting control group	No differences were found between runners and controls throughout the study. Bulimics had significantly higher depression scores than runners and controls (20.65, 3.30 and 4.80 respectively, F = 56.95, *p* < 0.0001), but runners and controls did not differ from each other. The same pattern of results was found for the Autonomic Thoughts Questionnaire (F = 45.87, *p* < 0.0001) and Eating Disorder Inventory (F = 34.95, *p* < 0.0001), with bulimics scoring higher, but no significant difference was found between runners and controls: ATQ means = 85.40, 41.10 and 41.50, respectively, and EDI means = 12.80, 0.80 and 1.60, respectively. There were significant group effects for all three variables of body image (*p* < 0.01); again, bulimics differed from runners and controls.
Coen et al. [53]	(1993)	USA	Cross-sectional	*n* = 142 male marathon runners; mean age 44.07; obligatory runners (*n* = 65) vs. non-obligatory runners (*n* = 77)	Anxiety, anorexia and self-identity (Obligatory Exercise Questionnaire, State-Trait Personality Inventory and The Ego Identity Scale)	Investigating the relationship between obligatory running vs. non-obligatory running on anxiety, anorexia and self-identity	Compared to the non-obligatory runners, the obligatory group ran significantly more miles per week,0 spent more time running each week (t_(140)_ = 13.19, *p* < 0.001) and had significantly higher levels of anxiety (18.85 vs. 6.45, respectively, (*p* < 0.01), suggesting that running represents a successful coping mechanism to reduce anxiety. There was no statistically significant difference in Ego Identity Scale score (*p* > 0.05), indicating that neither group showed a higher developed sense of identity.
Furst et al. [54]	(1993)	USA	Controlled cross-sectional	*n* = 188 participants, with *n* = 98 runners: 72 males and 26 females vs. *n* = 90 gym exercisers: 60 males and 30 females; majority aged 20–29	Negative addiction (Negative Addiction Scale)	Comparing negative addiction in runners vs. gym exercisers	A significant association was found between years of participation in physical activity and addiction scores (F_(5,182)_ = 6.39, *p* < 0.01) regardless of the type of activity, with no significant differences in addiction scores between runners and gym exercisers.
Masters et al. [55]	(1993)	USA	Cross-sectional	*n* = 712 participants in a marathon; 601 males and 111 females; aged 16–79	Self-esteem and psychological coping of runners (Motivation of Marathoners Scales, Sport Orientation Questionnaire, Marlowe–Crowne Social Desirability Scale, Attentional Focusing Questionnaire, and 3 body satisfaction and composition questions)	Investigating self-esteem and psychological coping of marathon runners	Participation in marathon running and training was used as a way to problem solve with self-distraction for psychological coping (r(66) = 0.54, *p* < 0.001), improving self-esteem (r(66) = 0.31, *p* < 0.01) and life meaning (r(66) = 0.36, *p* < 0.01). Marathon runners reporting higher anxiety levels were more likely to endorse psychological motives for marathon running, indicating that their running helps them avoid or dampen negative emotional experiences: psychological coping (r(62) = 0.38, *p* < 0.01) and self-esteem (r(62) = 0.36, *p* < 0.01). Women more strongly endorsed weight concern as a reason for involvement in marathons (t_(588)_ = −3.52, *p* < 0.001). Personal goal achievement and competition were both positively related to training miles per week (r(575) = 0.22, *p* < 0.001 and r(576) = 0.30, *p* < 0.001, respectively).
Pierce et al. [56]	(1993)	USA	Cross-sectional	*n* = 89 male runners; *n* = 33 non-competitive runners vs. *n* = 24 5-km runners vs. *n* = 32 marathoner runners	Exercise dependence (negative addiction scale)	Comparing exercise dependence in recreational (non-competitive) runners vs. 5-km runners vs. marathoner runners	Training mileage was significantly correlated with exercise dependence, with marathoners showing significantly higher (*p* < 0.05) mean exercise dependence scores (3.78) compared to 5K (2.9) and recreational runners (2.16). There was no significant difference in exercise dependence scores found between recreational and 5K runners.
Klock et al. [57]	(1995)	USA	Controlled cross-sectional	*n* = 22 females who were not currently pregnant or taking oral contraceptives; amenorrhoeic runners (*n* = 7, mean age 28.0), eumenorrheic runners (*n* = 9, mean age 32.1) and eumenorrheic sedentary women as controls (*n* = 6, mean age = 27.5)	Depression, anorexia nervosa, excessive exercise and eating disorder (The modified Body Image Questionnaire, the Beck Depression Inventory, the Symptom Checklist-90 and the Eating Disorders Inventory)	Comparing depression, anorexia nervosa, excessive exercise and eating disorders in amenorrhoeic runners, eumenorrheic runners and eumenorrheic sedentary women as controls	No significant differences were found between amenorrhoeic runners, eumenorrheic runners and eumenorrheic sedentary controls on any of the psychological measures; hence, these results do not suggest that there are psychological similarities between obligatory runners and anorexics. However, there was a subgroup of amenorrhoeic runners (3 out of 9) who scored in the clinically depressed range on the BDI, indicating that they were mild to moderately depressed, and who also had the highest scores in their group on the Eating Disorder Inventory measures.
Thornton et al. [58]	(1995)	UK	Cross-sectional	*n* = 40 long-standing, habitual male runners who ran on average 4 times per week with a weekly mileage of 42.5 miles; mean age 38	Addiction (Rudy and Estok Running Addiction Scale, the Hailey and Bailey Running Addiction Scale, and the Personal Incentives for Exercise questionnaire)	Investigating a relationship between habitual running and addiction	A high level of commitment in runners was found, with 55% classified as moderately committed (scores 13–20) and 22% classified as highly “addicted” (scores +20), but no relationship between years of running and addiction scales was found. This contrasts the significant correlations between both the Estok RAS and frequency of running (r_s_ = 0.38; *p* < 0.05) and the Bailey RAS and number of runs per week (r_s_ = 0.55; *p* < 0.01). The correlation between the two addiction scales revealed a strong positive relationship (r_s_ = 0.81; *p* < 0.001). The primary motivation for running was mastery (mean Personal Incentives for Exercise score of 4.2), followed by competition (3.93), weight regulation (3.9), health benefits (3.89), fitness (3.87) and social recognition (3.01).
Powers et al. [59]	(1998)	USA	Controlled cross-sectional	*n* = 57; habitual male runners (*n* = 20), habitual female runners (*n* = 20) and female anorexia nervosa patients (*n* = 17)	Psychological profiles (Minnesota Multiphasic Personality Inventory, Leyton Obsessional Inventory, Obligate Running Questionnaire, Becks Depression Inventory and three body image tests)	Comparing psychological profiles of habitual male runners, habitual female runners and female anorexia nervosa patients	Significant differences in body image between groups (F = 7.969, *p* < 0.001) were found, but no significant differences between female groups were found. Anorexics scored higher than either group of runners (*p* < 0.001) for MMPI subscales of depression, hysteria and psychopathic deviate, while none of the mean scores for either set of runners were considered clinically significant. Anorexics scored higher on the Becks Depression Inventory than both male and female runners (F = 68,645, *p* < 0.0001, mean scores = 23, 2.4 and 3.45, respectively), but again, there was no significant differences between the runners. While there were suggestive similarities between female runners and anorexics on body image, the overall results found few psychological similarities between anorexia patients and habitual runners, with evidence of significant psychopathology on all psychological measures in the anorexia group, while both groups of runners were consistently in the normal range.
Slay et al. [60]	(1998)	USA	Cross-sectional	*n* = 324 regular runners; 240 males and 84 females; 84 classified as obligatory runners: 63 males and 21 females; ages 15–71	Eating pathology traits (Eat Attitudes Test, and Obligatory Running and Motivations for Running Questionnaire)	Comparing eating pathology traits between obligatory and non-obligatory runners	Obligatory runners, particularly females, are most at risk of eating pathophysiology, as obligatory runners scored significantly higher on the EAT test, with female obligatory runners having the highest mean EAT score (r = 0.40, *p* < 0.0002). At low levels of obligatory running, women and men scored similarly on the EAT test (F_(1,164)_ = 2.78, *p* > 0.05); however, at higher levels of obligatory running, women demonstrated significantly higher EAT scores than men (F_(1,164)_ = 29.50, *p* < 0.001).
Ryujin et al. [61]	(1999)	USA	Controlled cross-sectional	*n* = 55 female participants; collegiate distance runners (*n* = 20) vs. non-running undergraduate student controls (*n* = 35)	Eating disorder symptomology (Eating Disorders Inventory 2)	Comparing eating disorder symptomology in collegiate distance runners to non-running undergraduate student controls	Distance runners showed no enhanced symptomatology of eating disorders; instead, female distance runners exhibited fewer symptoms of eating disorders on all subscales of the Eating Disorder Inventory-2 except Perfectionism: drive for thinness (t_(107)_ = 3.34, *p* < 0.005), bulimia (t_(107)_ = 2.48, *p* < 0.05) and body dissatisfaction (t_(107)_ = 4.23, *p* < 0.001).
Leedy [62]	(2000)	USA	Controlled cross-sectional	*n* = 276 runners with an average of 11.5 years of running experience; 239 men, mean age 37.9; 37 women, mean age 40.5	Depression and anxiety (Diagnostic and Statistical Manual-IV, and an author-adapted scale based on the Running Addiction Scale)	Comparing depression and anxiety in runners to non-runners	Of the non-runners and runners, 16.2% and 4.6%, respectively, had been diagnosed with an anxiety disorder or prescribed an anxiolytic medication. These participants had significantly higher anxiety trait scores than those without a diagnosis: F_(1,274)_ = 18.87, *p* < 0.0001; 27% of non-runners and 11.8% of runners reported a diagnosis of depression or were prescribed an antidepressant. These participants had significantly higher measures of depression traits: F_(1,274)_ = 22.46, *p* < 0.0001. Women’s Stress Relief scores were significantly higher than men’s (F_(1,229)_ = 20.51, *p* < 0.001). Stress relief scores also varied across race length (F_(2,229)_ = 6.47, *p* < 0.005), indicating that runners entered in the 5–10K runs had lower scores than those running the half/full marathon. Results indicate that highly committed runners (*n* = 31) had significantly lower anxiety (F_(2,113)_ = 5.73, *p* < 0.01) and depression scores (F_(2,113)_ = 8.00, *p* < 0.001) than recreational runners (*n* = 46) and non-runners (*n* = 39).
Edwards et al. [63]	(2005)	South Africa	Cross-sectional	*n* = 277 participants; 94 males and 183 females; mean age 25.2; hockey players (*n* = 60), runners (*n* = 40) and health club gym members (*n* = 69) vs. a control group of non-exercisers (*n* = 108)	Psychological well-being and physical self-perception (Ryff’s Short Standardized 18-item scale of Objective Psychological Well-being, Fox’s Physical Self-Perception Profile (PSPP) and the Physical Self-Perception Profile)	Comparing psychological well-being and physical self-perception in hockey players, runners and health club gym members vs. a control group of non-exercisers.	All three forms of physical activity were associated with significantly higher (*p* < 0.01) scores on 11 out of the 15 dimensions of psychological well-being and physical self-perception scales compared to the control group: autonomy (F = 11.3), personal growth (F = 35.4), environmental mastery (F = 9.6), purpose in life (F = 149.2), positive relations with others (F = 81.6), self-acceptance (F = 50.4), sport competence (F = 41.3), conditioning (F = 28.1), sport importance (F = 11.7), conditioning importance (F = 28.1) and body importance (F = 31.0).
Schnohr et al. [64]	(2005)	Denmark	Observational cohort study	*n* = 12,028 participants; 5479 males and 6549 females; aged 20–79	Stress (An author-created questionnaire)	Comparing stress levels between jogging and various levels of physical (in)activity in leisure time	Those who were vigorously physically active (joggers) had the lowest level of stress compared to those with low activity levels (males, 3.1% vs. 12.8%, respectively; female, 3.3% vs. 19.3%, respectively). With increasing physical activity in leisure time, there was a decrease in level of stress between sedentary persons and joggers (Odds Ratio (OR) = 0.30) and a decrease in life dissatisfaction between sedentary persons and joggers (OR = 0.30). The highest levels of stress and dissatisfaction was seen in sedentary persons who remained inactive at follow-up, while the group that changed from sedentary to active had an adjusted OR < 0.50.
Strachan et al. [65]	(2005)	Canada	Prospective longitudinal study	*n* = 67 regular runners; 32 were male and 35 were female; mean age of 40.6	Self-efficacy and self-identity (author-created measures of task self-efficacy and self-regulatory efficacy, and a 10-item, validated athletic identity measurement scale)	Investigating the relationship between running and self-efficacy and self-identity	Significant comparisons were made between extreme self-identity groups on social cognitive and behavioural variables (F_(5,37)_ = 4.72, *p* < 0.002), with those higher in self-identity scoring higher on task self-efficacy (*p* < 0.001), scheduling self-efficacy (*p* < 0.03), running more frequently (*p* < 0.001) and running for longer durations (*p* < 0.005) than those who scored lowest on self-identity. Both scheduling self-efficacy (R^2^ change = 0.16, *p* < 0.001) and barriers to self-efficacy (R^2^ change = 0.22, *p* < 0.001) were correlated with self-identity to prospectively predict running frequency (F_(2,64)_ = 9.98, *p* < 0.001; F_(2,63)_ = 12.89, *p* < 0.001, respectively). Both task self-efficacy (R^2^ change = 0.06, *p* < 0.05) and self-identity (R^2^ change = 0.06, *p* < 0.04) were significant predictors of maintenance duration.
Galper et al. [66]	(2006)	USA	Retrospective cross-sectional	*n* = 6728 participants; 5451 males with a mean age of 49.5 and 1277 females with a mean age of 48.1; inactive (*n* = 1454 men and *n* = 422 women), insufficiently active (*n* = 1892 men and *n* = 443 women), sufficiently active (*n* = 1396 men and *n* = 283 women) and highly active (*n* = 709 men and *n* = 129 women)	Depression and emotional well-being (Center for Epidemiological Studies Scale for Depression and the General Well-Being Schedule)	Assessing retrospectively if the level of walking/running impacted depression and emotional well-being	Significant inverse association between increased physical activity and reduced depression scores for both men (F_(6, 5306)_ = 20.93, *p* < 0.0001) and women (F_(6, 1247)_ = 11.80, *p* < 0.0001) and a positive association between increased physical activity and increased well-being scores in men (F_(6, 5306)_ = 78.65, *p* < 0.0001) and women (F_(6, 1247)_ = 24.82, *p* < 0.0001) were found. These effects peaked at 11–19 miles per week (the sufficiently active category).
Luszcynska et al. [67]	(2007)	UK	Longitudinal prospective cohort study	*n* = 139 runners; 111 males and 29 females; mean age of 29.5; strong (*n* = 72) and weak (*n* = 66) maintenance self-efficacy, strong (*n* = 72) and weak (*n* = 61) recovery self-efficacy, and strong (*n* = 87) and weak (*n* = 45) intentions	Self-efficacy and running behaviour (an author-created questionnaire)	Investigating the relationship between self-efficacy and running behaviour with data collected twice with a time gap of 2 years	Participants decreased the frequency of running sessions after 2 years, regardless of baseline intensions or self-efficacy; however, those with stronger recovery in self-efficacy jogged more than those with weaker recovery in self efficacy 2 years later. All participants reduced the number of jogging or running sessions over 2 years (F_(1,131)_ = 43.43, *p* < 0.001); however, those with strong baseline recovery self-efficacy ran/jogged more often at 2 years than those who had weak recovery self-efficacy at baseline (F_(1,131)_ = 6.12, *p* < 0.05). Participants reduced the number of running or jogging sessions over the 2 years, regardless of strong or weak intentions at baseline (F_(1,130)_ = 34.55, *p* < 0.001) or of strong or weak baseline maintenance of self-efficacy (F_(1,130)_ = 42.12, *p* < 0.001). No effects of maintenance self-efficacy were found. Recovery self-efficacy at T1 predicted recovery self-efficacy (*p* < 0.05), maintenance self-efficacy (*p* < 0.05), and jogging or running behaviour (*p* < 0.05) assessed 2 years later. Overall, social-cognitive variables predicted behaviour, whereas behaviour did not predict social-cognitive variables.
Smith et al. [68].	(2010)	UK	Cross-sectional	*n* = 93 non-competitive, regular runners; 47 males and 46 females	Exercise dependence, running addiction and social physique anxiety (Exercise Dependence Scale, Running Addiction Scale and Social Physique Anxiety Scale)	Comparing exercise dependence, running addiction and social physique anxiety in male vs. female runners	While a significant proportion of runners displayed symptoms of exercise dependence, results did not find that exercise dependence was linked to social physique anxiety (F_(3.179)_ = 1.21, *p* > 0.05) or that there was a difference between men and women (*p* > 0.05 in all cases). There was no significant difference between males and females for running addiction scale (22.64 and 20.91, respectively), social physique anxiety scale (22.30 and 22.61, respectively) or total exercise dependence scale scores (72.56 and 66.86, respectively).
Gapin et al. [69]	(2011)	USA	Cross-sectional	*n* = 179 regular runners; 88 males and 91 females; 91 obligatory and 88 non-obligatory runners	Disordered eating (Eating Disorder Inventory, Athletic Identity Measurement Scale and Obligatory Exercise Questionnaire)	Comparing disordered eating in obligatory and non-obligatory runners.	Obligated running (exercising to maintain identification with the running role) may be associated with pathological eating and training practices, with obligatory runners scoring significantly higher on all of the Eating Disorder Inventory measures (F_(1,166)_ = 9.75, *p* < 0.002),and the Athletic Identity Measure Scale (F_(8,161)_ = 8.85, *p* < 0.001). Results also indicated that runners in the obligatory group demonstrated greater concern with dieting, preoccupation with weight and pursuit of thinness.
Wadas [70]	(2014)	USA	Cross-sectional	*n* = 68 male, high school cross country runners; mean age 15.9	Disordered eating behaviours (questionnaire consisting of The Exercise Motivation Inventory 2, the Eating Attitudes Test 26 and the ATHLETE questionnaire)	Investigating any relationship between male runners with disordered eating behaviours and eating attitudes	Risk factors associated with eating disorders within high school male cross-country runners were found. Factors that had a significant relationship with disordered eating were weight management (r = 0.31, *p* = 0.011), drive for thinness and performance (r = 0.36, *p* < 0.05), and feelings about performance/performance perfectionism (r = 0.26, *p* < 0.05). No significant relationships were found between disordered eating behaviours and personal body feelings (r = 0.19, *p* = 0.109), feelings about eating (r = 0.18, *p* = 0.137), and feelings about being an athlete (r = 0.12, *p* = 0.345); 4.41% (*n* = 3) of participants scored 20 or higher on the EAT-26, indicating being at risk for disordered eating and displaying symptoms. An additional 13.2% (*n* = 9) met the cutoff score of 14 for disordered eating behaviours.
Samson et al. [71]	(2015)	USA	Cross-sectional	*n* = 308 marathon runners; 177 males and 191 females; mean age 41	Self-esteem and psychological coping (Motivation for Marathons Scale, The Perceived control questionnaire and Sport Mental Toughness Questionnaire)	Investigating the relationship between self-esteem and psychological coping with marathon running	Self-esteem was positively associated with perceived control (r = 0.40) (x^2^_7_ = 47.08, *p* < 0.001, CFI = 0.85 and RMSEA = 0.14) but negatively associated with mental toughness. There was also a positive relationship between perceived control and psychological coping (r = 0.42) (x^2^_8_ = 45.65, *p* < 0.001, CFI = 0.85 and RMSEA = 0.12). Results of the Motivations of Marathoners Scales suggested than females were more likely to run to improve psychological coping (4.8 and 4.42, respectively) and self-esteem (5.22 and 4.62, respectively) than men.
Lucidi et al. [72]	(2016)	Italy	Cross-sectional prospective field study	*n* = 669 runners training for a marathon; 569 males and 100 females; mean age 42.07	Stress (Perceived Stress Scale, the Passion Scale and The Italian version of the Locomotion and Assessment Scales)	Investigating the relationship between running and stress in runners training for a marathon	Results suggest that running does not directly impact stress (β = −0.01; *p* = 0.75); however, running increases harmonious passion (β = 0.37; *p* < 0.001), which in turn reduced athletes’ experience of stress. The indirect effect of running on anticipatory stress perception through harmonious passion was statistically significant (αβ = −0.10; 95% confidence interval: from −0.15 to −0.05). Similarly, the indirect effect of assessment on stress through obsessive passion was statistically significant (αβ = 0.12; 95% confidence interval: from 0.07 to 0.17). Results also indicated a significant direct effect of assessment on the athletes’ experience of stress (β = 0.22; *p* < 0.001).
Batmyagmar et al. [73]	(2019)	Austria	Prospective longitudinal study	*n* = 99 participants; *n* = 50 elderly marathon runners, mean age of 66, with 46 men and 4 women vs. *n* = 49 non-exercising controls, mean age of 66, with 44 men and 5 women	Self-reported health and well-being and quality of life (Short Form Health Survey-36)	Comparing self-reported health and well-being, and quality of life over 4 years in elderly marathon runners to non-exercising controls	Findings suggested that extensive high-intensity endurance exercise is linked with improved subjective health and well-being in elderly persons, with athletes evaluating their health as better than non-athletes in the following categories: general health perception (F = 14.21, *p* < 0.001), vitality (F = 13.37, *p* < 0.001), social functioning (F = 11.30, *p* < 0.001), emotional role functioning (F = 1.42, *p* < 0.002) and mental health (F = 6.07, *p* < 0.0016).
Cleland et al. [74]	(2019)	Australia	Cross-sectional	372 participants of “Parkrun” events; mean age 43.8	Enjoyment, self-efficacy and factors of participation in Parkrun event (author-created questionnaires).	Investigating enjoyment, self-efficacy and factors of participation in Parkrun event participants	Overall results suggested that perceived social benefits (*B* coefficient = 0.43) and self-efficacy for Parkrun (*B* coefficient = 0.18) were positively associated with Parkrun participation. Perceived benefits of Parkrun including enjoyment and social factors (*B* = 0.70) were positively associated with participation, as was overall enjoyment (*B* = 0.30), self-efficacy for Parkrun (*B* = 0.46), social support for Parkrun from family (absolute: *B* = 0.05) and social support from friends (*B* = 0.04) related to Parkrun.
Lukacs et al. [75]	(2019)	Hungary	Cross-sectional	*n* = 257 amateur runners with at least 2 years of running experience; 131 males and 126 females; mean age 40.49	Exercise addiction and psychological features (Exercise Dependence Scale; a Cantril ladder for Overall life satisfaction; SCOFF eating disorder questionnaire; the UCLA 3-item Loneliness Scale; Body Image Subscale from Body Investment scale; and the Depression, Anxiety and Stress Scale-21).	Investigating the prevalence of exercise addiction and psychological features in amateur runners, including perceived health, life satisfaction, loneliness, stress, anxiety, depression, body shape and eating disorders	Respondents (137) were characterized as nondependent symptomatic, 97 were nondependent asymptomatic and 23 were at risk of exercise addiction. Results found that five variables significantly predicted the risk of exercise addiction in runners: weekly time spent running, childhood physical activity, lower educational attainment, anxiety and loneliness (ranges of B = 0.47 to 2.06, 95% CI for odds ratio = 1.61 to 7.86, *p* < 0.001 to *p* = 0.023).

**Table 3:** Summary of data extraction from the 23 single-bout studies.

Author	Year	Country	Design	Population	Mental Health Outcome (Measurement)	Study Aim	Main Findings
Nowlis et al. [77]	(1979)	Canada	Pre-post non-controlled study	*n* = 18, experienced joggers; 5 females and 13 males; age range 17 to 55	Mood and anxiety (Mood Adjective Checklist and State Trait Anxiety Inventory)	Impact of a 12.5-mile jog on mood and anxiety	Significant improvement in measures of pleasantness (2.00 to 2.67, *p* < 0.01); a significant decrease in trait anxiety (34.81 to 33.31, *p* < 0.10); a nonsignificant increase in activation; a reduction in state-anxiety; and a reduction of sadness, anxiety, depression and relaxation subscales
Wilson et al. [78]	(1981)	Canada	Pre-post controlled study	*n* = 42; 20 runners, 12 aerobics class exercisers vs. 10 people having lunch; 23 females and 19 males; age range 21 to 28	Anxiety (State-Trait Anxiety Inventory)	Impact of a solo indoor track run on anxiety	Significant decrease in anxiety post-activity (F_(1,39)_ = 15.63, *p* < 0.003)
Markoff et al. [79]	(1982)	Hawaii	Pre-post non-controlled	*n* = 15, all had run at least 1 marathon; 11 males and 4 females; aged 23–45	Mood (Profile of Mood States)	Impact of a 1-h run on mood	Significant reduction in anxiety (*t =* 2.72, *p* < 0.01) and a nonsignificant reduction in depression (*t =* 1.80, *n*.s.)
Thaxton et al. [80]	(1982)	USA	Non-randomised controlled trial	*n* = 33, regular runners; 24 males and 9 females; mean age 36; 4 groups: outdoor running test (*n* = 6), pre-test but no running test (*n* = 9), no pre-test but running test (*n* = 11), and no pre-test and no running test (*n* = 7).	Mood (Profile of Mood States)	Impact of 30 min outdoor running on mood	Significant differences in the depression scores between the 30 min outdoor running group and abstaining groups (F_(1,29)_ = 4.8, *p* < 0.05) but no significant differences between anxiety, vigour and fatigue scores
McGowan et al. [81]	(1991)	USA	Non-randomised controlled trial	*n* = 72, college students; 25 joggers vs. 11 karate vs. 26 weight lifters vs. 10 science lecture class members	Mood (Profile of Mood States)	Impact of 75 min of jogging on an outdoor track on mood	Significant decrease in total mood disturbance from pre- (35.68) to post- (24.16) test (t_24_ = 2.84, *p* < 0.009) following 75 min of jogging on a track
Goode et al. [82]	(1993)	USA	Pre-post non-controlled	*n* = 150, regular runners; 104 males, 36 females; mean age 31.7	Mood (Profile of Mood States)	Impact of own training for running on mood	Significant alterations in all but one (vigor) of the POMS scales, with a significant reduction post-run in tension/anxiety (mean change of −3.1, *p* < 0.1), depression (mean change of −1.5, *p* < 0.1), confusion (mean change of −1.1, *p* < 0.1) and anger (mean change of −1.8, *p* < 0.1) and a significant increase in fatigue post-run (mean change of +1.8, *p* < 0.1).
Morris et al. [83]	(1994)	UK	Pre-post non-controlled	*n* = 165, members of a road runners club; 98 males and 67 females; mean age 34	Mood (author-devised adjective checklist based on POMS)	Impact of a 3 mile “fun-run” on mood	Increase in positive mood (F_(1,163)_ = 68.18, *p* < 0.001), decrease in negative mood (F_(1.163)_ = 47.62, *p* < 0.001) and greater improvements in mood in women than in men that was not significant (*p* > 0.1).
Rudolph et al. [84]	(1996)	USA	Randomised non-controlled trial	*n* = 36, moderately active female university students; *n* = 12 for 10-, 15- and 20-min interventions; mean age 20.6	Self-efficacy (Exercise-Efficacy Scale)	Impact of various timings of treadmill running on self-efficacy (10, 15 and 20 min)	Significant increase in mean scores of self-efficacy in all 3 groups, from pre to postexercise (F_(1, 33)_ = 74.57, *p* < 0.001), and moderate effect sizes in the 15- (ES = 0.36) and 10- (ES = 0.49) minute conditions although the largest effect size (ES) occurred in the 20-min condition (ES = 0.68)
Cox et al. [85]	(2001)	USA	Randomised controlled trial	*n* = 24, physically active male university students; mean age of 28.3	Psychological affect and well-being (Subjective Exercise Experiences Scale)	Impact of 30 min of treadmill jogging at either 50% or 75% predicted VO_2_ max on psychological affect and well-being	Significant improvement in psychological well-being following an acute bout of aerobic exercise (*p* = 0.037, η^2^_p_ = 0.07)
O’Halloran et al. [86]	(2002)	Australia	Pre-post non-controlled	*n* = 50, regular runners; 25 males and 25 females; mean age 26.6	Mood (Profile of Mood States and Beliefs Concerning Mood Improvements Associated with Running Scale)	Impact of a 60-min treadmill run on mood	Significant reductions in anxiety (composed-anxious POMS scale = 25.6 to 29.12, *p* < 0.05, i.e., more composed-less anxious) and depression (elated-depressed POMS scale = 24.56 to 27.10, *p* < 0.01, i.e., more elated-less depressed)
Szabo et al. [87]	(2003)	UK	Pre-post non-controlled time series quasi-experimental	*n* = 39, sports science university students; 22 males and 17 females; aged 20–23	Anxiety, positive well-being and psychological distress (Spielberger State Anxiety Inventory and Exercise induced Feeling Inventory)	Impact of 20 min of track running on anxiety and feelings	Significant reduction in state anxiety (F_(1.5, 58.3)_ = 5.32, *p* < 0.01) and a positive effect on psychological distress and positive well-being
O’Halloran et al. [88]	(2004)	Australia	Randomised controlled trial	*n* = 160 regular runners; 80 did run vs. 80 no running; 80 males and 80 females; aged 18–40	Mood (Profile of Mood States and Beliefs Concerning Mood Improvements Associated with Running Scale).	Impact of a 60-min treadmill run on mood	Improvements in composure, energy, elation and mental clarity during the run; in the energetic-tired subscale, evident improvements at 25 min (F_(1,156)_ = 10.09, *p* = 0.002); in the rest, non-evident mood improvements until 40 min of running; and more composure (less anxious) (F_(1,156)_ = 9.47, *p* = 0.002) and more clear headedness (less confused) (F_(1,156)_ = 5.57, *p* = 0.02) in runners
Robbins et al. [89]	(2004)	USA	Pre-post non-controlled	*n* = 168, inactive children and adolescents; 86 males and 82 females; mean age 12.6	Self-esteem using the Walking Efficacy Scale	Impact of a 20-min treadmill run on self-efficacy in children and adolescents	Significant increase in children and adolescents’ self-efficacy postexercise (F_(1, 158)_ = 84.31, *p* < 0.001) but significantly lower pre-activity self-efficacy in African American girls reported than the other three race-gender groups (F_(3,164)_ = 5.55, *p* < 0.01)
Pretty et al. [90]	(2005)	UK	Randomised controlled trial	*n* = 100; 45 males and 55 females; mean age 24.6	Mood and self-esteem (Profile of Mood States and Rosenberg Self-Esteem Questionnaire)	Impact of a 20-min treadmill run with rural vs. urban stimuli on mood and self-esteem	Significant increase in self-esteem (from 19.4 to 18.1 on the Rosenberg Self-Esteem Questionnaire, *p* < 0.001), with rural and urban pleasant stimuli producing a significantly greater positive effect on self-esteem than exercise alone, while both rural and urban unpleasant scenes reduced the positive effects of exercise on self-esteem
Hoffman et al. [91]	(2008)	USA	Pre-post pre-experimental study	*n* = 32; 16 regular exercisers and 16 non-exercisers; 8 males and 8 females in each group	Mood (Profile of Mood States)	Impact of a 30-min treadmill run on mood	Decreased total mood disturbance in a 30-min treadmill run in both regular exercisers (−16 points, 95% CI = 7–24) and non-exercisers (−9 points, 95% CI = 1–18) but almost double the effect in exercisers.
Kwan et al. [92]	(2010)	USA	Pre-post non-controlled	*n* = 129; 62 males and 67 females; mean age 22	General affective response (Physical Activity Affect scale)	Impact of a 30-min treadmill run on general affective response	Positive effect of the run on general affective response during exercise (b = 0.52, SE = 0.09, *p* < 0.0001) and 15 min postexercise (b = 0.73, CI_.95_ = 0.56, 0.89, t_(126)_ = 8.63, *p* < 0.0001).
Weinstein et al. [93]	(2010)	USA	Pre-post controlled study	*n* = 30; 15 males and 15 females; 2 with minor depressive disorder, 12 with major depressive disorder and 16 as controls; mean age 39.8	Mood and depression (Becks depression Inventory scale and Profile of Mood States)	Impact of 25 min of increasing graded treadmill running on mood and depression	Not only improvements in depressed mood immediately following exercise (*p* = 0.02) of 25 min of increasing graded treadmill running but also increased depressed mood at 30 min postexercise (F_(1,27)_ = 3.98; *p* = 0.05; ηp^2^ = 0.13) and significant relation between the severity of depression and increases in depressed mood (r = 0.60, *p* = 0.001) at 30 min postexercise
Anderson et al. [94]	(2011)	UK	Randomised controlled trial 2 × 2 mixed design	*n* = 40, from various sports clubs; aged 18–25	Mood (“Incredibly Short Profile of Mood States”)	Impact of a light 10-min outdoor jog on mood	Significant mood enhancement even with a light 10=min jog on a grass playing field (F_(1,38)_ = 24.18, *p* < 0.001, n^2^_p_ = 0.39) compared with a 10-min cognitive task
Kane et al. [95]	(2013)	USA	Pre-post non-controlled	*n* = 34 school children; 16 males and 18 females; aged 11–14	Self-efficacy (Self-efficacy questionnaire adapted for children)	Impact of the running PACER challenge (20 m sprints with increasing pace inside a gymnasium) on self-efficacy in children	Decrease in self-efficacy following participation in the run (from 2.7 to 2.3 following exercise, t = 4.6, *p* < 0.001, large effect size of d = 0.79) but positive correlation between PACER laps and pre- and post-measures of exercise self-efficacy
Szabo et al. [96]	(2013)	Hungary	Pre-post non-controlled	*n* = 50 recreational runners; 37 males and 13 females mean age 29.02	States of affect using the Exercise Induced Feeling Inventory	Impact of a 5 km self-paced run along a public running path on states of affect	Significant positive changes in all 4 measures of states of affect following a 5-km self-paced run: revitalisation (F_(1,48)_ = 145.93, *p* < 0.001, partial n^2^ = 0.75, ES = 2.0), positive engagement (F_(1,48)_ = 97.11, *p* < 0.001, partial n^2^ = 0.67, ES = 1.6), tranquillity (F_(1,48)_ = 85.02, *p* < 0.001, partial n^2^ = 0.64, ES = 1.5) and exhaustion (F_(1,48)_ = 32.25, *p* < 0.001, partial n^2^ = 0.40, ES = 1.0)
McDowell et al. [97]	(2016)	Ireland	Randomised controlled trial	*n* = 53; 27 males and 26 females; mean age of 21.2	Mood and anxiety (Profile and Mood States and State-Trait Anxiety Inventory)	Impact of a 30-min treadmill run on mood and anxiety	Significantly improved state anxiety (F_1,92_ = 12.52, *p* < 0.001), feelings of depression (F_1,86_ = 5.05, *p* < 0.027) and total mood disturbance (F = 36.91, *p* < 0.001) compared to 30 min of seated quiet rest
Rogerson et al. [98]	(2016)	UK	Pre-post non-controlled mixed between-within	*n* = 331 Parkrun attendees; 180 males and 151 females; mean age 40.8	Psychological well-being (Questionnaire containing parts of the Profile of Mood States, Rosenberg Self-esteem scale and Perceived Stress Scale)	Impact of a 5-km park run on psychological well-being	Significant (*p* < 0.001) improvements post-run for self-esteem (7.7% improvement; F_(1, 324)_ = 100.58, η^2^ = 0.24), stress (18.4% improvement; F_(1, 315)_ = 50.78, η^2^ *_p_* = 0.139) and total mood disturbance (14.2% improvement; F_(1, 278)_ = 22.15, η^2^_p_ = 0.07)
Edwards et al. [99]	(2017)	USA	Randomised controlled trial	*n* = 27; 8 joggers vs. 9 walkers vs. 10 stretchers; aged 18–35	Stress and anxiety (Exercise-Induced Feeling Inventory and Affective Circumplex Scale, and the Strait-Trait Anxiety Inventory)	Impact of a 15-min treadmill jog on stress and anxiety	Emotionally protective effect from a 15-min bout of treadmill jogging (*n* = 8) compared to an equivalent amount of time walking (*n* = 9) or stretching (*n* = 10) after exposure to a film clip intended to elicit a negative emotional response, with reduced anxiousness from baseline to post-jog (28.8 to 13.1, *p* = 0.06) on the State-Trait Anxiety Inventory, and increased anger score from baseline to post-film clip in the stretching group (1.2 to 26.0, *p* = 0.048) unlike the walking (*p* = 0.11) and jogging (11.3 to 9.4, *p* = 0.19) groups

**Table 4:** Summary of data extraction from the 9 double-bout studies.

Author	Year	Country	Design	Population	Mental health Outcome (Measurement)	Study Aim	Main Findings
Krotee [108]	(1980)	USA	Pre-post pre-experimental non-controlled	*n* = 78, children aged 7–12.	Anxiety (State-Trait Inventory for Children)	Impact of 50-metre group vs. solo run on anxiety	Children’s anxiety levels increased nonsignificantly following a run in either an individual (30.54 to 32.72, *n*.s.) or a group setting (30.67 to 31.83, *n*.s.).
Wildmann et al. [100]	(1986)	Germany	Pre-post non-controlled	*n* = 21, male long-distance runners; mean age of 29.8	Feelings of pleasantness and changes of mood (Eigenschaftsworterliste scale adjective checklist)	Impact of 2 identical 10-km runs (1 week apart) on feelings of pleasantness and change of mood	Higher scores of good mood and feelings of pleasantness were found following the runs (mean increase of the two runs for all subjects was 2.79 from a total of 19 items, but the increase did not reach significance).
O’Connor et al. [101]	(1991)	USA	Pre-post non-controlled	*n* = 17, members of local running clubs; 10 males and 7 females; mean age 25	Anxiety and body awareness (State-Trait Anxiety Inventory and Body Awareness Scale)	Impact of a 5-mile outdoor group vs. solo run on anxiety	Anxiety levels were reduced following both a group (mean baseline = 34.0 vs. pre-exercise = 42.5 vs. postexercise = 27.5, *p* < 0.05) and solo run (mean baseline = 34.0 vs. pre-exercise = 40.0 vs. postexercise = 30.0, *p* < 0.05).
Nabetani et al. [102]	(2001)	Japan	Pre-post non-controlled	*n* = 15, healthy, moderately active male graduate students	Mood (Mood Checklist Short-form 1)	Impact of a 10-min vs. a 15-min treadmill run on mood	Following the 10-min trial, anxiety significantly decreased (ES = 0.61, *p* < 0.01), whilst there was no significant difference in pleasantness (ES = 0.86) and relaxation (ES = 0.33). Following the 15-min trial, anxiety (ES = 0.51) and pleasantness (ES = 0.62) significantly decreased (*p* < 0.01), but relaxation (ES = 0.07) had no significant pre-post difference.
Bodin et al. [103]	(2003)	Sweden	Pre-post non-controlled within-subjects	*n* = 12, regular runners. 6 male and 6 female. Mean age of 39.7.	Emotional restoration ie. depression/anxiety (Exercise-Induced Feeling Inventory and the Negative Mood Scale).	Impact of 1 h park vs. urban run on depression and anxiety.	Runners preferred the park to the urban environment and perceived it as more psychologically restorative; there was no statistical difference in results for park vs. urban settings, with running in both settings causing a significant decline in anxiety/depression (F_(1,10)_ = 16.2, *p* < 0.002, r = 0.78, effect size = 0.30).
Butryn et al. [104]	(2003)	USA	Pre-post non-controlled within-subjects	*n* = 30, non-elite female distance runners; mean age 31	Mood, feeling states and cognition states (Profile of Mood States, Exercise-Induced Feeling Inventory and Thoughts During Running Scale)	Impact of a 4-mile park vs. urban run on mood	Total mood disturbance scores decreased by 8.97 (*p* < 0.001), with a similar effect following the urban run: total mood disturbance scores decreased by 9.13 (*p* < 0.001).
Kerr et al. [105]	(2006)	Japan	Pre-post non-controlled	*n* = 22, recreational runners; mean age 22.7	Stress and emotions (Tension and Effort Stress Inventory)	Impact of indoor vs. outdoor 5-km run on stress and emotions	Significant pre-post effects irrespective of running condition were found, with an increase in relaxation (F_(1, 21)_ = 5.60, *p* < 0.05) and excitement (F_(1, 21)_ = 24.65, *p* < 0.001) and a decrease in anxiety (F_(1, 21)_ = 9.90, *p* < 0.01).
Rose et al. [106]	(2012)	New Zealand	Pre-post controlled	*n* = 32, all females; 17 sedentary and 15 active; mean age 45	Self-efficacy (Self-Efficacy for Exercise Scale)	Impact of self-paced vs. prescribed pace 30-min treadmill run on self-efficacy	Higher self-efficacy was observed before the prescribed paced run compared to the self-paced run (F_1,28_ = 5.81; *p* < 0.023; n^2^ = 0.17).
Reed et al. [107]	(2013)	UK	Pre-post non-controlled	*n* = 75, children aged 11 and 12	Self-esteem (Rosenberg Self Esteem Scale)	Impact of rural vs. urban 1.5-mile run on self-esteem	Significant increase in self-esteem (F_(1,74)_ = 12.2, *p* < 0.001) was found, but no significant difference between the urban or green exercise condition (F_(1,74)_ = 0.13, *p* = 0.72) or any significant difference between boys and girls were found.

**Table 5:** Summary of data extraction from the 3 triple-bout studies.

Author	Year	Country	Design	Population	Mental Health Outcome (Measurement)	Study Aim	Main Findings
Harte et al. [109]	(1995)	Australia	Pre-post non-randomised controlled-repeated measure design	*n* = 10, male amateur triathletes or marathon runners with a mean age of 27.1	Mood (Profile of Mood States)	Impact of a 12-km outdoor run vs. indoor treadmill run with external stimuli vs. an indoor treadmill run with internal stimuli on mood	While the two indoor runs had a positive effect on mood, outdoor running had an even greater benefit to mood with subjects less anxious (F_(3,35)_ = 14.12, *p* < 0.005), less depressed (F_(3,35)_ = 4.16, *p* < 0.01), less hostile (F_(3,35)_ = 13.13, *p* < 0.005), less fatigued (F_(3,35)_ = 15.09, *p* < 0.005) and more invigorated F_(3,35)_ = 13.01, *p* < 0.005).
Berger, Owen + Motl [110]	(1998)	USA	Pre-post non- controlled study	Study 1: *n* = 71 college students (32 males and 39 females) with a mean age of 21.39; study 2: *n* = 68 college students (28 males and 40 females) with a mean age of 22.22	Mood (Profile of Mood States)	Impact of three 15-min runs of varying intensities (50, 65 or 80% age-adjusted HR max) on mood	Significant overall mood benefits postexercise (F_(6.57)_ = 6.43, *p* < 0.0001) for all intensities but no significant differences between intensities were found.
Markowitz et al. [111]	(2010)	USA	Pre-post controlled trial	*n* = 28, college-aged students; 14 active vs. 14 sedentary controls; mean age 21	Anxiety (State-Trait Anxiety Inventory)	Impact of three 20-min treadmill runs of varying intensities (5% below, 5% above and directly at lactate threshold) on anxiety	This was the only triple-bout study with a sedentary control condition. State anxiety improved postexercise at 5% below (effect size = −0.38, *p* < 0.001) and after exercise at the lactate threshold (effect size = −0.20, *p* < 0.001), but anxiety increased at 5% above the lactate threshold (effect size = +0.13, *p* = 0.0030).

**Table 6:** Summary of data extraction from the 34 longer-term intervention studies.

Author	Year	Country	Design	Population	Mental Health Outcome (Measurement)	Study Aim	Main Findings
Lion [112]	(1978)	USA	Randomised controlled trial	*n* = 6, chronic psychiatric patients; 2 males and 4 females; 3 had the running intervention and 3 were controls; middle aged	Anxiety and body image (State-Trait Anxiety Inventory and Rorschach Inkblot Test for body-boundary image)	Impact of running a mile 3 times per week for 2 months on anxiety and body image in chronic psychiatric patients	Post-test anxiety was significantly reduced in the jogging group vs. control group (t = 3.2, df = 4, *p* < 0.05). Joggers showed an average drop of 9 points on the STAI (39.3 to 30.3) from pre- to post-test, while controls showed an average rise of 4 points (32.6 to 36.6). No statistical difference was found between groups for post-test body image scores.
Blue [113]	(1979)	USA	Pre-post non-controlled	*n* = 2 former inpatients of a psychiatric hospital; 1 male aged 37 and 1 female aged 32	Depression (Zung depression scale)	Impact of 3 runs per week for 9 weeks on depression	Following running intervention, both patients’ depression scores reduced from “moderately depressed” to “mildly depressed” (decrease of 18 and 15 points on the Zung Depression Scale).
Young [114]	(1979)	USA	Pre-post non-controlled	*n* = 32 adults; 4 groups: young males (*n* = 8, mean age 30.13), middle-aged males (*n* = 8, mean age 53.0), young females (*n* = 8, mean age 28.25) and middle aged females (*n* = 8, mean age 50.25)	Anxiety and depression using the Multiple Affect Adjective Checklist	Impact of a 10-week walking/jogging programme consisting of 1 h 3× per week on anxiety and depression	Significant reductions in pre- to post-test anxiety (F_(1,28)_ = 6.01, *p* < 0.05) were found. Results for anxiety and depression both showed significant age differences in favour of older subjects ((F_(1,28)_ = 5.37, *p* < 0.05) and (F_(1,28)_ = 5.21, *p* < 0.05), respectively). However, there was no significant improvement with subject depression scores.
Blumenthal et al. [115]	(1982)	USA	Non-randomised controlled cohort	*n* = 16 healthy adults; 5 males and 11 females; mean age 45.1	Anxiety and mood (Profile of Mood States and the State-Trait Anxiety Inventory)	Impact of 3 times weekly walking-jogging programme for 10 weeks vs. 10 weeks of sedentary controls on anxiety and mood	The exercise group exhibited less tension (F_(1,30)_ = 4.49, *p* < 0.04), depression (F_(1,15)_ = 4.82, *p* < 0.04), fatigue (F_(1,30)_ = 3.88, *p* < 0.05) and confusion (F_(1,15)_ = 4.40, *p* < 0.05) but more vigour (F_(1,15)_ = 3.28, *p* < 0.09) than sedentary controls. No change was observed for either group on the POMS anger subscale. After the 10-week programme, exercisers also exhibited less state anxiety (F_(1,26)_ = 4.15, *p* < 0.05) and less trait anxiety (F_(1,26)_ = 6.05, *p* < 0.02).
Trujillo [116]	(1983)	USA	Randomised controlled trial	*n* = 35 female college students; 13 weight trainers, 12 runners and 10 controls	Self-esteem (Tennessee Self-concept Scale and the Bem Sex Role Inventory)	Impact of a 16-week running programme vs. weight training vs. a control on self-esteem	Both the running and weight training groups showed a significant increase in self-esteem from pre- to post-programme (t_(11)_ = 2.11, *p* < 0.05), while the control group showed a nonsignificant loss in self-esteem (t_(9)_ = 0.55, *p* > 0.05).
Tuckman et al. [117]	(1986)	USA	Randomised non-controlled trial	*n* = 154 children; aged 9–11	Psychological affects such as creativity, perceptual function, behaviour and self-concept (Alternate Uses Test, Bender–Gestalt Test, Devereaux Elementary School Behaviour Rating Scale and Piers–Harris Children’s Self-Concept Scale)	Impact of three 30-min runs per week on an outdoor running track for 12 weeks on psychological affects in children (creativity, perceptual function, behaviour and self-concept, compared to 12 weeks of the school’s regular physical education	Running significantly improved creativity of school children compared to regular physical education participants (F ratio = 17.00, *p* < 0.001) but had no significant effect on classroom behaviour, perceptual functioning or self-concept.
Doyne et al. [118]	(1987)	USA	Randomised controlled trial	*n* = 40 women; all with a diagnosis of minor or major depression; mean age of 28.52	Depression (Beck’s Depression Inventory, Hamilton Rating Scale for Depression and Depression Adjective Checklists)	Impact of 3 runs per week on an indoor track for 8 weeks on depression in women diagnosed with depression, compared to 8 weeks of weight lifting vs. control	Running statistically and clinically significantly decreased depression scores (F_(4,138)_ = 14.98, *p* < 0.01) relative to the wait-list control group, with improvements reasonably well maintained at 1 year follow-up.
Fremont et al. [119]	(1987)	USA	Randomised non-controlled trial	*n* = 49; 13 males and 36 females; aged 19–62	Depression, anxiety and mood state (Beck’s Depression inventory, State-Trait Anxiety Inventory and The Profile of Mood States)	Impact of 3 runs per week for 10 weeks on depression, anxiety and mood vs. 10 weeks of counselling vs. 10 weeks of a combination of running and counselling	Depression, trait anxiety and state anxiety all decreased significantly ((F_(4,184)_ = 50.3, *p* < 0.0001), (F_(1, 46)_ = 27.1, *p* < 0.0001), (F_(1,46)_ = 21.9, *p* < 0.0001), respectively), while mood improved significantly over the 10 weeks (F_(18,378)_ = 4.5, *p* < 0.001).
Hannaford et al. [120]	(1988)	USA	Randomised controlled trial	*n* = 27 male psychiatric patients with major psychiatric disorders; age range 25–60; 9 runners, 9 in corrective therapy for 8 weeks and 9 waiting list controls	Depression and anxiety (Zung Self Rating Depression Scale and State Trait Anxiety Index)	Impact of three 30-min runs per week for 8 weeks on depression and anxiety in psychiatric patients with major psychiatric disorders	Significant reductions were observed in depression scores (F_(2,23)_ = 3.61, *p* = 0.043) compared to the waiting list controls, and nonsignificant reductions were observed in anxiety scores (F_(2,23)_ = 1.085, *p* = 0.354) compared to the waiting list control group.
Long et al. [121]	(1988)	Canada	Randomised non-controlled trial	*n* = 39 chronically stressed, sedentary working women; mean age 40; 18 joggers vs. 21 relaxation intervention	Stress, anxiety and self-efficacy (Trait Anxiety Inventory, Sherer et al.’s Inventory for Self-Efficacy and a modified version of the Ways of Coping Checklist)	Impact of an 8-week running programme consisting of a weekly group session plus twice weekly solo jogs on stress, anxiety and self-efficacy	Runners had significantly less anxiety and greater self-efficacy than baseline; 24% of subjects reached clinically significant improvements at the end of treatment, and 36% reached clinically significant improvements at 14-month follow-up. The jogging group exhibited higher self-efficacy, and the time effect for the pre to the post/follow-up average was significant for both self-efficacy and trait anxiety (F_(2, 36)_ = 15.38, *p* < 0.001), while total coping scores did not change (F_(2, 35)_ = 2.88, *p* < 0.07) from pre to post/follow-up.
Simons et al. [122]	(1988)	USA	Non-randomised controlled trial	*n* = 128; 53 experimental subjects (24 male, 30 female, mean age 44.9); 75 control subjects (28 male, 47 female, mean age of 42.0)	Mood (Profile of Mood States, Nowicki–Strickland Internal–External Control Scale for Adults and Marlowe–Crowne Social Desirability Scale)	Impact of two 30 min walk/runs per week for 8 weeks on mood, compared to a weekly 30-min fitness lecture for 8 weeks	Significant improvement in mood pre- to post-test intervention compared to non-treatment controls (F_(1,126)_ = 4.46, *p* < 0.05) as well as significant improvement in pre- to follow-up mood change scores (F_(1,98)_ = 7.63, *p* < 0.01) were observed.
Moses et al. [123]	(1989)	UK	Randomised controlled trial	*n* = 75 sedentary adult volunteers; mean age 38.8; four 10-week conditions: high-intensity aerobic walk-jog programme (*n* = 18); moderate intensity walk-jog programme (*n* = 19); attention-placebo including strength, mobility and flexibility exercises (*n* = 18); or waiting list control (*n* = 20).	Mood and mental well-being (Profile of Mood States and the Hospital Anxiety and Depression Scale)	Impact of varying intensity 10 week walk-jog programmes on mood and mental well-being	Significant reductions in tension/anxiety (F_(3,71)_ = 2.94, *p* < 0.05) were reported only by subjects in the moderate exercise condition. Significant differences in the confusion subscale were found over time (F_(1,71)_ = 3.70, *p* < 0.06), with greater decreases in the moderate exercise group than in the high exercise, attention-placebo or waiting list conditions. No significant effects were found on the perceived coping scales, but there was significant improvement on the physical well-being scale in the exercisers (F_(3,71)_ = 3.82, *p* < 0.01) after 10 weeks, while the waiting list group ratings decreased. At follow-up, only subjects in the moderate exercise condition reported decreased ratings of depression/dejection (F_(2,55)_ = 3.00, *p* < 0.06) and positive changes that approached significance for the perceived coping assets scale (F_(2,55)_ = 2.56, *p* < 0.08), but this was not the case for the high exercise or attention-placebo conditions.
Ossip-Klein et al. [124]	(1989)	USA	Randomised controlled trial	*n* = 32 clinically depressed women; mean age 28.52	Self-concept (Beck Self-Concept Test)	Impact of running on an indoor track 4 times per week for 8 weeks on self-concept in clinically depressed women compared to weight lifting 4 times weekly vs. a delayed treatment (assessment only) control	Self-concept significantly improved in the clinically depressed women compared to controls (F_(3,99)_ = 7.62, *p* < 0.0001). Self-concept scores were also significantly higher in those in the running condition compared to the wait-list condition at post-treatment (F_(2, 33)_ = 4.69, *p* < 0.05), with improvements also reasonably well-maintained over time.
Morris et al. [125]	(1990)	UK	Pre-post study with randomised comparison	*n* = 30 male regular runners; mean age 37; 20 participants stopped running for 2 weeks vs. 20 continued running as normal	Anxiety and depression (General Health Questionnaire and short forms of the Zung Anxiety and Zung Depression scales)	Impact of stopping running for 2 weeks on anxiety and depression	Somatic symptoms, anxiety/insomnia and social dysfunction, symptoms of depression (*p* < 0.05), were all significantly greater in deprived than in continuing runners, and Zung depression (F_(1,37)_ = 22.64, *p* < 0.001) and anxiety (F_(1,37)_ = 11.51, *p* < 0.01) scores were significantly higher after the two weeks. Significantly more deprived than non-deprived subjects exceeded the suggested cutoff score for a psychiatric case after both weeks of deprivation (x^2^ = 5.38 and 4.51, respectively, df = 1, *p* < 0.05), but there was no statistical difference between groups once the deprived group resumed running.
Friedman et al. [126]	(1991)	USA	Randomised controlled trial	*n* = 387 students; 177 males and 188 females; mean age 20; 84 joggers, 96 relaxation, 100 group interaction and 107 lecture-control	Stress and mood (Profile of Mood States and Bem Sex Role Inventory)	Impact of 12 weeks of jogging on stress and mood	High masculinity male and female joggers reported significantly more mood improvement than those with low masculinity (*p* < 0.004). All women joggers reported significant reductions in depression after jogging, but those with high psychological masculinity experienced significantly greater reductions than low masculinity joggers (*p* < 0.04). Femininity had a significant effect on combined POMS scores (F_(6,297)_ = 2.79, *p* < 0.02), with higher psychological femininity associated with higher tension, depression and fatigue and with lower vigour and confusion scores compared to those low in femininity. There were significant pre-post session × technique interactions for high and low masculinity women (F_(18,843.36)_ = 2.47, *p* < 0.0007; F_(18,843.36)_ = 2.49, *p* < 0.0006, respectively). Short-term improvements in POMS scores depended upon masculinity for women joggers and participants in group interaction.
Williams et al. [127]	(1991)	USA	Pre-post non-controlled within subject design	*n* = 10 moderately trained male runners; mean age 25.6	Mood (Profile of Mood States)	Impact of 4 weeks of treadmill running 5 times per week at set paces reflecting 50, 60 and 70% VO_2_ max on mood	Regarding within-subject data, an increase in mean VO_2_ was associated with a significant increase in total mood disturbance (r = 0.88, *p* < 0.01), while running at a pace with more economical values was associated with more positive mental health profiles. However, when considered as a group, there was no relationship between running efficiency in moderately trained male runners and total mood disturbance.
Kerr et al. [128]	(1993)	Holland	Pre-post non-controlled	*n* = 32 regular exercising university students (18 males and 14 females) aged 18–22	Mood (Stress-Arousal Checklist and Telic State Measure)	Impact of a weekly 40-min fixed distance run (5 km for females, 6.6 km for males) through a wooded area for 7 weeks on mood	In both males and females, there were significant increases from pre- to post-running intervention in telic state measure felt arousal scores (F_(1,16)_ = 52.37, *p* = 0.0001 and F_(1,12)_ = 16.16, *p* = 0.002, respectively), stress-arousal checklist arousal scores (F_(1,16)_ = 15.34, *p* = 0.001 and F_(1,12)_ = 25.19, *p* = 0.0001, respectively) and telic state measure preferred arousal scores (F_(1,16)_ = 4.49, *p* = 0.05 and F_(1,12)_ = 11.82, *p* = 0.005, respectively). In contrast, telic state measure arousal discrepancy scores decreased significantly for males (F_(1,16)_ = 6.74, *p* = 0.02) and females (F_(1,12)_ = 11.86, *p* = 0.005) pre- to post-running.
Long [129]	(1993)	Canada	Randomised controlled trial	*n* = 35; 14 males and 21 females; mean age 35.6; 12 runners, 9 stress inoculation and 14 wait-list control	Anxiety and stress (Cornell Medical Symptom Checklist)	Impact of 3 runs per week for 10 weeks on anxiety and stress	Although the exercise group was more likely to report using exercise to cope with stress, there was no significant differences found between groups on stress or coping classifications. There was also no significant difference in scores of the Cornell Medical Symptom Checklist between the running group and the stress inoculation treatment groups (F < 1; M = 87.4 vs. M = 86.2, respectively).
Berger and Friedman [130]	(1998)	USA	Randomised controlled trial	*n* = 387 undergraduate college students; 117 males and 188 females; mean age 20.0; 84 joggers vs. 96 relaxation response vs. 100 in discussion groups vs. 107 in the control group	Stress and mood (Profile of Mood States)	Impact of three jogs per week for a minimum of 20 min over 12 weeks on stress and mood	Jogging was significantly more effective in reducing stress than the control activity (F_(18,280)_ = 1.79 to 1.85, *p* < 0.03), and joggers reported larger and more numerous reductions in tension, depression and anger than the control group; however, changes in vigour, fatigue and confusion were sporadic. There were no long-term benefits observed.
Berger and Owen [131]	(1998)	USA	Pre-post with comparison	*n* = 91 college students; *n* = 67 in weekly walking/jogging (32 males and 35 females) vs. *n* = 24 in a weekly health science class (9 males and 15 females)	Mood and anxiety (Profile of Mood States and State-Trait Anxiety Inventory)	Impact of twice weekly walking/jogging for 14 weeks on mood and anxiety	No significant interaction between exercise intensity and pre-post mood benefits was observed. Joggers reported significant short-term mood benefits following running regardless of exercise intensities (F_(6,56)_ = 4.87, *p* < 0.0005). Joggers reported significant pre-post exercise changes on all POMS subscales: tension (F = 15.67, *p* < 0.0002), depression (F = 15.64, *p* < 0.0002), anger (F = 12.77, *p* < 0.0007), vigour (F = 22.29, *p* < 0.00005), fatigue (F = 20.14, *p* < 0.00005) and confusion (F = 26.34, *p* < 0.00005).
Szabo et al. [132]	(1998)	UK	Pre-post non-controlled observational cohort study	*n* = 40 members of an amateur running club; 30 males, mean age 40.5, and 10 females, mean age 37	Anxiety and mood, i.e., exhaustion, tranquillity, positive engagement and revitalization (Commitment to running scale, Exercise-induced Feeling Inventory and Spielberger State Anxiety Inventory)	Impact of running vs. non-running days on anxiety and mood over 21 consecutive days	Reported differences (effect sizes ranging from 0.07 to 0.56, all *p* < 0.05) all favour running days over non-running days, concluding that, on running days, runners experienced less anxiety (F_(1,38)_ = 5.22, *p* < 0.03) and better subscales of mood: exhaustion (F_(1,38)_ = 4.34, *p* < 0.04), tranquillity (F_(1,38)_ = 5.56, *p* < 0.02), revitalisation (F_(1,38)_ = 18.32, *p* < 0.001) and positive engagement (F_(1,38)_ = 11.79, *p* < 0.001).
Broman-Fulks et al. [133]	(2004)	USA	Randomised non-controlled trial	*n* = 54 participants with elevated anxiety sensitivity scores; 13 males and 41 females; mean age 21.17; 29 high-intensity aerobic exercisers vs. 25 low-intensity aerobic exercisers	Anxiety sensitivity (Anxiety Sensitivity Index, State-trait Anxiety Inventory and Body Sensations Questionnaire)	Impact of six 20-min treadmill sessions of either high/low-intensity aerobic exercise across 2 weeks on anxiety sensitivity in participants with elevated anxiety sensitivity scores	Six 20-min treadmill sessions of both high-intensity and low-intensity running across 2 weeks reduced anxiety sensitivity (F_(2,56)_ = 42.50, *p* < 0.001, n^2^ = 0.60; F_(2, 48)_ = 13.72, *p* < 0.001, n^2^ = 0.36; respectively). State anxiety also decreased from pre-post high-intensity running (35.10 to 32.03); however, it increased following low-intensity running (42.72 to 42.32), but neither of these effects were significant.
Haffmans et al. [134]	(2006)	Holland	Randomised controlled trial	*n* = 60 psychiatric patients all suffering from a depressive disorder; 19 males and 41 females; mean age 39; 20 runners vs. 21 in physiotherapy training vs. 19 controls	Depression and self-efficacy (Hamilton Rating Scale for Depression, Becks Depression Inventory, Self-Efficacy Scale and Physical Self-Efficacy Scale)	Impact of running therapy for 3 days per week for 12 weeks on depression and self-efficacy in psychiatric patients all suffering from depression	While after 6 weeks of running, self-efficacy was significantly higher (*p* = 0.03), after the full 12 weeks of running, there was no significant difference in depression (26.7 to 25.5, *n*.s.) or self-efficacy (46.6 to 49.1, *n*.s.) scores from baseline.
Thornton et al. [135]	(2008)	USA	Repeated measures design	*n* = 50 runners over age 18	Anxiety (Beck Anxiety Inventory)	The relationship between anxiety and marathon	Marathon training decreased Beck Anxiety Inventory scores (0.9) initially from baseline pre-training levels compared to 2 months prior to marathon day (0.7; 72% had no change from baseline, 22% were less anxious and 6% were more anxious). However, anxiety scores increased as race day approached: at 1 month prior to race day (1.4; 46% had no change from baseline, 19% were less anxious and 35% were more anxious than baseline) and 1 week prior to race (2.6; 22% had no change from baseline, 14% were less anxious and 64% were more anxious than baseline, respectively).
Scholz et al. [136]	(2008)	Switzerland	Pre-post non-controlled non-experimental longitudinal study	*n* = 30 untrained participants; 4 males and 26 females; mean age 41.2	Self-efficacy (4-part author-created measurement)	Impact of a 1-year marathon training programme on self-efficacy	The trend between running and self-efficacy had substantial correlation but was not significant. No statically significant differences was observed in the baseline level, trend or fluctuation of self-efficacy between the participants who successfully completed the marathon and those who did not, but the baseline level of self-efficacy was positively associated with the baseline level in running (correlation analyses = 0.27; *p* < 0.05; 95% CI = 0.00; 0.53) and fluctuation in self-efficacy correlated positively with fluctuation in running (0.39; *p* < 0.05; 95% CI = 0.03; 0.74). As this was a non-experimental longitudinal study, no causal statements can be drawn.
Kalak et al. [137]	(2012)	Switzerland	Randomised controlled trial	*n* = 51 adolescents; 24 males and 27 females; mean age 18.3; 27 runners vs. 24 controls	Mood and stress (A-a daily mood log, a questionnaire assessing positive and negative comping strategies, and Perceived Stress Scale)	Impact of daily 30-min morning runs on weekdays for 3 weeks (i.e., 3 × 5 runs) on stress and mood	Perceived stress did not differ significantly between running and control groups over time (F_(1,49)_ = 1.71, n^2^ = 0.034, *n*.s.), while mood in the morning increased significantly over time in the running group compared with controls (F_(5,245)_ = 16.08, n^2^ = 0.247, *p* < 0.05). However, irrespective of group, mood in the evening improved, and there was no significant difference of mood in the evening between groups.
Inoue et al. [138]	(2013)	USA	Pre-post non-controlled	*n* = 148 homeless people; 134 males and 14 females; mean age 29.9	Self-sufficiency (author-created scale)	Impact of 10 organised runs on self-sufficiency in homeless people	Running involvement had a significant positive correlation with perceived self-sufficiency (r = 0.30, *p* < 0.01). Results suggested that participants gained higher levels of perceived self-sufficiency as they became more involved with running during the program (F = 3.39, *p* < 0.01, Adjusted R2 = 0.08), and increases in running involvement were the sole significant predictor of the outcome (β = 0.29, t = 3.57, *p* < 0.01).
Samson et al. [76]	(2013)	USA	Pre-post non-controlled	*n* = 39 university students who all had running experience; 11 males and 28 females; mean age 20.5	General affect and self-efficacy (Positive and Negative Affect Scale and author-created measurements for self-efficacy)	Impact of a 15-week marathon training program of 3 group training days per week and one run of 8–20 miles on the weekend on general affect and self-efficacy	Self-efficacy significantly increased over the training programme (F_(12,444)_ = 5.81, *p* < 0.01), but there was a significant decrease of positive affect over time (F_(12,444)_ = 8.35, *p* < 0.01) and no significant change was found for negative affect over the programme.
Doose et al. [139]	(2015)	Germany	Randomised controlled trial	*n* = 46 outpatients diagnosed with mild to severe depression; aged 18–65; 30 walker/runner vs. 16 controls	Depression (Hamilton Rating Scale and Beck Depression Inventory)	Impact of group walking/running 3 times per week for 8 weeks on depression	Depression clinically significantly decreased on the Hamilton Rating Scale (Cohen’s d = 1.8; mean change = 8.24; *p* = <0.0001), and while there were reductions, they were without clinical significance (Cohen’s d = 0.50; mean change = 4.66; *p* = 0.09) in the Becks Depression Inventory scores.
Von Haaren et al. [140]	(2015)	Germany	Randomised controlled trial, within subject design	*n* = 61 inactive male university students; mean age 21.4	Stress and mood (a shorten mood scale based on the Multidimensional Mood Questionnaire and a one-item test for perceived control and stress)	Impact of a 20-week running training course on stress and mood during academic examinations, compared to waiting list controls	Significant emotional stress reactivity was observed in both groups during academic assessment episodes; participants in aerobic training showed lower emotional stress reactivity compared with the control participants after the 20-week training programme, with perceived stress of the aerobic group remaining similar during both exam periods (2.27 to 2.24), while it increased further in the control group (2.43 to 2.51).
Kahan et al. [141]	(2018)	USA	Pre-post with comparison	*n* = 11 children; 9 males and 2 females; aged 9 and 10	Self-esteem and self-efficacy (50-item, author-created questionnaire)	Impact of 20 running sessions alternating between game vs. lap running on self-esteem and self-efficacy in children	Means for self-esteem and task-efficacy were 3.63 and 4.16, respectively, on a 5-point scale, while the mean for task-efficacy was 4.16 on a 5-point scale, and high inherent-interest participants (i.e., higher moderate–vigorous physical activity in the running laps condition) had statistically significant higher scores than low inherent-interest participants on recognition (*p* = 0.01), ego orientation (*p* = 0.03) and expectancy beliefs (*p* = 0.03) subscales. There were no direct comparisons of self-esteem and self-efficacy in game vs. lap running.
Keating et al. [142]	(2018)	Canada	Pre-post non-controlled	*n* = 46 participants with complex mood disorders; 11 males and 35 females; 29 youths (mean age 22.1) and 17 adults (mean age 45.2)	Stress, anxiety and depression (Cohen’s Perceived Stress Scale, Becks Depression Inventory, Becks Anxiety Inventory and Short Form Survey)	Impact of 12 weeks of twice weekly running in a group setting that offers social support supervised by clinical professionals on stress, anxiety and depression	Significant decreases in depression (F_(11,201)_ = 4.5, *p* < 0.0001), anxiety (F_(11,186)_ = 4.8, *p* < 0.0001) and stress (F_(11,186)_ = 2.3, *p* = 0.01) from baseline was observed. Following intervention, mean depression scores decreased by 39% in adults from high to low levels and by 27% in youths from moderate to reduced moderate levels. Younger participant age, younger age at onset of illness and higher perceived levels of friendship with other running group members (ps ≤ 0.04) were associated with lower depression, anxiety and stress scores. Higher attendance was linked with decreasing depression and anxiety (ps ≤ 0.01) scores over time.
Nezlek et al. [143]	(2018)	Poland	Pre-post observational cohort study over 3 months with no control	*n* = 244 recreational runners; 127 males and 117 females; mean age 32.5	Psychological well-being, self-esteem, self-efficacy and affect (Rosenberg Self-esteem Scale, Satisfaction with Life Scale, and a circumplex model that distinguishes the valence and arousal of affect)	Impact of 3 months of self-prescribed running on psychological well-being, self-esteem, self-efficacy and affect	Positive within-person relationships between how much people ran each week and self-reports of well-being were observed, with well-being significantly higher during weeks when individuals ran more often and further. Self-efficacy was related to distance run but not to frequency. For the km that people ran each week, significant moderation was found for weekly Satisfaction with Life Scale (γ_11_ = −0.0002, *p* = 0.013), self-esteem (γ_11_ = −0.0002, *p* = 0.015), positive activated affect (γ_11_ = −0.0003, *p* < 0.001), positive deactivated affect (γ_11_ = −0.0008, *p* < 0.01), negative activated affect (γ_11_ = 0.0002, *p* = 0.046) and negative deactivated affect (γ_11_ = 0.0003, *p* = 0.01).
Kruisdijk et al. [144]	(2019)	Holland	Randomised controlled trial	*n* = 48 participants with major depressive disorder; mean age 42.6; 25 runner-walkers vs. 23 controls	Depression (Hamilton Depression Scale)	Impact of 6 months of running-walking for one hour twice a week on depression in subjects with major depressive disorder	No significant difference or effect on depression in favour of the intervention group (Cohen’s d < 0.2, F = 0.13, *p* = 0.73) with only 9 participants (19%) completing the study was found, with low statistical power and lack of follow-up at six and 12 months.

**Table 7:** Summary of key findings within each of the three categories.

Study Type	Number of Studies	Summary of Evidence
**Cross-sectional**	47 studies	Consistent evidence was found for a positive association between mental health and habitual or long-term recreational running compared to non-runners. In contrast, there was evidence that high or extreme levels of running were associated with markers of running ill-health compared to levels of moderate running.
**Acute: single/double/triple bout**	35 studies	Overall, these studies suggest that acute bouts of running can improve mental health and that the type of running can lead to differential effects. Evidence suggests that acute bouts of treadmill, track, outdoor and social running (2.5–20 km and 10–60 min) all result in improved mental health outcomes. There were few differences between high and low intensities. Studies consistently show that any running improves acute/short-term mood markers but that lack of inactive comparisons limits the strength of evidence. Little variation in the demographics of participants and small sample sizes limit generalizability and precision of findings.
**Interventions (2 weeks or more)**	34 studies	Overall, running interventions of 2–20 weeks generally show improved markers of a range of mental health outcomes compared to non-running controls, including mental health outcomes in psychiatric and homeless populations. The risk of longer-term running interventions on adverse mental health outcomes remains unclear.

## Supplementary Materials

The following are available online at https://www.mdpi.com/1660-4601/17/21/8059/s1, Table S1: Narrative description of findings of the 47 cross-sectional studies. Table S2–S4: Narrative description of findings of the 35 studies with an acute bout of running. Table S5: Narrative description of findings of the 34 studies with a longer-term intervention of running.

## Appendix A

**Table A1:** Preferred Reporting Items for Systematic reviews and Meta-Analyses extension for Scoping Reviews (PRISMA-ScR) Checklist [18].

Section	Item	PRISMA-ScR CHECKLIST ITEM	Reported on Page Number
**Title**			
Title	1	Identify the report as a scoping review.	1
**Abstract**			
Structured summary	2	Provide a structured summary that includes (as applicable) background, objectives, eligibility criteria, sources of evidence, charting methods, results and conclusions that relate to the review questions and objectives.	1
**Introduction**			
Rationale	3	Describe the rationale for the review in the context of what is already known. Explain why the review questions/objectives lend themselves to a scoping review approach.	1–2
Objectives	4	Provide an explicit statement of the questions and objectives being addressed with reference to their key elements (e.g., population or participants, concepts and context) or other relevant key elements used to conceptualize the review questions and/or objectives.	1–2
**Methods**			
Protocol and registration	5	Indicate whether a review protocol exists; state if and where it can be accessed (e.g., a Web address), and if available, provide registration information, including the registration number.	2
Eligibility criteria	6	Specify characteristics of the sources of evidence used as eligibility criteria (e.g., years considered, language and publication status), and provide a rationale.	3
Information sources *	7	Describe all information sources in the search (e.g., databases with dates of coverage and contact with authors to identify additional sources) as well as the date that the most recent search was executed.	4
Search	8	Present the full electronic search strategy for at least 1 database, including any limits used, such that it could be repeated.	Appendix B
Selection of sources of evidence †	9	State the process for selecting sources of evidence (i.e., screening and eligibility) included in the scoping review.	4
Data charting process ‡	10	Describe the methods of charting data from the included sources of evidence (e.g., calibrated forms or forms that have been tested by the team before their use and whether data charting was done independently or in duplicate) and any processes for obtaining and confirming data from investigators.	4
Data items	11	List and define all variables for which data were sought and any assumptions and simplifications made.	4
Critical appraisal of individual sources of evidence §	12	If done, provide a rationale for conducting a critical appraisal of included sources of evidence; describe the methods used and how this information was used in any data synthesis (if appropriate).	N/A
Synthesis of results	13	Describe the methods of handling and summarizing the data that were charted.	4
**Results**			
Selection of sources of evidence	14	Give numbers of sources of evidence screened, assessed for eligibility and included in the review, with reasons for exclusions at each stage, ideally using a flow diagram.	4
Characteristics of sources of evidence	15	For each source of evidence, present characteristics for which data were charted and provide the citations.	Table 2, Table 3, Table 4, Table 5 and Table 6
Critical appraisal within sources of evidence	16	If done, present data on critical appraisal of included sources of evidence (see item 12).	N/A
Results of individual sources of evidence	17	For each included source of evidence, present the relevant data that were charted that relate to the review questions and objectives.	Table 2, Table 3, Table 4, Table 5 and Table 6
Synthesis of results	18	Summarize and/or present the charting results as they relate to the review questions and objectives.	5–40
**Discussion**			
Summary of evidence	19	Summarize the main results (including an overview of concepts, themes and types of evidence available), link to the review questions and objectives and consider the relevance to key groups.	40–41
Limitations	20	Discuss the limitations of the scoping review process.	41
Conclusions	21	Provide a general interpretation of the results with respect to the review questions and objectives as well as potential implications and/or next steps.	42
**Funding**			
Funding	22	Describe sources of funding for the included sources of evidence as well as sources of funding for the scoping review. Describe the role of the funders of the scoping review.	43

JBI = Joanna Briggs Institute; PRISMA-ScR = Preferred Reporting Items for Systematic reviews and Meta-Analyses extension for Scoping Reviews. * From where sources of evidence (see second footnote) are compiled, such as bibliographic databases, social media platforms and websites. † A more inclusive/heterogeneous term used to account for the different types of evidence or data sources (e.g., quantitative and/or qualitative research, expert opinion and policy documents) that may be eligible in a scoping review as opposed to only studies. This is not to be confused with *information sources* (see first footnote). ‡ The frameworks by Arksey and O’Malley (1) and Levac and colleagues (2) and the JBI guidance (3,4) refer to the process of data extraction in a scoping review as data charting. § The process of systematically examining research evidence to assess its validity, results and relevance before using it to inform a decision. This term is used for items 12 and 19 instead of “risk of bias” (which is more applicable to systematic reviews of interventions) to include and acknowledge the various sources of evidence that may be used in a scoping review (e.g., quantitative and/or qualitative research, expert opinion and policy document).

## Appendix B

**Table A2:** Details of the search strategy used in all 4 databases.

*Notes for Database*	Ovid (Embase)	Ovid (Medline)	Sport DISCUS (Ebscohost)	ProQuest Social Science Journals
	Ti,ab Searches Title & Abstract	Ti,ab Searches Title & Abstract	AB Searches Abstract	Ab Searches Abstract
Running Search Terms				
Run*, Jog*, Sprint*, Park-run, Orienteer, Orienteering, Marathon, Marathon-running, Treadmill				
Mental Health Search Terms				
Mental Health, Mental illness, Mental state, Emotions, Emotional, Depression, Depressive disorder, depressive therapy, Postnatal depression, Postpartum depression, Seasonal affective disorder, Situational depression, Atypical depression, Persistent depressive disorder, anxiety, loneliness, stress, mood, self-efficacy, sleep, psychological, psychological characteristics, psychology, eating disorder, disordered eating, anorexia, bulimia, exercise, health status disparities, quality of life, motivation, adjustment disorder, sick role. relaxation, lifestyle, exercise therapy, social support				
NOT search Terms				
Rodent, mouse, rat, bovine, pig, animal*, horses, mice, ecology, dermatology, epigenetics, gene*, molecule*, cell*, phenotype, drug*, hormone*, food, nutrient*, glucose, imaging, football, tennis, swimming, heart, troponin, cardiology, lung, respiratory, bone, cesarean, newborn, breast-feeding, HIV, cough, rectal, protocol, procedure, surgery, operation, stroke, sacroiliitis, COPD, asthma, Apnoea, angina, allergy, railway, falling				
Search Syntax	(remember the ‘adj’ function)	(remember the ‘adj’ function)	(remember the ‘adj’ function)	(remember the ‘adj’ function)
	(((“mental-health” or “mental-illness” or “mental-state” or emotions or emotional or depression or “depressive-disorder” or “depressive-therapy” or “postpartum-depression” or “seasonal-affective-disorder” or “situational-depression” or “atypical-depression” or “persistent-depressive-disorder” or anxiety or loneliness or stress or mood or “self-efficacy” or sleep or psychological or “psychological-characteristics” or psychology or “eating-disorder” or “disordered-eating” or anorexia or Bulimia or exercise or “health-status-disparities” or “quality-of-life” or motivation or “adjustment-disorder” or “sick-role” or relaxation or lifestyle or “exercise-therapy” or “social-support”)) AND (run* or Jog* or sprint* or “park-run” or orienteer or orienteering or marathon or “Marathon-running” or treadmill) NOT (dermatology OR epigenetics OR gene* OR drug* OR surgery OR hormone* OR food OR imaging OR animal* OR football OR tennis OR swimming OR rodent OR mouse OR rat OR pig OR bovine OR phenotype or Heart or cardiology OR lung or bone OR caesarean OR HIV OR troponin OR cough OR protocol OR breast-feeding OR cell* OR sacroiliitis OR rectal or procedure OR COPD or respiratory OR nutrient* OR glucose or newborn OR stroke OR asthma OR operation OR horses OR falling OR railway OR molecule* OR apn?ea OR angina OR allergy OR mice OR ecology)).ab,ti.	(((“mental-health” or “mental-illness” or “mental-state” or emotions or emotional or depression or “depressive-disorder” or “depressive-therapy” or “postpartum-depression” or “seasonal-affective-disorder” or “situational-depression” or “atypical-depression” or “persistent-depressive-disorder” or anxiety or loneliness or stress or mood or “self-efficacy” or sleep or psychological or “psychological-characteristics” or psychology or “eating-disorder” or “disordered-eating” or anorexia or Bulimia or exercise or “health-status-disparities” or “quality-of-life” or motivation or “adjustment-disorder” or “sick-role” or relaxation or lifestyle or “exercise-therapy” or “social-support”) and (run* or Jog* or sprint* or “park-run” or orienteer or orienteering or marathon or “Marathon-running” or treadmill)) not (dermatology or epigenetics or gene* or drug* or surgery or hormone* or food or imaging or animal* or football or tennis or swimming or rodent or mouse or rat or pig or bovine or phenotype or Heart or cardiology or lung or bone or caesarean or HIV or troponin or cough or protocol or breast-feeding or cell* or sacroiliitis or rectal or procedure or COPD or respiratory or nutrient* or glucose or newborn or stroke or asthma or operation or horses or falling or railway or molecule* or apn?ea or angina or allergy or mice or ecology)).ab,ti.	(AB(run* OR jog* OR sprint OR “park run” OR orienteer OR orienteering OR marathon OR “marathon-running” OR treadmill) AND AB(“mental health” OR “mental illness” OR “mental state” OR emotions OR emotional OR depression OR “depressive disorder” OR “depressive therapy” OR “postpartum depression” OR “seasonal affective disorder” OR “situational depression” OR “atypical depression” OR “persistent depressive disorder” OR anxiety OR loneliness OR stress OR mood OR “self-efficacy” OR sleep OR psychological OR “psychological characteristics” OR psychology OR “eating disorder” OR “disordered eating” OR anorexia OR bulimia OR exercise OR “health status disparities” OR “quality-of-life” OR motivation OR “adjustment disorder” OR “sick role” OR relaxation OR lifestyle OR “exercise therapy” OR “social-support”)) NOT (dermatology OR epigenetics OR gene* OR drug* OR surgery OR hormone* OR food OR imaging OR animal* OR football OR tennis OR swimming OR rodent OR mouse OR rat OR pig OR bovine OR phenotype or Heart or cardiology OR lung or bone OR caesarean OR HIV OR troponin OR cough OR protocol OR breast-feeding OR cell* OR sacroiliitis OR rectal or procedure OR COPD or respiratory OR nutrient* OR glucose or newborn OR stroke OR asthma OR operation OR horses OR falling OR railway OR molecule* OR apnoea OR angina OR allergy OR mice OR ecology)	(ab((run* OR jog* OR sprint OR “park run” OR orienteer OR orienteering OR marathon OR “marathon-running” OR treadmill)) AND ab((“mental health” OR “mental illness” OR “mental state” OR emotions OR emotional OR depression OR “depressive disorder” OR “depressive therapy” OR “postpartum depression” OR “seasonal affective disorder” OR “situational depression” OR “atypical depression” OR “persistent depressive disorder” OR anxiety OR loneliness OR stress OR mood OR “self-efficacy” OR sleep OR psychological OR “psychological characteristics” OR psychology OR “eating disorder” OR “disordered eating” OR anorexia OR bulimia OR exercise OR “health status disparities” OR “quality-of-life” OR motivation OR “adjustment disorder” OR “sick role” OR relaxation OR lifestyle OR “exercise therapy” OR “social-support”)) NOT ab((dermatology OR epigenetics OR gene* OR drug* OR surgery OR hormone* OR food OR imaging OR animal* OR football OR tennis OR swimming OR rodent OR mouse OR rat OR pig OR bovine OR phenotype OR Heart OR cardiology OR lung OR bone OR caesarean OR HIV OR troponin OR cough OR protocol OR breast-feeding OR cell* OR sacroiliitis OR rectal OR procedure OR COPD OR respiratory OR nutrient* OR glucose OR newborn OR stroke OR asthma OR operation OR horses OR falling OR railway OR molecule* OR apnoea OR angina OR allergy OR mice OR ecology))) AND (stype.exact(“Scholarly Journals”) AND la.exact(“ENG”))
Search complete?	Yes	Yes	Yes	Yes
Search saved?	Yes	Yes	Yes	Yes
Saved under:	Embase RunningMH	Medline RunningMH	Sport Discus Running MH	ProQuest RunningMH
Number of hits:	10,131 Text results (this had a limit of only human studies, as well as a limit for articles and articles in press applied to the search)	10,154 text results (this had a limit of human studies applied to the search)	3461 (this had a limit of English studies only, and academic journal only applied to the search)	5933 (this search was carried out within the sports medicine and education index database and in the social sciences database)
Uploaded to Covidence?	Yes	Yes	Yes	Yes

