# Supplementary material for: A Scoping Review of the Relationship between Running and Mental Health
Source: Int J Environ Res Public Health. 2020 Nov 1;17(21):8059. doi: 10.3390/ijerph17218059 (PMC7663387; doi:10.3390/ijerph17218059)
Supplement: Supplementary file 1 [file ijerph-17-08059-s001.pdf]

Supplementary Material

Supplementary Table S1

Narrative description of findings of the 47 cross-sectional studies.

	Author		Narrative description of findings
1	Wilson (1980)	comparing the mood states of marathon runners, regular joggers and non-exercisers	A Canadian cross sectional study by Wilson et al. (1980) used 30 male participants ranging from age 20-45 from the same socioeconomic area to compare the mood states of marathon runners (n=10), regular joggers (n=10) and non-exercisers (n=10) using the Profile of Mood States as measurement. The marathoners and joggers reported less depression ($F_{(2,28)}$ =7.51, p<0.003), less anger (F=10.11, p<0.001), less confusion (F=12.41,p<0.001) and more vigor (F=103.21,p<0.001) than the non-exercisers. The marathoners also reported less fatigue (F=10.26, p<.001) and less tension (F=7.51, p<0.003) than the non-exercisers. The marathoners and joggers did not significantly differ on reported fatigue and tension, however marathoners had significantly less depression, less anger, less confusion and more vigor than the joggers. Overall results found that the joggers reported better mood states than the non-exercisers, and the marathon runners reported even more positive mood states than the joggers[1].
2	Joesting (1981)	investigating the relationship between running and depression	An American controlled cross sectional questionnaire by Joesting (1981) used 100 runners (21 women, mean age 16.53) and 79 men (mean age 18.36) to investigate the relationship between running and depression using the Depression Adjective Checklist as measurement. The only significant sex difference between male and female runners was their age (t=2.85, p<.01). Results found that using the t test, runners of both sexes were significantly (p< .01) less depressed than Lubin's data for non-psychiatric sample of patients. Female runners mean score on the Depression Adjective Check List was 4.33, while the normative non-psychiatric patient normative sample mean was 7.32. Male runners mean depression score was 4.59 while the normative non-psychiatric male sample mean was 8.02. Overall results suggest that running decreased depression measurements in both males and females[2].
3	Jorgenson (1981)	investigating the relationship between emotional wellbeing and running	An American study by Jorgenson et al. (1981) used 454 regular runners (390 males and 64 females) of whom 9.9% were under 20, 25% were age 21-29, 37% were age 30-39, 23% were 40 and over, 4.8 did not respond about age. The study used a structured questionnaire consisting of 55 items designed by the author to investigate the relationship between emotional wellbeing and running. The majority of runners (92.3%, n=419) indicated an increase in emotional wellbeing (p<0.01), while 5.7% runners reported no effect and 1.8% reported a reduction in emotional wellbeing. Results did not report the scale of improvement. More than half the runners (54.8%, n=249) indicated an increase in general tolerance of others (p<.01), while 33.7% of runners indicated no effect and 6.8% had reduced tolerance of others. Results found that age and emotional well-being were significantly correlated (gamma value of 0.42, p<0.001), that is the older the runner, the greater the perception of emotional well-being resulting from running. The inverse relationship between average hours per week running and emotional wellbeing was highly significant (gamma value = -0.43, p<.001). Overall, results suggest that running increases emotional wellbeing[3].
4	Valliant (1981)	compare self-sufficiency and personality profiles in marathon runners vs recreational joggers	A Canadian cross sectional study by Valliant et al. (1981) used 68 male participants to compare self-sufficiency and personality profiles in marathon runners (n=30, mean age 34.4) vs recreational joggers (n=38, mean age 20.6) using a one hour 'Sixteen Personality Factor Questionnaire' as measurement. Marathoners were on average more reserved (F=17.07, df=1,66, p<0.001), intelligent (F = 12.69, df=1,66, p<0.001), tender-minded (F = 11.79, df = 1,66, p<0.001), imaginative (F=11.09, df=1,66, p<0.005), and self-sufficient (F = 19.84, df=1,66, p<0.001) than joggers. Conversely, joggers were more happy-go-lucky (F = 10.05, df = 1,66, p<0.005), apprehensive (F = 10.51, df=1,66, p<0.005) and controlled (F=7.09, df=1,66, p<0.01). Marathoners were on average significantly older (F=99.45, df=1,66, p<0.001), ran more miles per week (F=167.6, df=1,66, p<0.001) and trained for more years (F=20.55, df=1,66, p<0.001) than the joggers. Overall results found that marathon runners had a more self-sufficient personality as compared to joggers who were less assertive, more conscientious and controlled personality types[4].

**5**	Francis (1982)	Comparing anxiety, depression and hostility in various groups of runners vs sedentary controls	An American cross sectional study by Francis et al. (1982) used 44 male participants with a mean age of 32 to compare anxiety, depression and hostility in various groups of runners vs sedentary controls using the State-Trait Anxiety Inventory (STAI) and the Multiple Affect Adjective Check List (MAACL) as measurements. Participants were separated into 4 groups based on the number of miles jogged per week: non-running controls who ran 0 miles weekly (n=16), 20 miles (n=10), 30-40 miles (n=8) and 50-60 miles (n=10). There were no significant differences in psychological variables when jogging groups (ie. Miles jogged per week) were compared to each other. Spearman correlations for the psychological parameters were as follows: MAACL anxiety= -0.08, MAACL depression= -0.15, MAACL hostility= 0.11, STAI anxiety= -0.12, none of which were significant. Runners had significantly lower (p<0.01) anxiety, hostility and depression than their sedentary counterparts. Combined joggers scores vs sedentary control scores for MAACL anxiety were 4.2 vs 7.2, respectively, for MAACL hostility were 4.8 vs 6.8, for MAACL depression were 8.6 vs 12.3, and for STAI anxiety were 30.8 vs 42.8. Combined trait scores revealed that anxiety, hostility, and depression as measured by the MAACL in joggers were respectively 27%, 23% and 14% lower than normative scores (reported in other papers) and anxiety as measured by the STAI was 19% lower in joggers when compared to the reported norms. Overall results suggest that running lowers depression and anxiety measures[5].
**6**	Hailey (1982)	investigate the relationship between running and negative addiction	An American cross sectional questionnaire by Hailey et al. (1982) used 60 male runners aged between 13 and 60 years old to investigate the relationship between running and negative addiction using the negative addiction scale as measurement. The subjects were split into three groups: those who had run for less than 1 year (n=12), those who had ran for 1-4 years (n=32) and those who had ran for over 4 years (n=16). Overall, the sample mean for negative addiction scores was 5.39 on a scale of 1 to 14. There was significant difference in negative addiction between the groups ($F_{(2,58)}$= 3.48, p<.05), with length of running history associated with increasing negative addiction scores. Runners with a running history of less than 1 year scored a mean of 3.84, those running for 1-4 years scored 5.63, and those running for over 4 years scored 6.38. Addiction scores for runners of over 4 years was greater than the addiction score for runners of under one year (t(59)=2.72, p<.005). Likewise, the addiction score for runners of between one and four years was greater than the score for runners under one year (t(59)=2.52, p<.01). However the difference in addiction scores between the 1-4 year group and the 4+ year group was not statistically significant, which may suggest that negative addiction reaches a plateau with running and does not increase at the same rate in later stages as it does in the beginning of the development of running behaviour with significant differences between the groups. Overall, results suggest that the more years a male has been running, the greater the risk of developing negative addiction[6].
**7**	Callen (1983)	Investigating mental and emotional aspects associated with long-distance running in non-professional runners, including depression, tension, mood, happiness, self-confidence and self-image.	An American cross sectional study by Callen (1983) used 424 non-professional runners (303 men and 121 women) with a mean age of 34 years old and who ran on average more than 28.8 miles per week to investigate the mental and emotional aspects associated with long-distance running, including depression, tension, mood, happiness, self-confidence and self-image. A questionnaire designed by the author was used as measurement. 96% of the subjects noticed mental or emotional benefits from running, however no details were reported of the size of these mental and emotional benefits. Benefits included relief of tension (86% of all respondents, 88% of men and 82% of women, ns for all three); improved self-image (77% of all runners, 74% of men and 82% of women, ns for all three); better mood (66% of all participants, 62% of men (p<0.05) and 82% of women(p<0.05)), improved self-confidence (64% of all participants, 63% of men and 65% of women, ns for all three), relieved depression (56% of all participants, 52% of men (p< 0.05) and 69% of women (p < 0.05)) and improved happiness (58% of all participants, 56% of men and 64% of women). However, 25% state that they had experienced emotional problems associated with running, in which almost every instance the problem is one of depression, anger, or frustration associated with not being able to run due to an injury. No further details were reported on this. 69% of runners experienced an emotional "high" associated with running[7].
**8**	Galle (1983)	Comparing psychologic profiles including anxiety and depression in runners, infertility patients, fertile	An American controlled cross sectional questionnaire by Galle et al. (1983) used 391 female subjects to compare psychologic profiles including anxiety and depression in runners (n=102), infertility patients (n=103), fertile subjects (n=139) and Clomid study patients whose only infertility abnormality was ovulation dysfunction (n=47), using the Hopkins Symptom Checklist-90 (SCL-90) as measurement. The runners were aged 15 to 50 years, 15% had amenorrhea, 70% had regular cycles, and 15% had irregular cycles. The SCL data showed that the mean scores in all groups-runners, Clomid study patients, infertility patients, and fertile control subjects were in the normal range for all factors. Mean total scores for SCL did not vary significantly amongst the 4 groups (F=1.19, ns),

		subjects and Clomid study patients whose only infertility abnormality was ovulation dysfunction	however, there was a significant difference for the depression subscale (F=3.42, p<0.025): the depression scores of runners (were nearly identical to those of fertile control subjects but were significantly lower than the depression scores of the Clomid study patients or the infertility patients. The only significant difference between runners and fertile control subjects was that control subjects had higher hostility (p<0.05). Significant differences were noted with the factors of obsessive-compulsive behaviour (p< 0.01) and psychoticism (p<0.005). The women running more than 30 miles per week had higher mean scores for all factors, with significant differences in somatization (P<0.05) and anxiety (P<0.005). Regarding just runners, there was significant differences in depression between amenorrhoeic (n=15) and regular cycling runners (n=87), with amenorrhoeic runners scoring higher in the depression factor than regular cycling runners (F=3.0, p<0.10). Overall the emotional distress scores of runners were not significantly different from fertile control subjects, but both groups of infertility patients showed greater distress on items in the depression factor than the runners and fertile control subjects. Emotional distress factors were associated with the development of amenorrhea in these runners[8].
**9**	Lobstein (1983)	Impact of a treadmill run with increasing gradient on depression	An American pre-post controlled between subject design by Lobstein et al. (1983) used 22 medically healthy men aged 40-60, to compare depression in physically active men (n=11) to sedentary men (n=11) using the Minnesota Multiphasic Personality Inventory (MMPI) as measurement. The MMPI indicated that sedentary men were more depressed (mean 61.36) than the physically active men (mean 50.73) with p < 0.01 and standardized canonical coefficients of 0.929. However, both groups of men were within clinical limits for normal, mentally healthy, middle aged men[9].
**10**	Rudy (1983)	investigating how levels of anxiety and self-esteem related to intensity of jogging	An American cross sectional questionnaire by Rudy et al. (1983) used 319 female regular runners between the ages of 16 and 60 to investigate how levels of anxiety and self-esteem related to intensity of jogging using the Rosenberg Self-esteem Scale and Zuckerman's Anxiety Adjective Checklist as measurements. Results found that female runners jogging with great intensity demonstrated significantly less anxiety (x^2 = 22.83; p<.001). In addition 14% of women listed decreased tension as a result of jogging they felt others should know about. No significant relationship was drawn between self-esteem score and intensity of jogging, however the majority (89%) of women scored in the range designated as high self-esteem, and there was evidence that jogging influenced self-esteem in the open-ended answers with 29% of responses stating they feel better about themselves, 12% stating they have increased self-confidence and 6% stating they had a sense of accomplishment. Hence this paper does show evidence that jogging influences self-esteem, just not significant evidence[10].
**11**	Goldfarb (1984)	Investigating anorexia nervosa traits within distance runners	An American cross sectional study by Goldfarb et al. (1984) used 200 distance runners (136 men and 64 women) to investigate anorexia nervosa traits within distance runners using the Goldfarb Fear of Fat scale and Activity Vector Analysis to measure personality characteristics. The study does not give details on the demographics of the participants. Results do not support a connection between running and fear of fat, a central component of anorexia nervosa, with only 29 (14.5%) participants reporting a high fear of fat score (i.e. between 6 and 10 on the scale). Overall, the mean fear of fat score for these runners was 2.91, indicating a low-normal fear of fat. Fear of fat scores did not correlate significantly with any of the measures of running zealousness including miles run per week (r=-.04), number of workouts per week (r=.09), number of road races (r=.05), or marathons completed (r=-.05), and degree of importance placed on running (r=-.03). The runners who demonstrated the greatest zealousness demonstrated AVA profiles that closely clustered around one particular profile (r=.64, p< .05) consisting of high ipsative scores on aggressiveness and dependence and low ipsative scores on sociability and emotional stability… indicating that these individuals are assertive, obsessive, perfectionistic, and anxious. Overall, results do not support a correlation between running and fear of fat. However, the runners most closely resembling "obligatory runners" exhibited traits characteristic of anorexia nervosa patients[11].
**12**	Guyot (1984)	comparing death anxiety in runners vs non-runners	An American controlled cross sectional study by Guyot et al. (1984) used 126 participants to compare death anxiety in runners (44 males and 20 females) vs non-runners (37 males and 25 females) using the Death Concern Scale as measurement. The study did not give details on the demographics of the participants. Runners scored significantly higher (mean= 19.5) than nonrunners (mean= 17.6) on the death thoughts subscale within the Death Concern Scale ($F_{(1,122)}$ =4.49, p<.05), meaning that runners reported thinking more about death than nonrunners. However, nonrunners scored significantly higher (mean =12.5) than runners (mean =10.8) on the death anxiety subscale ($F_{(1,122)}$=6.35, p<.05), indicating that nonrunners had more anxiety about death than runners. Sex of the subject was not significant in either analysis and there were no significant interactions. The number of years running, which averaged 5.5 years for male runners and 4.9 years for female runners did not significantly correlate with either

			death thoughts subtotal (r=-.04) or death anxiety subtotal (r=-.04). Overall results found runners experienced more death thoughts but less death anxiety than nonrunners [12].
**13**	Rape (1987)	Comparing depression scores in runners vs non-exercisers	An American controlled cross sectional study with a matched two-group design by Rape (1987) used 42 male participants between the ages of 18 and 25 to compare depression scores in 21 runners (ran 15 or more miles weekly) vs 21 non-exercisers using the Beck Depression Inventory as measurement. Results found that the runners were significantly less depressed [M=4.38, SD = 3.88] than the non-exercisers [M=9.55, SD = 5.40]; (t_{40}= 3.55, p<0.001). Overall results suggest that running reduces depression [13].
**14**	Weight (1987)	comparing eating attitudes and disorders in marathon runners vs cross country runners vs non-running controls	A South African cross sectional controlled study by Weight et al. (1987) used 135 female participants between ages 18-56 to compare eating attitudes and disorders in marathon runners (n=85) vs cross country runners (n=25) vs non-running controls (n=25) using the Eating Attitudes Test (EAT) and the Eating Disorder Inventory (EDI) as measurements. One way ANOVA of the different groups showed no significant difference between any group on any of the EAT sub-scores (P<0.05), with mean EAT scores for the marathoners, cross country runners and non-running control at 8.4, 14.3 and 11.8, respectively. The EDI scores did not follow a definite pattern, with all groups showing a gradual, if erratic downward trend. Mean EDI scores for the marathoners, cross country runners and non-running controls were 24.8, 27.1 and 32.0, respectively. All subjects with high EAT scores (>20) also had high EDI scores (>30) but there was no relationship between high EDI scores and the EAT scores. Overall results found that abnormal eating attitudes and the incidence of anorexia was no more common among competitive female runners than it is among the general population, with a low incidence of anorexia in the total group (2 out of 135 participants) [14].
**15**	Chan (1988)	Comparing depression, self esteem and mood in prevented runners vs continuing runners	An American cross sectional questionnaire by Chan et al. (1988) used 60 runners (32 women and 28 men) aged between 15 & 50 who had ran consistently (at least 3x per week) for a minimum of a year and more than 20 miles per week when not injured. The study compared depression, self-esteem and mood in 30 prevented runners (unable to run for 4 weeks due to a running-related injury) to 30 continuing runners (ran without interruption) using the Zung depression Scale, Rosenberg Self-esteem Scale and Profile of Mood States as measurement. Prevented runners reported significantly greater over-all psychological distress than the continuing runners group (Wilks's =0.63, p<.01: X_9^2 = 24.38, p<.01). Regarding Zung Depression Scale scores, the prevented runners were significantly more depressed than the continuing runners ($F_{(1,58)}$= 11.57, p<0.01). Based on POMS total score, prevented runners reported a significantly greater over-all mood disturbance than the continuing runners group ($F_{(1,58)}$ =11.03, p<.01). vs 42.60). On the Rosenberg Self-esteem scale, the prevented runners reported significantly lower self-esteem than the continuing runners ($F_{(1,58)}$ =3.17, p<.05). Prevented runners reported that they were less satisfied with the way their bodies presently look ($F_{(1,58)}$=4.17, p<.05) and had a greater desire to change something about the way their bodies presently look ($F_{(1,58)}$ =4.54, p<.05) compared to continuing runners. Overall, results suggest that preventing running in regular runners increases depression, overall mood disturbance, as well as decreasing self-esteem and body confidence [15].
**16**	Frazier (1988)	Investigating the relationship between running and mood in regular distance runners	An American post only, non-randomised, long term observational study that is unlikely to have made any controls for confounding, by Frazier (1988) used 86 regular, distance runners who had all completed a marathon (68 males with mean age of 33.7 and 18 females with a mean age of 32.2) to investigate the relationship between running and mood using the Profile of Mood States as measurement. The running subjects had lower mean scores on tension, depression, anger, fatigue and confusion, and a higher mean of vigor compared to scores for test norms, however statistical significance between the runners and the norm values was not reported. female subjects recorded higher mean scores on all sex states (tension, depression, anger, vigor, fatigue, confusion), however, only a significant difference was noted on confusion between females (mean =7.8) and males (5.5) ($F_{(1,84)}$ = 5.33, p<.05). Overall results suggest that regular, distance running improves mood in both males and females [16].
**17**	Lobstein, Ismail (1989)	Comparing anxiety and depression levels in runners vs sedentary controls	An American controlled cross sectional study by Lobstein, Ismail et al. (1989) used 36 male participants aged between 40 & 60 years old to compare anxiety and depression levels in runners (n=21) vs sedentary controls (n=15) using the Minnesota Multiphasic Personality Inventory (MMPI) and Eysenck Personality Inventory as measurements. In MMPI scores, both groups appeared to be within psychologically normal limits, however, physically active men exhibited significantly less anxiety than sedentary men (mean = 48.95 vs 61.48 respectively, p<0.05, standardised canonical coefficient = -1.07) and less depression compared to the sedentary men (mean = 50.76 vs 57.93, respectively, p<0.05, standardised canonical coefficient = 0.00). Discriminant function analysis showed that anxiety index was the most powerful discriminator between the physically active and sedentary men (standardised canonical coefficient =-1.07). Neurotocism score (Eysenck) was not significant between the physically active group

			and the sedentary group (4.95 vs 6.20) (standardised canonical coefficient = -0.72). High physical fitness scores were correlated with low depression (r=-0.40, p<0.05). Overall results indicate that running reduces anxiety and depression compared to being sedentary[17].
**18**	Lobstein, Rasmussen (1989)	Comparing depression and stress in sedentary men to physically active joggers	An American cross sectional study by Lobstein, Rasmussen et al. (1989) used 20 psychologically normal, medically healthy men, aged between 40 & 60, to compare depression and stress in sedentary men (n=10) compared to physically active joggers who had been running about 20 miles per week for at least 3 years (n=10) using the Eysenck Personality Inventory (EPI) and Minnesota Multiphasic Personality Inventory (MMPI) as measurements. EPI scores demonstrated that the joggers (mean=2.80) exhibited significantly more emotional stability than the sedentary group (mean=7.10) (t=-2.84, p<0.01). Regarding the MMPI profile, both physically active and sedentary group profiles were within clinical limits for psychologically normal middle-aged men. The MMPI subscales of depression and Wiggins depression were both significantly lower in the joggers (t=3.70,p<0.01; t=2.40, p< 0.05; respectively) indicating that the physically active men were less depressed than the sedentary me. The magnitude and direction of the canonical coefficients (0.98) indicated that the subjective depression subscale appeared to be the most powerful discriminator between the two groups. Overall the findings suggest that regular jogging decreases subjective depression and increases emotional stability[18].
**19**	Nouri (1989)	investigating the relationship between various levels of jogging vs non-exercising on anxiety and addiction/ commitment	An American cross sectional study by Nouri et al. (1989) used 100 male participants aged between 18 and 62 to investigate the relationship between various levels of jogging vs non-exercising on anxiety and addiction/commitment using the Commitment to Running Scale, The Buss-Dutkee Inventory measuring hostility and aggression and the Spielberger State-Trait Anxiety Inventory as measurements. Participants were divided into 5 groups: non-exercisers (n=28), drop-out joggers (n=21), beginning joggers (n=15), intermediate joggers (n=16), 20 advanced joggers (n=20). Commitment to Running gave a main effect for level of jogging ($F_{(4,89)}$ = 14.30, p<.01). Advanced, Intermediate and Begging joggers all scored higher than drop-outs or non-exercisers on the Commitment to Running Scale. However, there was no statistically significant difference between non-exercisers and drop-out joggers or among the other jogging groups. ANOCA for trait anxiety scores was significant ($F_{(4,89)}$ = 4.43, p<.01). Non-exercisers had higher mean scores on trait anxiety than advanced, intermediate, bigger and drop-out joggers (2.00, 1.42, 1.69, 1.77 and 1.68, respectively). Advanced joggers had the lowest mean trait-anxiety score (1.42) and were significantly lower than the other groups p<.01). Overall results suggest that running reduces anxiety levels compared to physical inactivity, with advanced joggers having even less anxiety than beginner and intermediate joggers[19].
**20**	Chan (1990)	Investigating a relationship between running and depression, stress, tension and personality profiles	A Hong Kong based cross sectional study by Chan et al. (1990) used 44 male, Chinese runners with a mean age of 27.8, who all except 1 belonged to a single track club, who ran for a mean of 4.66 years and ran a mean of 57.2km per week. The study investigated a relationship between running and depression, stress, tension and personality profiles, using a Chinese version of the Personality Research Form and a questionnaire designed by the authors to assess running history and experience. 36.4% of participants reported 'improving mental health' as a reason to starting running. Emotional benefits from running reported were: more self-confident (59.1% of respondents), happier (56.8%), better mood (50.0%), relieved tension (45.5%), better self-image (36.4%), relieved depression (36.4%), more aggression (36.4%), improved outlook (34.1%), more content (31.8%) and better family relationship (15.9%). However, when participants stopped running 38.6% experienced low mood and 25.0% experienced anxiousness. Significance was largely not reported on throughout the results. Results inferred that the typical male runner was more controlled and less oriented intellectually and aesthetically. More experienced runners, compared to less experienced runners, were less aggressive or easily angered (t=2.92, df=42, p<0.01), less guarded or defensive (t=2.13, df=42, p<0.005), and more likely to present themselves favourably (t=2.68, df=35, p<0.05). Overall, results suggested that running increased mood, happiness and outlook, while relieving depression, aggression and anger, however there was no reporting of the size of these changes or their significance[20].
**21**	Chapman (1990)	investigating the relationship between running addiction, psychological	An American cross sectional study by Chapman et al. (1990) used 47 runners (32 males aged 34-57, and 15 females aged 35 to 59) to investigate the relationship between running addiction, psychological characteristics and running using the Running Addiction Scale (RAS), Commitment to Running Scale (CR), Symptom Checklist (SCL-90-R) and Levenson's Locus of Control Scale as measurements. RAS correlated for both sexes of runners strongly with self-rated addiction (p<0.05) and moderately with discomfort (p<.05). However, CR did not significantly correlate with self-rated addiction

		characteristics and running	in females (.246, ns) while the RAS did (.753, $p<.05$) ($z=2.00$, $p<.05$). Running addiction (RAS) was found to be associated with high frequency of running ($p<.05$) and longer duration of running (males=$p<.05$; females= ns). The CR score correlated significantly with run frequency for the male (.59, $p<0.05$) but not the female runners (.14, ns), while CR and run duration did not correlate significantly for either sex (males=.16, females=.28, ns for both). Male runners were above the norm for obsessive compulsive tendencies (SCL-90 score) and significantly higher than female runners ($p<.05$). The female runners were above the norm in hostility ($p<.05$) and interpersonal sensitivity ($p<.05$) and significantly higher than males ($p<.05$). For males, correlations indicated a significant relationship between positive personality characteristics and addiction, high frequency and long duration running and psychological health ($p<0.05$). There were no significant correlations with personality traits for females. Overall, results indicates that for female runners commitment to running can occur without addiction and that there is a sex difference in the relationship between addiction and commitment. Running addiction was found to be associated with male positive personality characteristics but not with mood enhancement. While the duration of running was found to be associated with mood enhancement implying that the benefits of running to mood may be obtained without addiction[21].
**22**	Guyot (1991)	Investigating the relationship between addiction and death anxiety between pain runners and non-pain runners	An American cross-sectional questionnaire by Guyot (1991) used 370 runners to investigate the relationship between addiction and death anxiety between pain runners and non-pain runners using the Dickstein Death Concern Scale and author created questionnaires for pain running, running motives, risk taking and medical symptoms as measurements. Participants consisted of 78% males, who had a mean age of 38, ran 33.3 miles per week and had been running for an average of 7.9 years, and 22% females, who had a mean age was 35, ran 21.3miles per week and had been running for an average of 6.3 years. 56% of the 370 runners pushed themselves during running until they felt pain: 60% of the males and 43% of female runners were classified as pain runners. This difference between genders was significant ($p=0.008$). Compared to non-pain runners, pain runners were more likely to be male, taller ($F_{(1,366)}=11.45$, $p<0.05$), heavier ($F_{(1,366)}=9.19$, $p<0.05$), and younger ($F_{(1,366)}=5.75$, $p<0.05$). Pain runners were more likely to be running for competition (58% of PR vs 42% of NPR) and less likely for improved health (75% of PR vs 84% of NPR) ($p<0.05$ for both). Pain runners were significantly higher on 17 of 23 (74%) medical symptoms than non-pain runners ($p<0.05$). Pain runners reported significantly more death thoughts Dickstein Death Concern Scale (Mean = 16.77) than non-pain runners (Mean = 15.78) $F_{(1,364)}= 5.04$, $p<0.05$, as well significantly more death anxiety (Mean = 10.95) than non-pain runners (Mean = 9.66), $F_{(1,364)}= 8.86$, $p<0.05$. Overall, results suggest that runners classified as pain runners were experienced significantly more death thoughts and death anxiety than non-pain runners[22].
**23**	Maresh (1991)	Investigating psychological characteristics including anxiety, depression and stress in distance runners	An American cross sectional study by Maresh et al. (1991) used 29 male, distance runners with a mean age of 40.1 who had been running for an average length of 11.8 years prior to the study, to investigate psychological characteristics including anxiety, depression and stress using the Myers-Briggs Type Indicator Form to determine personality characteristics and the Multidimensional Anger-Inventory as measurements. Results suggested that long term involvement in running is associated with low levels of self-reported anxiety (m=2.5 on a 6 point scale), depression (M=1.8) and stress (m=2.5). 82% of the male runners reported suffering from withdrawal symptoms when forced to be inactive, with the level of self-reported addiction average 4.4 ('moderately' to 'very') on a 6 point scale. Withdrawal symptoms were experienced 5.0+/-6.2 days after exercise ceased. A majority (55%) of those experiencing withdrawal did so within 3 days. Compared to a normative sample of male control students, the runners were less angry overall and were less frequently angry across fewer situations. Runners also reported very low scores on hostile outlook, however there were no differences between the two samples on brooding, guilt over anger, or tendencies to turn anger inward against the self. The subjects personality profiles differed markedly from the normative sample, with men in the general population tending to be more extraverted (75%) than introverted (25%), more sensate (57% than intuitive (25%), more thinking (60%) than feeling (40%), and equally split between judging (50%) and perception (50%). Overall results suggest that running is associated with a positive sense of self; reduced anxiety, depression and stress; and more introverted personalities. However, many runners experienced withdrawal symptoms if forced to be inactive[23].
**24**	Gleaves (1992)	Comparing depression, body image disturbance and	An American controlled cross sectional study by Gleaves et al. (1992) used 60 female participants to compare depression, body image disturbance and bulimia nervosa symptomology in runners (n=20), bulimia patients (n=20) and a non-exercising, non-dieting control group (n=20) using the Beck's depression inventory (BDI) Automatic

		bulimia nervosa symptomology in runners, bulimia patients and a non-exercising, non-dieting control group	thoughts Questionnaire (ATQ), subscales from the Eating Disorder Inventory (EDI) (Ineffectiveness scale, Drive for Thinness scale, Bulimia scale, and Body dissatisfaction scale), a Bulimia test, Body Image Assessment Procedure, and a dieting/weight loss questionnaire as measurements. For depression scores the overall MANOVA was significant, F(6110.25) = 14.76, P < 0.0001. Bulimics scored significantly higher for BDI depression than the runners and controls (20.65, 3.30 and 4.80 respectively, F=56.95, p<0.0001), but runners and controls did not differ from each other. The same pattern of results was found for the ATQ and the EDII with bulimics differing from the two other groups and no significant difference between runners and controls: ATQ (F=45.87, p<0.0001), means for runners =41.10, controls=41.50, bulimics = 85.40; EDII (F=34.95, p<0.0001), means for runners =0.80, controls=1.60, bulimics = 12.80. There were significant group effects on all four dependent variables of bulimia (p<0.001), with bulimics scoring higher compared to the other groups who did not differ among themselves. There were significant group effects for all three variables of body image (p<0.01), again, bulimics differed from runners and controls. Overall, results did not indicate that running leads to development of disordered eating or problems with body image, but instead that runners are generally indistinguishable from control subjects with no differences found between runners and controls throughout the study. Bulimics were significantly more disturbed than runners or controls[24].
**25**	Coen (1993)	investigate the relationship between obligatory running vs non-obligatory running on anxiety, anorexia and self-identity	An American cross sectional study by Coen et al. (1993) used 142 male marathon runners with a mean age of 44.07 to investigate the relationship between obligatory running (n=65) vs non-obligatory running (n=77) on anxiety, anorexia and self-identity using the Obligatory Exercise Questionnaire (OEQ), State-Trait Personality Inventory and The Ego Identity Scale (EIS) as measurements. The obligatory runners had a mean total OEQ score of 56.64; the non-obligatory group had a mean score of 47.60. There was a statistical difference between (OEQ) scores of the obligatory and the non-obligatory runners ($t_{(140)} = 13.19$, p <0.001), with the obligatory group running more miles per week and spending more time running each week. The obligatory group had significantly higher (p<0.01) mean levels of anxiety than the non-obligatory group (18.85 vs 6.45, respectively), indicating that obligatory runners appear to be more perfectionistic and to have higher levels of trait anxiety than non-obligatory runners. Although the non-obligatory runners had an average Ego Identity Scale score that was higher than the obligatory group (means 8.68 vs 8.34, respectively), the difference was not statistically significant (p>0.05) indicating that neither group showed a higher developed sense of identity. Overall results suggest that running represents a successful coping mechanism to reduce anxiety and becomes problematic only when the obligatory individual is unable to run because of injury or other circumstances[25].
**26**	Furst (1993)	Comparing negative addiction in runners vs gym exercisers	An American controlled cross sectional study by Furst et al. (1993) used 188 subjects to compare negative addiction in runners (72 male & 26 female runners, 82% white, 42% were aged between 20-29 y/o, 36% between 30 and 39 y/o) to gym exercisers (60 male & 30 female, 95% were white, 42% were aged between 20 & 29, 36% between 30 & 39) using the Negative Addiction Scale as measurements. Subjects were divided into 6 groups by years of participation and a significant difference was found between years of physical activity and addiction scores ($F_{(5,182)} = 6.39$, p<0.01) indicating that longer involvement in physical activity was associated with higher addiction scores. When runners were compared with gym exercisers, there were no significant differences in mean addiction scores. Only 5 people scored 9 or above on the negative addiction scale (ranges from 0 to 14), indicating that none of the 188 participants were extremely addicted to the activity. Overall results suggest that the longer people had been exercising, both runners and gym goers, the more addicted they were to exercise[26].
**27**	Masters (1993)	Investigating self-esteem and psychological coping of marathon runners	An American cross sectional study by Masters et al. (1993) used 712 participants in a marathon (601 men and 111 women) all aged between 16 and 79 to assess self-esteem and psychological coping of runners using the Motivation of Marathoners Scales (MOMS), Sport Orientation Questionnaire, Marlowe-Crowne Social Desirability Scale, Attentional Focusing Questionnaire (AFQ) and 3 body satisfaction and composition questions. There were significant positive correlations between the AFQ dissociation and the MOMS psychological coping [r(66)=.54, p<.001], self-esteem [r(66)=.31, p<.01] and life meaning scales [r(66)=.36, p<.01]. Marathon runners reporting higher anxiety levels were more likely to endorse psychological motives for marathon running, indicating that their running helps them avoid or dampen negative emotional experiences: psychological coping, [r(62)= .38, p<.01] and self-esteem, [r(62)=.36, p<.01]. A t-test comparing average rating on the weight concern scale for the two genders was calculated. Women had a higher mean score than men, and women more strongly endorsed weight concern as a reason for involvement in marathons [t(588)= -3.52, p<.001]. No significant relationship was found indicating that social desirability played a major role in subjects responses to the MOMS. Personal goal achievement and

			competition were both positively related to training miles per week [r(575)=.22, p<.001 and r(576) =.30, p<.001, respectively]. Overall, results suggest that participation in marathon running and training was used as a way of problem solving, providing self-distraction and improving mood and self-esteem[27].
**28**	Pierce (1993)	Comparing exercise dependence in recreational (non-competitive) runners vs 5km runners vs marathoner runners	An American cross sectional questionnaire by Pierce et al. (1993) used 89 male runners to compare exercise dependence in recreational (non-competitive) runners (n=33) vs 5km runners (n=24) vs marathoner runners (n=32) using the negative addiction scale as measurement. Marathoners showed significantly higher (p<0.05) mean exercise dependence scores (3.78) compared to 5K (2.9) and recreational runners (2.16). There was no significant difference in exercise dependence scores found between recreational and 5K runners. Comparisons between competitive groups and variables of exercise addiction and miles per week yielded correlational coefficients of +0.68 and +0.81, respectively. Overall results show that training mileage was significantly correlated with exercise dependence and competitive orientation[28].
**29**	Klock (1995)	Comparing depression, anorexia nervosa, excessive exercise and eating disorders in amenorrhoeic runners, eumenorrheic runners and eumenorrheic sedentary women as controls	An American controlled cross sectional study by Klock et al. (1995) used 22 females who were not currently pregnant or taking oral contraceptives to compare depression, anorexia nervosa, excessive exercise and eating disorders in amenorrhoeic runners (n=7, mean age 28.0), eumenorrheic runners (n = 9, mean age 32.1) and eumenorrheic sedentary women as controls (n=6, mean age = 27.5) using the modified Body Image Questionnaire (BIQ), the Beck Depression Inventory (BDI), the Symptom Checklist-90 (SCL-90) and the Eating Disorders Inventory (EDI) as measurements. All 3 groups had overall satisfaction with general body appearance and there were no significant differences regarding body satisfaction. No differences were found among groups on the BDI, however the amenorrhoeic runners' mean score was double that of the eumenorrheic runners and sedentary controls (8.3 versus 3.8 and 3.0, respectively) but it was below 11 which is the lowest score indicative of mild depression. No significant differences found between groups on the SCL-90 scores or total EDI scores, although the amenorrhoeic runners' mean EDI score was at the level indicative of a clinically significant eating disorder. 3 of the 9 amenorrhoeic runners scored in the clinically depressed range on the BDI, indicating that they were mild to moderately depressed, and also had the highest scores in their group on the SCL-90 and the EDI. Overall results found no significant differences between amenorrhoeic runners, eumenorrheic runners, and eumenorrheic sedentary controls on any of the psychological measures, hence these results do not suggest that there are psychological similarities between obligatory runners and anorexics. However, there was a subgroup of amenorrhoeic runners who scored in the extreme range on the depression and eating disorder measures[29].
**30**	Thornton (1995)	Investigating a relationship between habitual running and addiction	A UK based cross sectional questionnaire by Thornton et al. (1995) used 40 long-standing, habitual male runners with a mean age of 38 and who ran on average 4 times a week with a weekly mileage of 42.5miles, to investigate a relationship between habitual running and addiction using the Rudy and Estok Running Addiction Scale (RE/RAS), the Hailey and Bailey Running Addiction Scale (HB/RAS) and the Personal Incentives for Exercise questionnaire (PIE) as measurements. The majority (77%) of subjects were committed to levels of running which would be classified as moderately (scores 13-20) or highly 'addictive' (scores +20) (55% and 22%, respectively). The correlation between the two addiction scales revealed a strong positive relationship (r_s = 0.81; p<0.001). The primary motivation for running was mastery (mean PIE score of 4.2) followed by competition (3.93), weight regulation (3.9), health benefits (3.89), fitness (3.87) and social recognition (3.01). For RE/RAS scores, only two variables, mastery ($F_{(1,38)}$ = 12.1, p < 0.001) and social recognition ($F_{(2,37)}$ = 9.4, p < 0.001) contributed to the predictive equation. In a second regression analysis for the HB/RAS scores, mastery was again entered as the first step ($F_{(1,38)}$ = 16.5,p < 0.001), with both social recognition ($F_{(2,37)}$ = 11.8, p< 0.001) and distance ($F_{(3,36)}$ = 11.6, p < 0.001) providing significant contributions. A final regression analysis was performed to predict 'distance run' according to both addiction scales and PIE subscale scores. The model entered two variables with the HB/RAS addition scores as the initial variable ($F_{(1,38)}$ = 8.1, p< 0.01; R^2 = 0.18), and social recognition in the second step ($F_{(2,37)}$ = 11.3, p< 0.01; R^2=0.06). There was no relationship between years of running and either of the addiction scales. This contrasts with the significant correlations between both the RE/RAS and the frequency of running (r_s = 0.38; p < 0.05) and the HB/RAS scale and the number of runs per week (r_s = 0.55; p < 0.01). The effect of mileage run was related only to the HB/RAS (r_s =0.39; p <0.05). Overall results found a high level of commitment in the sample of runners, but there was no relationship between years of running and addiction measured by the regression analysis[30].
**31**	Powers (1998)	Comparing psychological	An American controlled cross sectional study by Powers et al. (1998) used 57 participants to compare psychological profiles of habitual male runners (n=20), habitual

		profiles of habitual male runners, habitual female runners and female anorexia nervosa patients	female runners (n=20) and female anorexia nervosa patients (n=17) using the Minnesota Multiphasic Personality Inventory (MMPI), Leyton Obsessional Inventory, Obligate Running Questionnaire, Becks Depression Inventory and three body image tests (open door test, body parts satisfaction test and colour-a-person body dissatisfaction) as measurements. In the open door test there were significant differences between the groups (F=7.969, p<.001) but no significant differences between the female groups. On the Body Parts Satisfaction Questionnaire, male runners were significantly more satisfied with their bodies than female runners, who were significantly more satisfied than anorexics. In the ORQ item "I worry almost constantly that I will get fat" anorexics were more likely to answer true, male runners more likely to answer false and female runners answered true as often as they answered false (p=0.001). In the MMPI subscale scores anorexics scored significantly higher than either group of runners (p<.001) for the nine subscales except scale 9 (p=.0001). Mean T scores were above 70 (considered clinically significant) for subscales of depression, hysteria and psychopathic deviate in the anorexic group, while none of the mean scores for either set of runners were considered clinically significant. There were significant differences in depression scores (F=68,645, p=.0001) with anorexics scoring significantly higher (p<.0001) than both male and female runners (mean scores were 23, 2.4 and 3.45, respectively) but there was no significant differences between the runners. While there were suggestive similarities between female runners and anorexics on body image, the overall results found few psychological similarities between anorexia patients and habitual runners with evidence of significant psychopathology on all psychological measures in the anorexia group, while both groups of runners were consistently in the normal range[31].
**32**	Slay (1998)	Comparing eating pathology traits between obligatory and non-obligatory runners	An American cross sectional questionnaire by Slay et al. (1998) used 324 regular runners (240 males and 84 females) between the ages of 15 and 71 to compare eating pathology traits between obligatory and non-obligatory runners using the Eat Attitudes Test (EAT) and Obligatory Running and Motivations for Running Questionnaire as measurements. 21 women (25%) and 63 men (26.2%) were classified as obligatory runners. There was a significant effect for miles run per week [$F_{(1,164)}$ =8.31, p<0.001], with men running more than women [p<0.05] and obligatory runners with higher mileage than non-obligatory runners [p<0.001]. Obligatory runners scored significantly higher on the EAT test, with female obligatory runners having the highest mean EAT score. A partial correlation, controlling for miles run per week, between the EAT and obligatory running scores for men was slightly weaker (r=.28, p<.0001) than for women (r=.40, p<.0002), showing a stronger relationship of obligatory running with eating pathology in women than in men. Results found women and men scored similarly on the EAT with no significant differences at low levels of obligatory running [$F_{(1,164)}$ =2.78, p>.05]; however at higher levels women demonstrated significantly higher EAT scores than did men [$F_{(1,164)}$ =29.50, p<.001]. Independent of miles run per week, there was still significant overall effect on EAT scores, [$F_{(1,164)}$ =9.65, p<.0001] and a sex/obligatory running interaction, [$F_{(1,164)}$ =8.02, p<.05]. Overall results suggest that obligatory runners, particularly females are most at risk of eating pathophysiology[32].
**33**	Ryujin (1999)	Comparing eating disorder symptomology in collegiate distance runners to non-running undergraduate student controls	An American controlled cross-sectional study by Ryujin et al. (1999) used 55 female participants to compare eating disorder symptomology in collegiate distance runners (n=20) to non-running undergraduate student controls (n=35) using the Eating Disorders Inventory 2 as measurement. Differences were significant in the following subscales: Drive for Thinness (t(107) = 3.34, p < .005), Bulimia (t(107) = 2.48, p < .05) and Body Dissatisfaction (t(107) = 4.23, p < .001). Significance was approached for Interpersonal Distrust (t(107) = 1.70, p < .10) and Impulse Regulation (t(107) = 1.65, p = .10). Results found that distance runners showed no enhanced symptomatology of eating disorders, instead the female distance runners exhibited fewer symptoms of eating disorders on all subscales of the EDI-2 except Perfectionism[33].
**34**	Leedy (2000)	Comparing depression and anxiety in runners to non-runners	An American controlled cross sectional study by Leedy (2000) used 276 participants to compare depression and anxiety in runners with an average of 11.5 years of running experience (n=239, 56.1% men, mean age 37.9) to non-runners (n=37, 62% women, mean age 40.5) using an author created questionnaire designed to measure anxiety and depression based on the Diagnostic and Statistical Manual -IV, and an author adapted scaled based on the Running Addiction Scale as measurement. 16.2% of non-runners and 4.6% of runners indicated that they had been diagnosed with an anxiety disorder or prescribed an anxiolytic medication at some point in their life. These participants had significantly higher anxiety trait scores than those without a diagnosis, $F_{(1,274)}$= 18.87, p<.0001. 27% of non-runners and 11.8% of runners reported a diagnosis of depression or being prescribed an antidepressant. These participants had significantly higher measures of depression traits: $F_{(1,274)}$=22.46, p<.0001. Runners who were classified as highly committed (n=31) had significantly lower anxiety ($F_{(2,113)}$= 5.73,

			p<.01) and depression scores ($F_{(2,113)}$= 8.00, p<.001) than those classified as recreational runners (n=46) and non-runners (n=39). Women's Stress Relief scores were significantly higher than the men's ($F_{(1,229)}$= 20.51, p<.001). Stress relief scores also varied across race length, $F_{(2,229)}$ = 6.47, p<.005, indicating that the runners entered in the 5K - 10K runs had lower scores than those running the half or full marathon. Overall, the strongest motivator for running was Health/Fitness, ($F_{(2,229)}$=135.3, p<.001), for both men and women, and for all three race distances. The Committed Runners had significantly higher scores across motivation factor scores compared to the Recreational Runners, ($F_{(1,156)}$= 7.00, p<.01), with again, the most strongly endorsed motivation factor being Health/Fitness ($F_{(2,156)}$=39.13, p<.001). Overall, results indicate that highly committed runners had significantly lower anxiety and depression than recreational runners and non-runners[34].
**35**	Edwards (2005)	Comparing psychological wellbeing and physical self-perception in hockey players, runners and health club gym members vs a control group of non-exercisers	A South African cross sectional study by Edwards et al. (2005) used 277 participants (183 women and 94 men) with a mean age of 25.2 to compare psychological wellbeing and physical self-perception in regular exercisers including hockey players (n=60), runners (n=40) and health club gym members (n=69) vs a control group of non-exercisers (n=108), using Ryff's Short Standardized 18 item scale of Objective Psychological Wellbeing and Fox's Physical Self-Perception Profile (PSPP) and the Physical Self-Perception Profile as measurements. Regular exercisers scored significantly higher (p<0.01) than controls on 11 out of the 15 dimensions of psychological-well-being and physical self-perception: autonomy (F=11.3), personal growth (F=35.4), environmental mastery (F=9.6), purpose in life (F=149.2), positive relations with others (F=81.6), self-acceptance (F=50.4), sport competence (F=41.3), conditioning (F=28.1), sport importance (F=11.7), conditioning importance (F=28.1), body importance (F=31.0). Runners scored significantly higher than controls on autonomy, personal growth, environmental mastery, purpose in life, positive relations, self-acceptance, sport competence, conditioning, sport importance and conditioning importance. However, results don't give details of significance. Runners had the lowest physical self-worth average score out all groups. Hockey players reported more positive relations with others and sport competence compared to health club members or runners, but no report of significance for either. Men scored higher on sport (F=27.2, p<0.01), conditioning (F=20.1, p<0.01), body (F=13.3, p<0.01), sport importance (F=7.2, p<0.01) and conditioning importance (F=6.3, p<0.01). No significant influences of age or language, but gender was related to body attractiveness (F=13.5, p<0.01). Overall results show that all three forms of physical activity were associated with higher scores on the psychological well-being and physical self-perception scales compared to the control group[35].
**36**	Schnohr (2005)	Comparing stress levels between jogging and various levels of physical (in)/activity in leisure time	A large Danish observational cohort study by Schnohr et al. (2005) used 12,028 participants (5479 men and 6549 women) aged 20-79 to compare stress levels between jogging and various levels of physical (in)/activity in leisure time using an author created questionnaire as measurement. In both males and females, those who were vigorously physically active (joggers) had the lowest level of stress compared to those with low activity levels (males 3.1% vs 12.8%, respectively; females= 3.3% vs 19.3%, respectively). With increasing physical activity in leisure time, there was a decrease in high level of stress, between sedentary persons and joggers (OR= 0.30). With increasing physical activity there was also a decrease in life dissatisfaction, between sedentary persons and joggers (OR= 0.30). Highest levels of stress and dissatisfaction was seen in the sedentary persons who remained inactive at follow-up. In contrast, the group that changed from sedentary to active had an adjusted OR of <0.50. The physically active who remained active through follow-up reported the lowest level of both stress and dissatisfaction. Associations between physical activity and stress/life dissatisfaction were similar in men and women, showing that if either gender changed from sedentary to more physical activity in leisure time, the was decreased stress and lower life dissatisfaction. In contrast if they become sedentary, the opposite is true. Overall results showed a clear trend of higher level of stress and of life dissatisfaction in the sedentary group compared with the more active running groups[36].
**37**	Strachan (2005)	Investigating the relationship between running and self-efficacy and self-identity	A Canadian prospective longitudinal study by Strachan et al. (2005) used 67 regular runners with an average age of 40.6 (52% were female and had been running on average for 8.69 years) to investigate the relationship between running and self-efficacy and self-identity using author created measures of task self-efficacy, self-regulatory efficacy and a 10-item, validated athletic identity measurement scale. Participants filled out a questionnaire and four weeks following this initial assessment, were contacted over the phone in order to obtain a measure of their running behaviour over the last week. There was significant comparisons between extreme self-identity groups (high vs low) on social cognitive and behavioural variables ($F_{(5,37)}$=4.72, p<.002). Results found that those higher in self-identity showed significantly higher scores on

			task self-efficacy (p<.001), scheduling self-efficacy (p<.03), ran more frequently (p<.001) and for longer durations (p<.005), than those who scored lowest on self-identity. Both scheduling self-efficacy (R^2change =.16, p< .001) and barriers self-efficacy (R^2change = .22, p<.001), were significantly correlated with self-identity to prospectively predict running frequency ($F_{(2,64)}$ =9.98, p< .001; $F_{(2, 63)}$ = 12.89, p<.001, respectively). Both task self-efficacy (R^2change=.06, p< .05) and self-identity (R^2change =.06, p< .04) were significant predictors of maintenance duration. Overall both types of self-regulatory efficacy were related to prospectively were predictive of maintenance running frequency[37].
**38**	Galper (2006)	Assessing retrospectively if level of walking/running impacted depression and emotional wellbeing	An American retrospective cross sectional study by Galper et al. (2006) used 6728 participants (5451 men with a mean age of 49.5, and 1277 women with a mean age of 48.1) to assess retrospectively if level of walking/running impacted depression and emotional wellbeing using the Center for Epidemiological Studies Scale for Depression and the General Well-Being Schedule as measurements.<br>The participants were classified into four categories: inactive (walking/jogging/running <1 mile per week); insufficiently active (1–10 miles per week); sufficiently active (11–19 miles per week); (highly active (>20 miles per week). 27% (n=1454) of the men were classified as inactive, 35% (n=1892) as insufficiently active, 26% (n=1396) as sufficiently active, and 13% (n=709) as highly active. Likewise, 33% (n=422) of the women were classified as inactive, 35% (n=443) as insufficiently active, 22% (n=283) as sufficiently active, and 10% (n=129) as highly active. Results found that among men and women in the study, relative increases in habitual physical activity are cross-sectionally associated with significantly lower depressive symptomatology (P < 0.0001) and greater emotional well-being (P< 0.0001). This peaked at 11–19 miles per week. There was an inverse association between physical activity and estimated mean depression scores for both men ($F_{(6, 5306)}$=20.93, P<0.0001) and women ($F_{(6, 1247)}$ = 11.80, P< 0.0001). Inactive men had greater depressive symptom severity than insufficiently active men (P < 0.0001) and highly active men (P<0.0001). Inactive women had greater depressive symptom severity than insufficiently active women (P<0.0001), sufficiently active women (P< 0.0001) and highly active women (P<0.0001). ANCOVA demonstrated a positive association between physical activity and estimated mean wellbeing scores in men ($F_{(6, 5306)}$ = 78.65, P<0.0001) and women ($F_{(6, 1247)}$ = 24.82, P<0.0001). Inactive men had lower emotional wellbeing than insufficiently active men (P<0.0001), sufficiently active men (P<0.0001), highly active men (P<0.0001). Inactive women had lower emotional well-being than insufficiently active women (P<0.0001), sufficiently active women (P <0.0001), and highly active women (P<0.0001). Overall results suggest that increased habitual physical activity reduces depression and increases emotional wellbeing[38].
**39**	Luszcynska (2007)	Investigate the relationship between self-efficacy and running behaviour	A UK based longitudinal prospective cohort study by Luszcynska et al. (2007) used 139 runners (111 men and 29 women) with a mean age of 29.5 to investigate the relationship between self-efficacy and running behaviour using an author created questionnaire as measurement to collect data twice with a time gap of 2 years. Participants were divided into subgroups with strong (n=72) and weak (n=66) maintenance self-efficacy, into strong (n=72) and weak (n= 61) recovery self-efficacy, and into strong (n=87) and weak (n=45) intentions. Participants reduced the number of running or jogging sessions over the 2 years, regardless of their strong, or weak intentions at baseline ($F_{(1,130)}$=34.55, p<.001). Again, participants declined in frequency of running/jogging over 2 years, regardless of their strong or weak baseline maintenance self-efficacy ($F_{(1,130)}$= 42.12, p<.001). Overall, all participants reduced the number of jogging or running sessions over two years ($F_{(1,131)}$=43.43, p<.001), however, those with strong baseline recovery self-efficacy ran/jogged more often at 2 year measurement than those who had weak recovery self-efficacy at baseline ($F_{(1,131)}$=6.12, p<.05). Recovery self-efficacy and intention jointly predicted running/jogging behaviour 2 years later ([$F_{(1,131)}$= 43.43, p<.001] and [$F_{(1,130)}$ =34.55, p<.001], respectively), whereas running/jogging behaviour did not predict recovery self-efficacy and intention. No effects of maintenance self-efficacy were found. Recovery self-efficacy at T1 predicted recovery self-efficacy (p<.05), maintenance self-efficacy (p<.05) and jogging or running behaviour (p<.05) assessed 2yr later. Overall, social–cognitive variables predicted behaviour, whereas behaviour did not predict social–cognitive variables. The majority of participants (n =120) experienced at least one 2-week period of decline in running or jogging behaviour. Among those who experienced lapses, recovery self-efficacy remained the only significant social-cognitive predictor of behaviour, accounting for 30% of the variance of behaviour measured 2 years later (*B*=.19, p<.05). Overall, results found that participants decreased the frequency of running sessions after 2 years, regardless of baseline intensions or self-efficacy, however those with stronger recovery self-efficacy jogged more than those with weaker recovery self-efficacy 2 years later[39].

**40**	Smith (2010)	Comparing exercise dependence, running addiction and social physique anxiety in male vs female runners	A UK based cross sectional questionnaire by Smith et al. (2010) used 93 non-competitive, regular runners with a mean age of 28.05 to compare exercise dependence, running addiction and social physique anxiety in male (n=47) vs female (n=46) runners using the Exercise Dependence Scale, Running Addiction Scale and Social Physique Anxiety Scale as measurements. Results found that a significant proportion of runners displayed symptoms of exercise dependence, however there were no significant differences were found between the males and females (p>.05 in all cases). There was no significant difference between males and females for running addiction scale (22.64 and 20.91, respectively), social physique anxiety scale (22.30 and 22.61, respectively) or total exercise dependence scale scores (72.56 and 66.86, respectively). Overall results did not find that exercise dependence was linked to social physique anxiety ($F_{(3.179)} = 1.21$, p>.05), nor that there was a difference between men and women[40].
**41**	Gapin (2011)	Comparing disordered eating in obligatory and non-obligatory runners	An American cross sectional study by Gapin et al. (2011) used 179 regular runners (88 male and 91 female) with a mean age of 36.0 to compare disordered eating in obligatory (91) and non- obligatory runners (n=82) using the Eating Disorder Inventory (EDI), Athletic Identity Measurement Scale (AIMS) and Obligatory Exercise Questionnaire (OEQ) as measurements. Obligatory runners scored significantly higher (P<0.002) on all of the EDI eating attitudes/disorder measures: (Obligatory mean = 8.07, non-obligatory mean =4.42), F(1,166)=9.75, P=0.002; Drive for Thinness: (Obligatory mean = 6.42, non-obligatory mean =3.01), F(1,166) =28.91, P<0.001; Perfectionism: (Obligatory mean = 6.77, non-obligatory mean =3.73), F(1,166) = 21.59, P<0.001; Bulimia (Obligatory mean =1.37, non-obligatory mean =0.17), F(1,166) =10.43, P= 0.001. Obligatory runners also scored significantly higher on the AIMS (Hotelling's T2= 0.440, F(8,161) = 8.85, P<0.001). Results from the OEQ indicated that runners in the obligatory group demonstrated greater concern with dieting, preoccupation with weight, and pursuit of thinness. Overall the findings suggest that obligated running (exercising to maintain identification with the running role) may be associated with pathological eating and training practices[41].
**42**	Wadas (2014)	Investigating any relationship between male runners with disordered eating behaviours and eating attitudes	An American cross sectional study by Wadas (2014) used 68 male high school cross country runners with a mean age of 15.9 (70.6% white race) to investigate any relationship between male runners with disordered eating behaviours and eating attitudes using a questionnaire consisting of The Exercise Motivation Inventory 2, the Eating Attitudes Test 26 and the ATHLETE questionnaire as measurements. Factors that had a significant relationship with disordered eating are weight management (r =.31, p =.011), drive for thinness and performance (r = 0.36: p < 0.05), and the Feelings about Performance subscale (or Performance Perfectionism) (r = 0.26: p < 0.05). No significant relationships were found between disordered eating behaviors in male cross country athletes and personal body feelings (r =.19, p = .109), feelings about eating (r =.18, p =.137), and feelings about being an athlete (r =.12, p =.345). The mean EAT-26 score for all participants was 6.0, with 4.41% (n=3) male high school cross country runners scoring 20 or higher on the EAT-26, indicating at risk for disordered eating and displays symptoms. An additional 13.2% (n = 9) met the cut-off score of 14 for disordered eating behaviours, one standard deviation above the mean for population norms. Overall results found that risk factors associated with eating disorders existed within high school male cross country runners[42].
**43**	Samson (2015)	Investigating the relationship between self-esteem and psychological coping with marathon running	An American cross sectional questionnaire by Samson et al. (2015) used 308 marathon runners (117 males and 191 females) with a mean age of 41 to investigate the relationship between self-esteem and psychological coping with marathon running, using the Motivation for Marathons Scale, The Perceived control questionnaire and The Sport Mental Toughness Questionnaire as measurements. Self-esteem was positively associated with perceived control (r=.40) (x^2_7= 47.08, p<.001, CFI=.85; RMSEA=.14), but negatively associated with mental toughness. There was also a positive relationship between perceived control and psychological coping (r=.42) (x^2_8 =45.65, p<.001; CFI=.85; RMSEA=.12), suggesting that runners who ran for those reasons also reported higher levels of perceived control regarding the outcome of the race, however, it was not directly related to perceptions of mental toughness. The mean MOMS scores for psychological coping and self-esteem suggested than females were more likely to run for these reasons than males: 4.8 & 4.42 respectively for psychological coping, and 5.22 & 4.62 for self-esteem. Overall results suggest that females were motivated to run to improve self-esteem and psychological coping than men[43].
**44**	Lucidi (2016)	Investigating the relationship between running and stress in	An Italian cross sectional prospective field study by Lucidi et al. (2016) used 669 runners training for a marathon (85% male) with a mean age of 42.07 to investigate the relationship between running and stress using the Perceived Stress Scale, the Passion Scale and The Italian version of the Locomotion and Assessment Scales as measurements. Runners filled out the survey 15 days prior to the marathon to evaluate

		runners training for a marathon	stress. Running positively predicted harmonious passion (β = 0.37; P < 0.001), which in turn reduced athletes' experience of stress, whereas assessment positively predicted obsessive passion (β = 0.26; P < 0.001). Harmonious passion negatively predicted athletes' experience of anticipatory stress (β = −0.28; P< 0.001), whereas obsessive passion positively predicted it (β = 0.45; P< 0.001). These effects were estimated controlling for athletes' training frequency, which was not significantly related to athletes' stress. The indirect effect of running on anticipatory stress perception through harmonious passion was statistically significant (αβ = −0.10; 95% confidence interval: from −0.15 to −0.05). Similarly, the indirect effect of assessment on stress through obsessive passion was statistically significant (αβ = 0.12; 95% confidence interval: from 0.07 to 0.17). Results also indicated a significant direct effect of assessment on the athletes' experience of stress (β = 0.22; P < 0.001). The direct effect of running on stress was not significant (β = −0.01; P = 0.75). Overall results suggest that running does not directly impact stress, however running increases harmonious passion which improves stress[44].
**45**	Batmyagmar (2019)	Comparing self-reported health & wellbeing & quality of life over 4 years in elderly marathon runners to non-exercising controls	An Austrian prospective longitudinal study by Batmyagmar et al. (2019) used 99 participants to compare self-reported health and wellbeing and quality of life over 4 years in elderly marathon runners (n=50, mean age of 66, 46 men and 4 women) to non-exercising controls (n=49, mean age of 66, 44 men and 5 women) using the Short Form Health Survey (SF-36) as measurement. SF-35 scores in all domains remained stable over time and, in nearly all of them, marathon runners showed higher self-reported health than non-athlete controls. Athletes evaluated their health as better than non-athletes in the following categories: general health perceptions (mean control= 81 vs athletes=81; between subjects F= 14.21, p<0.001); vitality (mean control= 69 vs athlete= 80; between subjects F= 13.37, p<0.001); social functioning (mean control= 87 vs athlete=97; between subjects F= 11.30, p<0.001); emotional role functioning (mean control= 84 vs athlete=98; between subjects F=1.42, p<0.002); mental health (mean control= 78 vs athletes=84; between subjects F=6.07, p<0.0016). Overall, findings suggest that extensive high intensity endurance exercise is associated with improved subjective health and wellbeing in elderly persons[45].
**46**	Cleland (2019)	Investigating enjoyment, self-efficacy and factors of participation in Parkrun event participants	An Australian cross sectional study by Cleland et al. (2019) used 372 participants of 'Parkrun' events with a mean age of 43.8 to investigate enjoyment, self-efficacy and factors of participation using author-created questionnaires to assess psychological/cognitive measures, social support and environmental level factors. These Parkrun subjects were divided into three groups: regular walker/runner (n=175, 55% female, mean age 45.0), occasional walker/runner (n=142, 57.8% female, mean age 42.5) and non-walker/runner (n=54, 68.5% female, mean age 43.3). Results were often reduced when adjusted for length of time since registration, ie. absolute parkrun participation (total number of parkrun events) compared to adjusted parkrun participation (absolute park-run participation adjusted for the number of weeks registered). Perceived benefits of parkrun including enjoyment (absolute participation: *B* coefficients= 0.32; and adjusted participation: *B* coefficients = 0.22) and social factors (absolute: *B*= 0.70; and adjusted: *B*= 0.35) were positively associated with participation as was overall enjoyment (absolute: *B*=0.30; and adjusted: *B*= 0.30), self-efficacy for parkrun (absolute: *B*=0.46; and adjusted: *B*= 0.33), social support for parkrun from family (absolute: *B* =0.05; and adjusted: *B* =0.03) and social support from friends (absolute: *B*= 0.04; and adjusted: *B*= 0.02) related to parkrun. Perceived social benefits (absolute: *B*= 0.43; and adjusted: *B*= 0.17) and self-efficacy for parkrun (absolute: *B*= 0.18; and adjusted: *B*= 0.13) were positively associated with absolute and adjusted parkrun participation. Overall results suggested that higher participation levels of park-run events correlated with greater self-efficacy and perceived social benefits[46].
**47**	Lukacs (2019)	Investigating the prevalence of exercise addiction and psychological features in amateur runners, including; perceived health, life satisfaction, loneliness, stress, anxiety, depression,	A Hungarian cross sectional questionnaire study by Lukacs et al. (2019) used 257 amateur runners (126 females and 131 males) with a mean age of 40.49 and at least 2 years of running experience. The study investigated the prevalence of exercise addiction and psychological features including; perceived health, life satisfaction, loneliness, stress, anxiety, depression, body shape and eating disorders; using the Exercise Dependence Scale, a Cantril ladder for Overall life satisfaction, SCOFF eating disorder questionnaire, the UCLA 3-item Loneliness Scale, Body Image Subscale from the Body Investment scale and the 'Depression, Anxiety and Stress Scale-21'. About 53.6% (n=137) of respondents were characterized as non-dependent symptomatic, 37.8% (n=97) as non-dependent asymptomatic and 8.6% (n=23) were at risk of exercise addiction. The logistic regression model indicated that five variables significantly predicted the risk of exercise addiction: weekly time spent running [B=1.42, 95% CI for odds ratio=4.17, p<.001], childhood physical activity [B=2.06, 95% CI for odds ratio=7.86, p=.008], lower educational attainment [B=1.97, 95% CI for odds ratio=7.17, p=.006], anxiety [B=0.47, 95% CI for odds ratio=1.61, p=.023], and loneliness [B=0.79,

		body shape and eating disorders	95% CI for odds ratio=2.21, p=.004]). Subscale results of the exercise dependence scale suggested that to deal with both anxiety and loneliness, as runners from all groups found it important to spend a significant amount of time engaging in exercise [Time subscale (3.09, SD = 1.11, 95% CI = 2.96–3.23]) and continually increase exercise intensity, frequency, and duration [Tolerance subscale (3.71, SD = 1.28, 95% CI = 3.55–3.87)] to achieve joyfulness and happiness. The at risk group for exercise addiction scored higher on the Lack of Control subscale (4.90, SD = 0.76, 95% CI = 4.57–5.23) and therefore these runners were less able to control the urge to exercise or to stop exercising for a significant time. All investigated groups showed fewer problems on the Intention subscale (exercising longer than intended, expected, or planned; (2.39, SD = 1.10, 95% CI = 2.25–2.52) and the Reduction in Other Activities subscale (choosing or thinking about exercise rather than spending time with family, friends, or concentrating on school or work; (1.90, SD = 0.82, 95% CI = 1.80–2.00). ANOVA post hoc test results showed that all three groups significantly differed from each other in all subscales (all $p<.001$): tolerance ($F=63.053$, $n_p^2 = .365$), Time ($F=68.147$, $n_p^2 = .371$), Continuance ($F=41.578$, $n_p^2=.304$), Lack of control ($F= 171.509$, $n_p^2 = .587$), Withdrawal ($F=32.757$, $n_p^2 = .222$), Intention Effect ($F= 61.963$, $n_p^2 = .360$), Reduction ($F=65.249$, $n_p^2 = .386$). The study results did not comment on the other psychological features (perceived health, life satisfaction, stress, depression, body shape and eating disorders)[47].

Supplementary Table S2

Narrative description of findings of the 23 studies with a single bout of running.

	Author		Narrative description of findings
1	Nowlis (1979)	Impact of a 12.5 mile jog on mood and anxiety	A Canadian pre-post non-controlled study by Nowlis et al. (1979) used 18 experienced joggers (5 females and 13 males) who ranged in age from 17 to 55, to investigate how a 12.5 mile jog impacted mood and anxiety using the Mood Adjective Checklist and State Trait Anxiety Inventory as measurements. Following the 12.5 mile run there was significant improvement from pre- to post- measures of pleasantness (2.00 to 2.67;$p< 0.01$) and a significant decrease in Trait anxiety (34.81 to 33.31; $p<0.10$). There was an increase in activation, a reduction in state-anxiety, and a reduction of sadness, anxiety, depression and relaxation subscales… but no significance was reached in any of these[48].
2	Wilson (1981)	Impact of a solo indoor track run on anxiety	A Canadian pre-post controlled study by Wilson et al. (1981) used 42 participants consisting of 20 runners, 12 participants of a 40 minute aerobic exercise class and 10 lunchers, all aged between 21 and 28 (23 women and 19 men) to compare the impact of solo indoor track running (n=20), an aerobics class (n=12) and lunching (n=10) on anxiety using the State-Trait Anxiety Inventory as measurement. Each group showed significant decreases in anxiety after the activity ($F_{(1,39)}$=15.63, $p<0.003$) but no differences between groups ($F_{2,39}$= 1.27, $p > 0.05$) and no interaction ($F_{2,39}$ = 1.57, $p > 0.005$) were observed. Results suggest that frequency of runs per week is the most important factor for decrements in anxiety during running sessions ($r = -0.58$, $p < 0.01$) and that initial level of anxiety was positively related to decreased anxiety following activity ($r = .63$, $p < .005$) for both men and women[49].
3	Markoff (1982)	Impact of a 1 hour run on mood	A Hawaiian pre-post non-controlled study by Markoff et al. (1982) used 15 participants (11 men & 4 women) aged 23-45 who had all ran at least 1 marathon to examine the impact of 1 hour of running on mood using the Profile of Mood States as measurement. There was a significant reduction of anxiety pre- to post-run (5.2 to 3.27 in men and 3.08 to 2.15 in women). The t-test for anxiety was 2.72 and thus $p<0.01$. For depression, there was a non-significant decrease in scores pre- to post- run (4.93 to 1.73 in men and 7.39 to 1.83 in women). The t-test for depression was 1.80 which was not significant[50].
4	Thaxton (1982)	Impact of 30 minutes outdoor running on mood	An American non-randomised controlled trial by Thaxton et al. (1982) used 33 regular runners with a mean age of 36 (24 males and 9 females) who were divided into 4 groups to compare pre-test 30 minute outdoor running test (n=6), pre-test no running test (n=9) no pre-test 30 minute running test (n=11) and no pre-test no running test (n=7) on mood using the Profile of Mood States as measurement. Significant differences in the depression scores between the running and abstaining (non-pretested) groups, $F(1,29) = 4.8$,$p<.05$, however no significant differences between anxiety, vigour, and fatigue scores[51].
5	McGowan (1991)	Impact of 75 minutes of jogging on an outdoor track on mood	An American non-randomised controlled trial by McGowan et al. (1991) used 72 college students to compare the effect of 75 minutes of jogging on an outdoor track (n=25) vs 75 minutes of karate (n=11), weight training (n=26) and a stationary science lecture class (n=10) on mood using the Profile of Mood States as measurement. The running group exhibited significant changes in total mood disturbance from pre-(35.68) to post (24.16) test… t_{24} = 2.84, $p<0.009$. The weight lifting group had changes of $F_{6,20}$ = 2.60, $p = 0.05$, but there were no significant changes observed for the karate group or control[52].
6	Goode (1993)	Impact of own training run on mood	An American pre-post non-controlled study by Goode et al. (1993) used 150 regular runners with a mean age of 31.7 (69% male, 31% female) to investigate mood states before and after subjects own training run using the Profile of Mood States as measurement. All but one (vigor) of the POMS scales showed significant ($p<0.1$) changes following the run. Tension/anxiety (mean change of -3.1, $p<0.1$), depression (mean change of -1.5, $p<0.1$), confusion (mean change of -1.1, $p<0.1$) and anger mean change of -1.8, $p<0.1$) all reduced significantly post run, while fatigue significantly increased post run (mean change of +1.8, $p<0.1$)[53].
7	Morris (1994)	Impact of a 3 mile 'fun-run' on mood	A British pre-post non-controlled study by Morris et al. (1994) used 165 members of a road runners club (98 males and 67 females) with a mean age of 34 to examine how a 3 mile 'fun-run' impacted mood using an author devised adjective checklist based on POMS as measurement. Positive mood was increased after running ($F(_{1,163})$=68.18, $p<0.001$), negative mood decreased after running ($F_{(1.163)}$ = 47.62, $p<0.001$) and improvements in mood were greater in women than men but was not significant ($p>0.1$)[54].

**8**	Rudolph (1996)	Impact of various timings of treadmill running on self-efficacy (10, 15 and 20 minutes)	An American randomised non-controlled trial by Rudolph et al. (1996) used 36 moderately-active female university students with a mean age of 20.6 to compare the impact of 10 (n=12), 15 (n=12) and 20 (n=12) minutes of treadmill running on self-efficacy using the Exercise-Efficacy Scale as measurement. Mean scores of self-efficacy increased significantly in all 3 groups, from pre to post exercise ($F_{(1, 33)}$= 74.57, p< .001): the 10 minute (43.2 to 55.6), 15 minute (34.7 to 45.6) and 20 minute (37.3 to 53.4) exercise conditions. The within-group effect sizes for self-efficacy were calculated. Although the largest effect size (ES) occurred in the 20 minute condition (ES= .68), the effect sizes in the 15 (ES= .36) and 10 (ES= .49) minute conditions are also moderate in magnitude[55].
**9**	Cox (2001)	Impact of 30 minutes of treadmill jogging at either 50% or 75% predicted VO_2 max on psychological affect and wellbeing	An American randomised controlled trial by Cox et al. (2001) used 24 physically active male university students with a mean age of 28.3 to compare the impact of 30 minutes of treadmill jogging at either 50% or 75% predicted VO_2 max vs a stationary stepper on psychological affect and wellbeing using the Subjective Exercise Experiences Scale. Results showed that following an acute bout of aerobic exercise a significant linear trend for time was observed for psychological distress (p=0.001, η^2_p=0.17) and a significant linear trend for time for positive well-being (p=0.037, η^2_p =0.07) but there was no significant difference between the wellbeing for the stepper vs the treadmill running group[56].
**10**	O'Halloran (2002)	Impact of a 60 minute treadmill run on mood	An Australian pre-post non-controlled study by O'Halloran et al. (2002) used 50 regular runners (25 men and 25 women) with a mean age of 26.6 to examine how a 60 minute treadmill run impacted mood using the Profile of Mood States and Beliefs Concerning Mood Improvements Associated With Running Scale as measurements. The pre vs post exercise scores from POMS was as follows: Agreeable-Hostile = 27.3 to 27.58; Composed-Anxious = 25.6 to 29.12; Clearheaded-Confused = 27.20 to 28.62; Confident-Unsure = 25.22 to 25.10; Elated-Depressed = 24.56 to 27.10; Energetic-Tired = 22.42 to 23.48. There were significant reductions in anxiety (p<0.05), depression (p<0.01) and confusion (p<0.05, however there was not a significant change of confidence. The largest correlation (r =.44) was between the beliefs scale and changes on the Elated-Depressed subscale (p<0.01)[57].
**11**	Szabo (2003)	Impact of 20 minutes of track running on anxiety and feelings	A UK based pre-post non-controlled time series quasi-experimental study by Szabo et al. (2003) used 39 sports science university students (22 male and 17 female) aged between 20 and 23 who all had a British-Caucasian cultural background to compare the impact of 20 minutes of track running, a comedy video and a nature documentary on anxiety, positive wellbeing and psychological distress using the Spielberger State Anxiety Inventory and Exercise induced Feeling Inventory as measurements. Both exercise and humour had an equally positive effect on psychological distress and positive wellbeing. State anxiety significantly reduced with exercise ($F_{(1.5, 58.3)}$ = 5.32, p<0.01), however, exercise had a statistically lower reduction on anxiety than humour ($t_{(38)}$ = 3.36, p<0.002)[58].
**12**	O'Halloran (2004)	Impact of a 60 minute treadmill run on mood	An Australian randomised controlled study by O'Halloran et al. (2004) used 160 regular runners (80 males and 80 females) between age 18 and 40 to compare how a 60 minute treadmill run (n=80) vs no running (n=80) impacted mood using the Profile of Mood States and Beliefs Concerning Mood Improvements Associated With Running Scale as measurements. There were improvements in composure, energy, elation and mental clarity during the run relative to the control condition and pre-exercise assessment. Other than the Energetic-tired subscale where improvements were evident at 25 minutes ($F_{(1,156)}$ =10.09, p=.002), most subscales didn't have mood improvements until the 40 minute assessment during the run. Runners became more composed (less anxious) $F_{(1,156)}$ =9.47, p=.002), more clear headed (less confused) ($F_{(1,156)}$ =5.57, p=.02) and more elated (less depressed) ($F_{(1,156)}$ =10.18, p=0.002) by 40 minutes into a 60 minute treadmill run. Although there was a trend for differences on the beliefs concerning mood improvements after running scale, mean scores on the beliefs concerning mood during running scale were strikingly similar for the running (3.69) and the control groups (3.67)[59].
**13**	Robbins (2004)	Impact of 20 minute treadmill run on self-efficacy in children and adolescents	An American pre-post non-controlled study by Robbins et al. (2004) used 168 inactive African American and European American children & adolescents with a mean age of 12.6 (49% female) to investigate how 20 minutes of treadmill exercise impacted self-esteem using the Walking Efficacy Scale as measurement. There was an increase in self-efficacy post-exercise $F_{(1, 158)}$ = 84.31, p < .001, however girls reported significantly lower pre-activity self-efficacy (M = 41.02, SD = 24.37) than boys (M = 52.46, SD= 23.99) with $t_{(166)}$ = 3.07, p < .01, and subsequently reported greater perceived exertion. African American girls reported significantly lower pre-activity self-efficacy than the other three race-gender groups $F_{(3,164)}$ = 5.55, p < .01[60].

**14**	Pretty (2005)	Impact of a 20 minute treadmill run with rural vs urban stimuli on mood and self-esteem	A UK based randomised controlled trial by Pretty et al. (2005) used 100 participants (55 female and 45 male) with a mean age of 24.6 to investigate how pleasant and unpleasant urban vs rural stimuli whilst running on a treadmill for 20 minutes impacted mood and self-esteem, using the Profile of Mood States and Rosenberg Self-Esteem Questionnaire as measurements. There were 20 participants in each of the 5 different stimuli groups: rural pleasant, rural unpleasant, urban pleasant, urban unpleasant and the control group who exercised with blank white screens. There was a significant increase in self-esteem (from 19.4 (+/-0.4) to 18.1 (+/-0.4), p < 0.001) following exercise, however both rural and urban pleasant scenes produced a significantly greater positive effect on self-esteem than exercise alone, while both rural and urban unpleasant scenes reduced the positive effects of exercise on self-esteem. There were significant reductions in confusion, p < 0.01; and tension-anxiety, p < 0.001, while a significant improvement vigour, p < 0.001) following exercise[61].
**15**	Hoffman (2008)	Impact of a 30 minute treadmill run on mood	An American pre-post pre-experimental study by Hoffman et al. (2008) used POMS to examine how a 30 minute treadmill run altered mood using 32 subjects (16 regular exercisers and 16 non exercisers, consisting of 8 women and 8 men in each group). Post exercise total mood disturbance was decreased 16 +/- 10 (95% CI, 7–24) among the moderate exercisers, and 9+/- 13 points (95% CI, 1–18) among the non-exercisers. TMD improves post-exercise in both the exercisers and non-exercisers, but the exercisers experience almost double the effect. A "nearly significant group-by-time interaction" (P.08) is suggestive of a trend toward less of an effect among the non-exercisers than the other groups[62].
**16**	Kwan (2010)	Impact of a 30 minute treadmill run on general affective response	An American pre-post non-controlled study by Kwan et al. (2010) used 129 participants (67 women and 62 men, 80% white ethnicity) with a mean age of 22 to show the positive impact of a 30 minute treadmill run on general affective response using the Physical Activity Affect scale (PAAS) as measurement at 6 time points before, during and after the exercise. There was a positive effect during exercise (b = .52, SE = .09, p < .0001) and between baseline and 15 minutes post-exercise (b= .73, $CI_{.95}$ = .56, .89, t(126)= 8.63, p < .0001)[63].
**17**	Weinstein (2010)	Impact of 25 minutes of increasing graded treadmill running on mood and depression	An American pre-post controlled study by Weinstein et al. (2010) used 30 participants with a mean age of 39.8 (50% women); 14 of whom were diagnosed with minor (n=2) or major (n=12) depressive disorder and 16 of whom were controls; to examine how 25 minutes of increasing graded treadmill exercise impacts mood and depression using the Becks depression Inventory scale and Profile of Mood States as measurements. Immediately following exercise, depressed individuals displayed improvements in depressed mood from baseline (p=0.02) but subsequently exhibited increased depressed mood from baseline at 30 mins post exercise $F_{interaction(1,27)}$= 3.98; p=0.05; ηp^2 =0.13. The severity of depression (as assessed by BDI-II) was significantly related to increases in depressed mood (r = 0.60; p = 0.001) at 30 min post-exercise[64].
**18**	Anderson (2011)	Impact of a light 10 minute outdoor jog on mood	A British randomised controlled trial 2x2 mixed design by Anderson et al (2011) used 40 participants aged 18-25 from various sports clubs to compare the impact of a light 10 minute jog outside on a grass playing field vs a 10 minute cognitive task on mood using the 'Incredibly Short Profile of Mood States' as measurement. The between persons design found a significant mood enhancement (F(1,38) = 24.18, p <.001, n^2_p = .39) within the exercise group, compared with the non-exercise control group[65].
**19**	Kane (2013)	Impact of the running pacer challenge (20m sprints within increasing pace inside a gymnasium) on self-efficacy in children	An American pre-post non-controlled study by Kane et al. (2013) used 34 school children aged 11 to 14 (18 female and 16 male) to examine how the PACER challenge (20m sprints within increasing pace inside a gymnasium) effected self-efficacy using the self-efficacy questionnaire adapted for children. The study found a decrease in self-efficacy following participation in the PACER (mean score decreased from 2.7 to 2.3 following exercise, t=4.6, p<.001, large effect size of d = 0.79), however there was a positive correlation between PACER laps and pre- and post- measures of exercise self-efficacy (mean score increased from .58 to .70 following exercise)[66].
**20**	Szabo (2013)	Impact of a 5km self-paced run along a public running path on states of affect	A Hungarian pre-post non-controlled study by Szabo et al. (2013) used 50 recreational runners (37 males and 13 females) with a mean age of 29.02 to investigate how a 5km self-paced run on a public running path impacted states of affect using the Exercise Induced Feeling Inventory as measurement. Significant positive changes were seen in all 4 measures of affect following the run: revitalisation ($F_{(1,48)}$ = 145.93, p < .001, partial n^2 = .75 with an effect size of 2.0), positive engagement ($F_{(1,48)}$ = 97.11, p< .001, partial = n^2 =.67 with an effect size of 1.6), tranquillity ($F_{(1,48)}$ = 85.02, p < .001, partial n^2 = .64 with an effect size of 1.5) and exhaustion ($F_{(1,48)}$ = 32.25, p < .001, partial n^2 = .40 with an effect size of 1.0)[67].

**21**	McDowell (2016)	Impact of a 30 minute treadmill run on mood and anxiety	An Irish randomised controlled trial by McDowell et al. (2016) used 53 participants (27 males and 26 females) with a mean age of 21.2 to compare the effects of 30 minutes of vigorous treadmill running vs 30 minutes of seated quiet rest on mood and anxiety using the State-Trait Anxiety Inventory and Profile of Mood States as measurements. Compared with the control, 30 minutes of acute aerobic exercise significantly improved state anxiety (F1,92 = 12.52, $P < 0.001$), feelings of depression (F1,86 = 5.05, $P < 0.027$), and total mood disturbance F = 36.91, $P < 0.001$[68].
**22**	Rogerson (2016)	Impact of a 5km park run on psychological wellbeing	A British pre-post non-controlled mixed between-within study by Rogerson et al. (2016) used 331 Park Run attendees (180 males and 151 females) with a mean age of 40.8 to investigate how a 5km Park Run impacted affective outcomes of psychological wellbeing using a questionnaire containing parts of the Profile of Mood States, Rosenberg Self-esteem scale and Perceived Stress Scale as measurement. There were significant ($p<.001$) improvements from pre- to post-run for self-esteem (7.7% improvement; $F_{(1, 324)} = 100.58$, $\eta^2 = .24$), stress (18.4% improvement; $F_{(1, 315)} =50.78$, $\eta^2{}_p = .139$) and total mood disturbance (14.2% improvement; $F(_{1, 278)} =22.15$, $\eta^2{}_p = .07$)[69].
**23**	Edwards (2017)	Impact of a 15 minute treadmill jog on stress and anxiety	An American randomised controlled trial by Edwards et al. (2017) used 27 participants aged between 18 and 35 to compare the effects of a 15 minute treadmill jog (n = 8) to the equivalent amount of time walking (n = 9) or stretching (n=10) on stress and anxiety after exposure to a film clip which was intended to elicit a negative emotional response using the Exercise Induced Feeling Inventory and Affective Circumplex Scale and the Strait-Trait Anxiety Inventory as measurements. It found a protective emotional effect from jogging, with reduced anxiousness (28.8 vs 13.1, $p = 0.06$) and stress (11.3 vs 9.4, $p = 0.11$) within the runners after being shown the emotive film. When comparing anxiousness scores from baseline to post-film clip, the p-values for the stretching, walking, and jogging groups were .21, .21, and .06, respectively, suggesting that anxiousness was more significantly different between baseline and post-film clip in the jogging group versus the walking or stretching groups. Unlike the walking ($p = .11$) and jogging (11.3 to 9.4, $p = .19$) groups, only the stretching group (1.2 to 26.0, $p = .048$) had an increased anger score from baseline to post-film clip[70].

Supplementary Table S3

Narrative description of findings of the 9 studies with a double bout of running.

	**Author**		**Narrative description of findings**
**1**	Krotee (1980)	Impact of 50m group vs solo run on anxiety	A pre-post pre-experimental non-controlled design by Krotee (1980) in the USA used 78 children (31 females and 47 males) between the ages of 7 and 12 to compare how a 50 metre run in individual vs small group settings impacted anxiety using the State-Trait Inventory for Children as measurement. In the individual setting, the level of pre initial (31.56) to pre termination (31.07) anxiety levels decreased while in the small group setting a slight but not significant gain in pre initial (30.54)to pre termination (31.40) anxiety level was realised. In the individual setting, the level of post initial anxiety (31.56) was higher at post termination (32.72)> This also happened in the small group setting, the level of post initial anxiety (30.67) increased at post termination level (31.83). There was Significant pre to pre (individual r = .9050 and small group r = .8667) and post to post (individual r=.8684 and small group r=.7335) correlations concerning the A-State level exists at the 0.001 level of confidence. It appears that there is relative stability between the various measures of pre and post A-State anxiety level and perhaps the physical activity and sport situational setting does not create as much anxiety for the participant as popularly purported. Results indicate that the children did not significantly increase in anxiety level (A-STATE) when participating in various physical activity and sport situational settings (ie. individual or group), however females) exhibited a significantly higher competitive anxiety level (A-TRAIT) than males prior to participation in the physical activity and sport situational setting (20.90 & 18.40, respectively)[71].
**2**	Wildmann (1986)	Impact of 2 identical 10km runs (1 week apart) on feelings of pleasantness & change of mood	A German based pre-post non-controlled study by Wildmann et al. (1986) used 21 male long-distance runners with a mean age of 29.8 to investigate how two 10km runs (1 week apart) under equal conditions on a 400m running track impacted 'feelings of pleasantness' and 'changes of mood' using the Eigenschaftsworterliste scale (an adjective check list) as measurement. Following running bouts there was a change in mood with good mood scoring higher after running. The mood elevation had considerable individual variability but there was a significant correlation in the mean values of the 2 runs between ratings in feelings of pleasantness. General feeling of pleasantness, which combines items of the EWL checklist related to self-confidence and elevated mood, scored higher post-run as compared to pre-run. The mean increase of the two runs for all subjects tested was 2.79 + 5.54 from a total of 19 items. However, again considerable individual differences were striking, therefore the increase did not reach significance[72].
**3**	O'Connor (1991)	Impact of 5 mile outdoor group vs solo run on anxiety	An American pre-post non-controlled study by O'Connor et al. (1991) used 17 members of local running clubs (10 males and 7 females) with a mean age of 25, to compare how a group vs solo 5 mile outdoor run impacted anxiety and body awareness using the State-Trait Anxiety Inventory and Body Awareness Scale as measurements. Both cognitive (STAI) and somatic (BAS) anxiety were reduced following intense running, performed either in the absence or in the presence of interpersonal competition, and that the magnitude of these anxiety reductions were equal in the two conditions. When interpersonal competition was present, post-exercise state anxiety levels (m=27.5) were significantly ($p < 0.05$) below the pre-exercise (m=42.5) and the baseline (m=34) anxiety levels. When interpersonal competition was absent, post-exercise state anxiety levels (m=30) were also significantly below ($p < 0.05$) pre-exercise (m=40) and baseline (m=34) anxiety levels. While body awareness levels were significantly ($p < 0.05$) below both the pre-exercise but not reduced below the baseline value. When interpersonal competition was

			present, post-exercise body awareness (m=27.5) were lower than pre-exercise (32.5), but not below baseline levels (m=24). When interpersonal competition was absent, post-exercise body awareness (m=26) were again, below pre-exercise levels (m=31), but not below baseline (m=24). No significant effect for gender was found[73].
**4**	Nabetani (2001)	Impact of a 10 minute vs a 15 minute treadmill run on mood	A Japan based pre-post non-controlled study by Nabetani et al. (2001) used 15 healthy, moderately active male graduate students with a mean age of 23.4 to compare how two self-selected intensity runs on a treadmill (one for 10 minutes vs the other for 15 minutes) impacted mood using the Mood Checklist Short-form 1 containing three subscales: pleasantness, relaxation and anxiety as measurement. The results found that exercise of 10 and 15 minutes produced similar psychological benefits. Following the 10 minute trial: anxiety (ES = 0.61) significantly decreased ($p<0.01$), whilst there was no significant difference of pleasantness (ES = 0.86) and relaxation (ES = 0.33). Following the 15 minute trial, anxiety (ES = 0.51) and pleasantness (ES = 0.62) significantly decreased ($p<0.01$), but relaxation (ES = 0.07) had no significant pre-post difference[74].
**5**	Bodin (2003)	Impact of 1 hour park vs urban run on depression and anxiety	A Swedish pre-post non-controlled within-subjects study by Bodin et al. (2003) used 12 regular runners (6 female and 6 males) with a mean age of 39.7 to compare how a 1 hour run in a park vs a 1 hour run in an urban environment impacted emotional restoration (depression/ anxiety) using the Exercise-Induced Feeling Inventory and the Negative Mood Scale as measurements. In both men and women, in park and urban settings, running caused a significant decline in anxiety/depression between pre- and post-test measures with $F_{(1,10)} = 16.2$, $p < 0.002$, r=0.78 and a moderate effect size of rs = 0.30. The runners preferred the park to the urban environment in a global sense ($F_{(1, 10)} = 133.07$; $p_{unadjusted} < 0.0001$; $p_{adjusted} < 0.002$) and perceived it as more psychologically restorative, however, results did not indicate any greater emotional benefit from running in the park versus the urban environment, nor that men and women differed[75].
**6**	Butryn (2003)	Impact of 4 mile park vs urban run on mood	An American pre-post non-controlled within-subjects study by Butryn et al. (2003) used 30 non-elite female distance runners with a mean age of 31 to compare how a 4 mile run in a natural setting vs a 4 mile run in an urban setting impacted mood, feeling states and cognition states using the Profile of Mood States, Exercise Induced Feeling Inventory and Thoughts During Running Scale as measurements. Despite 93% of participants preferring running in the park setting, following a 4-mile run regardless of whether the run was completed in a park or urban setting there was a decrease in negative mood and increase in positive mood. Following the park run, total mood disturbance scores decreased 8.97 ($p < 0.001$), while positive engagement, revitalisation and tranquillity all significantly increased ($p < 0.05$, $p < 0.001$ and $p < 0.01$ respectively). A similar effect was found following the urban run: total mood disturbance scores decreased 9.13 ($p < 0.001$), whilst positive engagement and revitalisation significantly increased ($p < 0.05$ and $p < 0.01$ respectively), and tranquillity increased but not significantly[76].
**7**	Kerr (2006)	Impact of indoor vs outdoor 5km run on stress and emotions	A Japanese pre-post non-controlled study by Kerr et al. (2006) used 22 recreational runners with a mean age of 22.7 years to compare how a 5km indoor run on a treadmill vs a 5km outdoor run in a natural environment impacted stress and emotions using the Tension and Effort Stress Inventory as measurement. There were significant pre/post effects for total pleasant somatic emotions [$F_{(1, 21)} = 16.35$, $p< 0.01$], and total unpleasant somatic emotions [$F_{(1, 21)} =7.08$, $p <0.05$]. Post hoc tests indicated that total pleasant somatic emotions increased from pre- (M=12.55), to post-running (M= 14.66), while total unpleasant somatic emotions decreased from pre (M=9.39), to post-session (M=7.77), while irrespective of running condition. There were significant pre/post effects, irrespective of running condition, for relaxation [$F_{(1, 21)} =5.60$, $p< 0.05$], anxiety [$F_{(1, 21)} =9.90$, $p< 0.01$], and excitement [$F_{(1, 21)} =24.65$, $p< 0.001$]. Relaxation and excitement increased,

			and anxiety decreased from pre- (M=4.30; M=2.50; M=3.14 respectively), to post-session (M=4.86; M=3.77; M=2.36 respectively)[77].
**8**	Rose (2012)	Impact of self-paced vs prescribed pace 30 minute treadmill run on self-efficacy	A New Zealand based pre-post controlled study by Rose et al. (2012) used 32 females (17 sedentary and 15 active) with a mean age of 45, to compare how a 30 minute self-paced bout of treadmill exercise vs a 30 minute prescribed-paced bout of treadmill exercise (1 week apart) impacted self-efficacy using the Self-Efficacy for Exercise Scale as measurement. There was a significant main effect for group ($F_{1,28} = 4.74$; P = 0.038; $n^2 = 0.14$), with significantly higher self-efficacy in the active women (M=70.9) than the sedentary women (M=57.7). There was also a significant main effect for condition ($F_{1,28} = 5.81$; P<0.023; n2 = 0.17), with higher self-efficacy before the prescribed condition (M=66.1) compared with the self-selected condition (M=62.6). There was also a significant condition by order interaction ($F_{1,28} = 18.8$; P <0.001; n^2 = 0.39) that showed when the prescribed session was completed first, self-efficacy was equal for the self-selected (M=65.7) and prescribed (M=63.1) conditions, however, when the self-selected condition was completed first, self-efficacy was greater for the prescribed (M=69.0) compared with the self-selected (M=59.4) condition[78].
**9**	Reed (2013)	Impact of rural vs urban 1.5 mile run on self-esteem	A UK based pre-post non-controlled study by Reed et al. (2013) used 75 children aged 11 & 12 to compare how a 1.5 mile run in an urban vs a rural environment impacted self-esteem using the Rosenberg Self Esteem Scale as measurement. Following exercise there was a significant increase in self-esteem ($F_{(1,74),} = 12.2$, p <0.001), however there was no significant difference between the urban or green exercise condition ($F_{(1,74)} =0.13$, p = 0.72), or any significant difference between boys and girls[79].

Supplementary Table S4

Narrative description of findings of the 3 studies with a triple bout of running.

	Author		Narrative description of findings
**1**	Harte (1995)	Impact of 12km outdoor run vs indoor treadmill run with external vs indoor treadmill run with internal stimuli on mood	An Australian pre-post non randomised controlled-repeated measure design by Harte et al. (1995) used 10 male amateur triathletes or marathon runners with a mean age of 27.1 to investigate how an outdoor 12km run, 1 indoor treadmill run with external stimuli, an indoor run with internal stimuli vs a sedentary control impacted mood using the Profile of Mood States as measurement. Following the outdoor run, subjects felt less anxious $F_{(3,35)} = 14.12$ ($p < 0.005$); less depressed $F_{(3,35)} = 4.16$ ($p < 0.01$); less hostile $F_{(3,35)} = 13.13$ ($p < 0.005$); less fatigued $F_{(3,35)} = 15.09$ ($p < 0.005$); and more invigorated $F_{(3,35)} = 13.01$ ($p < 0.005$) than at pre-test; while the two indoor runs had less positive effects on mood[80].
**2**	Berger, Owen + Motl (1998)	Impact of three 15 minute runs of varying intensities (50, 65 or 80% age-adjusted HR max) on mood	Berger, Owen + Motl (1998)[81]<br><br>Study 1 …. A pre-post non controlled study by Berger, Owen + Motl (1998) used 71 USA college students (32 male and 39 female) with a mean age of 21.39 to investigate how three 15 minute runs at intensities of 50, 65 or 80% age-adjusted HR max impacted mood using the Profile of Mood States as measurement. There were significant overall mood benefits for women ($p<0.001$) and for men ($p<0.03$) post-exercise, with all subscales except vigor and fatigue showing significant pre-post changes. No results were provided differentiating the three different intensities of running[81].<br><br>Study 2 ….. A pre-post non controlled study by Berger, Owen + Motl (1998) used 68 USA college students (28 male and 40 female) with a mean age of 22.22 to investigate how three 15 minute runs at intensities of 50, 65 or 80% age-adjusted HR max impacted mood using the Profile of Mood States as measurement. There was significant mood benefits following exercise ($F_{(6.57)} = 6.43$, $p < 0.0001$) and all POMS subscales, apart from fatigue, had significant pre-post improvements reported following running ($p<0.05$). Again, there were no results provided comparing the three different intensities of running[81].
**3**	Markowitz (2010)	Impact of three 20 minute treadmill runs of varying intensities (5% below, 5% above and directly at lactate threshold) on anxiety	An American pre-post controlled trial by Markowitz et al. (2010) used 28 college-aged students with a mean age of 21, to compare anxiety using the State-Trait Anxiety Inventory in 14 active vs 14 sedentary college students following 20 minutes of treadmill exercise at 5% below, 5% above and directly at their lactate threshold. State anxiety improved post-exercise at 5% below ($F(1,21) = 22.781$, $p < 0.001$ and effect size -0.38) and at lactate threshold ($F(1, 21) = 16.223$, $p < 0.001$ and effect size -0.20) but increased at 5% above lactate threshold ($F(1)= 10.891$, $p = 0.003$ and effect size = +0.13[82].

Supplementary Table S5

Narrative description of findings of the 34 studies with longer term intervention of running.

	**Author**		**Narrative description of findings**
**1**	Lion (1978)	Impact of running a mile 3 times per week for 2 months on anxiety and body image in chronic psychiatric patients	An American randomised controlled trial by Lion (1978) used 6 middle aged, chronic psychiatric patients (4 females, 2 male) to compare how running a mile 3 times per week for 2 months (n=3) vs a control group (n=3), impacted anxiety and body image using the State-Trait Anxiety Inventory (STAI) and Rorschach Inkblot Test for body-boundary image as measurements. Post-test anxiety scores on the STAI were significantly reduced in the jogging group compared to the control group (t=3.2, df = 4, $p<0.05$), with the joggers showing an average drop of 9 points on the STAI (39.3 to 30.3) between pre and post-test, while the control group showed an average rise of 4 points (32.6 to 36.6, SD=12). However there was no statistical difference found between the groups for post-test body image scores on the Inkblot test for barrier (t=0.81, df=4. $p<0.05$) or penetration responses (t=0.23, df = 4, $p<0.05$)[83].
**2**	Blue (1979)	Impact of 3 runs per week for 9 weeks on depression	An American pre-post non-controlled study by Blue (1979) used 2 former in-patients of a psychiatric hospital (1 male aged 37 and 1 female aged 32) to examine how 3 runs per week for 9 weeks impacted depression using the Zung depression scale as measurement. Following the running intervention, both patients' depression scores reduced from the category of "moderately depressed" to "mildly depressed", with the male patient reducing his score by 18 points, while the female patient reduced her score by 15 points[84].
**3**	Young (1979)	Impact of a 10 week walking/ jogging programme consisting of 1 hour 3x per week on anxiety and depression	An American pre-post non-controlled intervention study by Young (1979) used 32 adult participants separated into 4 groups by age and sex: young males (n=8, mean age 30.13), middle aged males (n=8, mean age 53.00), young females (n=8, mean age 28.25) and middle aged females (n=8, mean age 50.25). The study investigated how a walking/jogging programme consisting of one hour 3x per week for 10 weeks, impacted anxiety and depression using the Multiple Affect Adjective Checklist as measurement. Results showed significant reductions in pre- to post-test anxiety scores within subject (ANOVA =6.01, $p<0.05$) and also a significant age difference on anxiety in favour of older subjects (ANOVA = 5.37, $p<0.05$, d.f.(1,28)). Results for depression also showed significant age differences in favour of older subjects (ANOVA =5.21, $p<0.05$, d.f.(1,28)), however there was no significant improvement within subject depression scores (ANOVA = 0.25, n.s.)[85].
**4**	Blumenthal (1982)	Impact of 3 times weekly walking-jogging programme for 10 weeks vs 10 weeks of sedentary controls on anxiety and mood	An American non-randomised controlled cohort study by Blumenthal et al. (1982) used 16 healthy adults (11 women and 5 men) with a mean age of 45.1 to compare how a 3-times weekly walking-jogging programme for 10 weeks vs 10 weeks of sedentary controls, impacts anxiety and mood using the Profile of Mood States and the State-Trait Anxiety Inventory as measurements. Results did not detail the number of participants in each group. There were no differences between the exercise and control groups POMS scores at pretesting, but after 10 weeks of training the exercise group exhibited less tension ($F_{(1,30)} = 4.49$, $p <0.04$), less depression ($F_{(1,15)} = 4.82$, $p <0.04$), less fatigue ($F_{(1,30)}= 3.88$, $p <0.05$), less confusion ($F_{(1,15)} = 4.40$, $p <0.05$) and more vigor ($F_{(1,15)}= 3.28$, $p <0.09$) than the sedentary controls. There was no change for either group on the POMS anger subscale. Similarly for State-trait anxiety: there was no difference between the two groups at the time of pretesting, but after the 10 week programme exercisers also exhibited less state anxiety ($F_{(1,26)} = 4.15$, $p <0.05$), and less trait anxiety ($F_{(1,26)} = 6.05$, $p <0.02$)[86].
**5**	Trujillo (1983)	Impact of a 16 week running programme vs weight training vs a control on self-esteem	An American randomised controlled trial by Trujillo (1983) used 35 female college students to compare the impact of a 16 week programme of weight training (n=13) vs running (n=12) vs a physical activity control such as swimming (n=10) control on self-esteem using the Tennessee Self-concept Scale and the Bem Sex Role Inventory as measurements. Results found that both the running and weight training group showed a significant increase in self-esteem from pre- to post-programme ([t,(11)=2.11, $p<0.05$] and [t,(12)= 1.82, $p<0.05$], respectively), however the control group showed a nonsignificant loss in self-esteem [t,(9) =0.55, $p>0.05$]. Although both the weight training and running groups reported significant change in the level of self-esteem, the amount of actual change when compared with between groups was significantly higher for only the weight training group: with the gain scores in weight training as compared to the control group at $t_D(31)=2.83$, $p<0.05$, while the gain scores comparing weight training to running [$t_D(31)=1.00$, $p >0.05$] and running to the control were both non-significant [$t_D(31) = 1.75$, $p>0.05$]. With regards to the

			Bem Sex Role Inventory, the majority of participants in all 3 groups were androgynous in nature at pre-test measurement (n=7, N=11, n=7 for the weight training, running and control groups respectively), with no change occurring at post-test measurement in either of the groups[87].
**6**	Tuckman (1986)	Impact of three 30 minute runs per week on an outdoor running track for 12 weeks on psychological affects in children (creativity, perceptual function, behaviour & self-concept	An American randomised non-controlled trial by Tuckman et al. (1986) used 154 children aged 9-11 to compare how three 30 minute running sessions on an outdoor running track per week for 12 weeks, vs 12 weeks of the school's regular physical education schedule, effected psychological affects such as creativity, perceptual function, behaviour and self-concept, using the Alternate Uses Test, Bender-Gestalt Test, Devereaux Elementary School Behaviour Rating Scale and Piers-Harris Children's Self-Concept Scale, respectively, as measurements. Running significantly improved creativity of school children compared to regular physical education participants (F ratio = 17.00, $p<0.001$), with running treatment children averaging 3 to 5 more creative responses than controls. However running had no significant difference on classroom behaviour (F = 0.91), self-concept (F = 1.02), or perceptual functioning (F = 2.17)[88].
**7**	Doyne (1987)	Impact of 3 runs on an indoor track per week for 8 weeks on depression in women with a diagnosis of major or minor depression	An American randomised controlled trial by Doyne et al. (1987) used 40 women all with a diagnosis of major or minor depression and a mean age of 28.52 to compare the impact of 8 weeks of 3 sessions per week of running on an indoor track vs 8 weeks of weight lifting vs a wait-list control on depression using the Beck's Depression Inventory, Hamilton Rating Scale for Depression and Depression Adjective Checklists as measurements. Results found statistically and clinically significant decreases ($F_{(4, 138)} = 14.98, p < .01$) in mean depression scores from baseline to post-measurements in both running (22.27 vs 8.18) and weight lifting (22.07 to 5.93) relative to the wait-list control group (20.17 to 15.25), with improvements reasonably well maintained at 1 year follow-up, however no significant overall differences found between the two exercise groups[89].
**8**	Fremont (1987)	Impact of 3 runs per week for 10 week on depression, anxiety and mood	An American randomised non-controlled trial by Fremont et al. (1987) used 49 participants (13 male and 36 female) aged between 19 and 62) to compare how 10 weeks of running (3 runs per week) vs 10 weeks of counselling vs 10 weeks of a combination of running and counselling impacted depression, anxiety and mood state using the Beck's Depression inventory, State-Trait Anxiety Inventory and The Profile of Mood States as measurements. There were no significant differences between the three programmes, they all produced similar improvements in depression, anxiety and mood states; with improvement maintained at 4 months follow-up. Depression (BDI), trait anxiety and state anxiety scores all decreased significantly over the 10 weeks ([$F_{(4,184)} =50.3$, $p < 0.0001$]; [$F_{(1, 46)} = 27.1$, $p < 0.0001$]; [$F_{(1,46)} = 21.9$, $p < 0.0001$] respectively). Mood improved over the 10 weeks ($F_{(18,378)} = 4.5$, $p < 0.001$), with significant decreases over time for depression ($F_{(3, 138)} = 23.6$, $p < 0.0001$), confusion ($F_{(3, 138)} = 15.6, p < 0.0001$), anger ($F_{(3, 138)} = 12.4$, $p < 0.0001$), fatigue ($F_{(3, 138)} = 17.9$, $p < 0.0001$), and tension ($F_{(3, 138)} =16.1$, $p < 0.0001$), whilst there was significant increase in vigor over time ($F_{(3,138)} = 14.6, p < 0.001$)[90].
**9**	Hannaford (1988)	Impact of three 30 minute runs per week for 8 weeks on depression and anxiety in psychiatric patients with major psychiatric disorders	An American randomised controlled trial by Hannaford et al. (1988) used 27 male psychiatric patients with major psychiatric disorders and an age range of 25 to 60, to compare the impact of three 30 minute runs per week for 8 weeks (n=9) vs corrective therapy 3 days a week for 8 weeks (n=9) vs waiting list controls (n=9) on depression and anxiety using the Zung Self Rating Depression Scale and State Trait Anxiety Index as measurements. Results found significant reductions in depression scores ($F_{(2,23)} = 3.61$, $p= 0.043$) for the running treatment group compared to the waiting list controls (adjusted means = 45.99 and 51.67, respectively), while the corrective therapy group was intermediate between (adjusted mean = 47.12), but not significantly different from either of the other two groups. Results regarding anxiety scores were in the hypothesized direction, but were not significant ($F_{(2,23)} = 1.085$, $p=0.354$) with the running group not significantly lower than either the corrective therapy group or the waiting list control group (adjusted means = 38.92, 42.76 and 38.98, respectively)[91].
**10**	Long (1988)	Impact of an 8 week running programme consisting of a weekly group session plus twice weekly solo jogs on	A 14 month follow-up from a Canadian randomised non-controlled trial by Long et al. (1988) used 39 chronically stressed, sedentary working women with a mean age of 40 to compare how an 8 week running programme of a weekly group session plus twice weekly solo jogs (n=18) vs 8 weeks of progressive relaxation intervention (n=21) impacted stress, anxiety and self-efficacy using the Trait Anxiety Inventory, Sherer et al.'s inventory for self-efficacy and a modified version of the Ways of Coping Checklist.

		stress, anxiety and self-efficacy	At follow-up, considerably more subjects in the exercise group compared to the relaxation group self-reported program maintenance (67% vs. 14%, respectively). At follow-up, both intervention groups reported significantly less anxiety and greater self-efficacy. In addition, subjects tended to increase their use of problem-focused coping as compared to emotion-focused coping, and 64% of them were still regularly using some structured form of relaxation or exercise. The proportion of subjects reaching clinically significant improvements was 24% at the end of treatment and 36% at the 14-month follow-up.<br><br>Regarding trait anxiety and self-efficacy results showed a significant group main effect ($F_{(2, 36)}$=3.16, p<.05), however, only the univariate for self-efficacy was significant (p<.02). Overall, the exercise group exhibited higher self-efficacy. The time effect for the pre to the post/follow-up average was significant ($F_{(2, 36)}$= 15.38, p<.001) with significant univariate Fs for both measures (both ps<.001). Furthermore, the time effect for post to follow-up approached significance at p<.07, with only the univariate F for trait anxiety significant, $F_{(1, 37)}$=5.01, p<.03. These analyses indicated that both the exercise and relaxation groups maintained treatment effects on self-efficacy, with even further reductions on trait anxiety from post to follow-up. However, despite the exercise group's higher self-efficacy scores, there were no significant interaction effects (Fs<1), suggesting that the exercise and relaxation groups did not change differentially over time.<br><br>Regarding coping, there was a significant group main effect on the two coping dependent measures ($F_{(2, 35)}$ =4.97, p<0.01), with both the exercise and relaxation groups decreased emotion- focused coping and increased problem-focused coping, while total coping scores did not change ($F_{(2, 35)}$ =2.88, p<.07) for the pre to the post/follow-up average contrast. The time effect for post to follow-up was not significant ($F_{(2, 35)}$ = 1.30, p=0.28) indicating that posttreatment changes were maintained at follow-up. Finally, there were no significant interaction effects, indicating that the coping within both groups changed similarly over time (both Fs<1)[92].
**11**	Simons (1988)	Impact of two 30 minute walk/runs per week for 8 weeks on mood	An American non-randomised controlled trial by Simons et al. (1988) used 128 participants consisting of 53 experimental subjects (24 male, 30 female and mean age 44.9) and 75 control subjects (28 male, 47 female and mean age of 42.0) to compare how two 30 minute walk/run per week for 8 weeks vs a weekly 30 minute fitness lecture for 8 weeks effected mood using the Profile of Mood States (POMS), Nowicki-Strickland Internal-External Control Scale for Adults (ANSIE) and Marlowe-Crowne Social Desirability Scale for measurements. Exercise class subjects had significant improvement in mood compared to non-treatment controls, with mean pre- to post-test summed mood change scores improving significantly for experimental (28.8 to 15.6) in comparison with control subjects (23.5 to 20.9), $F_{(1,126)}$ = 4.46, p < 0.05. There was also significant improvement in pre- to follow-up mood change scores, $F_{(1,98)}$ = 7.63, p < 0.01. Mood improvement was predicted by initial mood, with improvement limited to the most mood-disturbed subjects[93].
**12**	Moses (1989)	Impact of varying intensity 10 week walk-jog programmes on mood and mental wellbeing	A British randomised controlled trial by Moses et al. (1989) had 75 sedentary adult volunteers with an average age of 38.8 years who were assigned to one of four 10 week conditions: high intensity aerobic walk-jog programme (n=18), moderate intensity walk-jog programme (n=19), attention-placebo including strength, mobility and flexibility exercises (n=18) or a waiting list control (n=20). The study compared the 4 conditions effects on mood and mental wellbeing using the Profile of Mood States and the Hospital Anxiety and Depression Scale as measurements. There were no significant differences before training between groups on any of the POMS, coping or self-efficacy measures. There was a significant group by time interaction for ratings on the tension/anxiety scale of the POMS [$F_{(3,71)}$ = 2.94, p<0.05], with reductions in tension/anxiety reported only by subjects in the moderate exercise condition. There were also significant differences in the POMS subscale of confusion, were there were differences over time [$F_{(1,71)}$ = 3.70, p<0.06] and group by time [$F_{(3,71)}$ = 2.61, p<0.06], with greater decreases in the moderate exercise group (mean change - 0.193) than in the high exercise (-0.039), attention-placebo (-0.0003) or waiting list (+0.008) conditions. No significant effects were found on the perceived coping scales, but there was significant effects on the physical well-being scale [$F_{(3,71)}$ = 3.82, p<0.01], with all three active treatment groups showed improvements after the 10 week programmes, while the waiting list group ratings decreased. (+0.046, + 0.046 and +0.146, in the high intensity, moderate intensity and attention-placebo condition, respectively). At follow-up there was a significant group by time interaction on the coping deficits scale [$F_{(2,55)}$

			= 3.45, p<0.05] and ratings of depression/ dejection [$F_{(2,55)}$ = 3.00, p<0.06] with decreases reported in the moderate exercise group, but not in the high exercise or attention-placebo conditions. Also, the group by time interaction approached significance for the perceived coping assets scale [$F_{(2,55)}$ = 2.56, p<0.08] where again, positive changes were confined to subjects in the moderate exercise condition[94].
**13**	Ossip-Klein (1989)	Impact of running on an indoor track 4 times per week for 8 weeks on self-concept in clinically depressed women	An American randomised controlled trial by Ossip-Klein et al. (1989) used 32 clinically depressed women with an average age of 28.52 to compare the effects of 8 weeks of running 4 times weekly on an indoor track vs weight lighting 4 times weekly vs a delayed treatment (assessment only) control on self-concept using the Beck Self-Concept Test as measurement. Results did not detail the number of participants in each group. No significant differences between exercise groups were found, with results showing that both running ($F_{(3,99)}$ =7.62, p<0.0001) and weight lifting ($F_{(3,99)}$ = 11.92,p <0.0001) exercise programs significantly improved self-concept in the clinically depressed women compared to wait-list controls. Scores for the track and universal conditions were significantly higher than those for the wait-list condition at post treatment for the Beck Self-Concept Test ($F_{(2, 33)}$= 4.69, p<0.05). Improvements were also reasonably well-maintained over time. In general, no significant differences were found between exercise groups; but where differences did occur, they slightly favoured the weightlifting group[95].
**14**	Morris (1990)	Impact of stopping running for 2 weeks on anxiety and depression	A UK based pre-post study with randomised comparison by Morris et al. (1990) used 40 male regular runners with a mean age of 37 years to compare how stopping running for 2 weeks (n=20) vs continuing to run as normal (n=20) over a 6 week timeframe impacted anxiety and depression using the General Health Questionnaire and short forms of the Zung Anxiety and Zung Depression scales as measurements. The groups did not differ at baseline on any scale (all Fs for group main effects and interactions). Scores on the GHQ subscales, Somatic Symptoms, Anxiety/Insomnia and Social Dysfunction, were all significantly greater in deprived than in continuing runners after both the first and second week of deprivation, and significantly more deprived (11 & 9 subjects in weeks 3&4 respectively) than non-deprived subjects (3 & 2 subjects in weeks 3&4 respectively) exceeded the suggested cut-off score for a psychiatric case after both the first and second weeks of deprivation (x^2 = 5.38, 4.51, respectively, df = 1, p <0.05). Symptoms of depression were greater in the withdrawn than in the control group at the end of the second week of withdrawal, the effect reached significance by a randomization test (t = 2.33, df = 38, p < 0.05, l-tailed). There was a tendency for a similar, although reduced, effect after the first week of resumed running (Table IV) but this did not reach significance (t = 1.60, p = 0.05 at 1.68). A significant difference between the groups arose only after the second week of deprivation, with scores in the Zung depression and anxiety scales only reaching significance after week 2 of deprivation ($F_{(1,37)}$ = 22.64, p<0.001 for depression and $F_{(1,37)}$ =11.51, p<0.01 for anxiety). Despite the tendency for the deprived group to continue to decline from weeks 5-6 in anxiety and depression scores, there was no statistical difference between the groups once the deprived group resumed running[96].
**15**	Friedman (1991)	Impact of 12 weeks of jogging on stress and mood	An American randomised controlled trial by Friedman et al. (1991) used 387 students (188 female and 117 male) with an average age of 20.0 to compare how 12 weeks of either jogging (n= 84), relaxation (n=96), group interaction (n= 100), and lecture-control (n= 107) impacted stress and mood using the Profile of Mood States and Bem Sex Role Inventory as measurements. In initial measures the relaxation response, jogging, group interaction, and lecture-control groups did not differ on psychological masculinity [$F_{(3, 367)}$= .38], femininity [$F_{(3, 367)}$= .38] or the six POMS subscales. High masculinity male and female joggers reported significantly more mood improvement than those who were low, indicating that psychological masculinity, rather than gender, was associated with joggers' short-term mood improvement. Although all women reported significant mood benefits, high masculinity women benefitted more than low masculinity women, with greater reductions in tension, depression, and anger. For men, psychological masculinity was related to benefits on tension and vigor, but not on the other subscales. Although all women reported significant reductions in depression after the relaxation and jogging sessions, women joggers who were high in psychological masculinity experienced significantly greater reductions than low masculinity joggers (p<0.04). The interaction between technique, gender, moderating variable, and pre-post session was significant for masculinity [$F_{(18,843.4)}$ = 2.14, p < .004], but not for femininity [$F_{(18,843.4)}$ =.62]. Femininity had a significant effect on combined POMS scores [$F_{(6, 297)}$= 2.79, p< .02], with higher psychological femininity associated with higher tension, depression, and fatigue and lower vigor and confusion scores

			compared to those low in femininity. There were significant pre-post session x technique interactions for both high and low masculinity women [$F_{(18, 843.36)}$= 2.47, p<0.0007; $F_{(18, 843.36)}$= 2.49, p<0.0006, respectively]. In both the jogging and group interaction techniques, the masculinity x pre-post session interaction was significant [$Fs_{(6,298)=}$ 3.32,3.53;p's < .004,.003,respectively]. Short- term improvements in POMS scores depended upon masculinity for women joggers and participants in group interaction. For the relaxation response and lecture-control groups, the hypothesized interactions between masculinity and pre-post session were not significant [$Fs_{(6,298)}$ = 1.48, 1.15;p's < .19, .38]. Short-term improvements in mood did not depend on masculinity in these groups; women reported significant improvements in mood from pre- to post session [$Fs_{(6,298)}$ = 6.01, 4.36; p's< .0001, .0003][97].
**16**	Williams (1991)	Impact of 4 weeks of treadmill running 5 times per week at set paces reflecting 50, 60 & 70% VO_2 max on mood	An American pre-post non-controlled within-subject design by Williams et al. (1991) used 10 moderately trained male runners with a mean age of 25.6 to assess the impact of 4 weeks of treadmill running 5 times a week at set paces reflecting 50, 60 & 70% VO_2 max, on mood using the Profile of Mood States as measurement. The within-subject data indicated a positive correlation, showing that an increase in mean VO_2 (decrease in RE) is associated with an increase in mood disturbance, as reflected by the total mood disturbance score (r = 0.88; p<0.01) as well as 5 of the 6 POMS subscales: tension (r = 0.81; p<0.01), depression (r = 0.73; p<0.01), anger (r =0.58; p<0.01), vigor (r = -0.60; p<0.01), fatigue (r = 0.18; not significant) and confusion (r = 0.60; p<0.01). This positive correlation indicates that, when the focus of attention was on within-subject variation, weeks featuring more economical values were associated with more positive mental health profiles. However, in moderately trained male runners considered as a group, there is no relationship between running efficiency and total mood disturbance[98].
**17**	Kerr (1993)	Impact of a weekly 40 minute fixed distance run (5km for females, 6.6km for males) through a wooded area for 7 weeks on mood	A Netherlands based pre-post non-controlled study by Kerr et al. (1993) used 32 regularly exercising university students (18 male and 14 female) aged between 18 & 22 to investigate the effect of a weekly 40 minute fixed distance, running session in a wooded area (5.0km for females, 6.6km for males) for 7 weeks on mood using the Stress-Arousal Checklist and Telic State Measure as measurements. Over the running programme, subjects' mood experience was generally pleasant, characterized by high arousal and low stress. In males, from pre- to post-running there were significant increases in TSM felt arousal scores ($F_{(1,16)}$=52.37, p=0.0001), SACL arousal scores (F($_{1,16)}$= 15.34, p=0.001) and TSM preferred arousal scores ($F_{(1,16)}$ = 4.49, p=0.05). In contrast, TSM arousal discrepancy scores were found to decrease significantly for males ($F_{(1,16)}$= 6.74 , p=0.02) pre- to post-running. Similar significant effects were observed pre-post running for females, with strongly significant increases in TSM felt arousal scores ($F_{(1,12)}$=16.16 ,p=0.002), SACL arousal scores ($F_{(1,12)}$=25.19, p=0.0001) and TSM preferred arousal scores ($F_{(1,12)}$= 11.82, p=0.005). Female runners' TSM arousal discrepancy scores also decreased significantly ($F_{(1,12)}$= 11.86, p=0.005) pre- to post-running. When comparing mood responses of fast runners to slow runners, both female and male fast runners scored higher on TSM felt arousal than slow runners ([$F_{(1,12)}$= 6.50, p=0.03] and [$F_{(1,16)}$= 4.97, p=0.04], respectively)[99].
**18**	Long (1993)	Impact of 3 runs per week for 10 weeks on anxiety and stress	A Canadian randomised controlled trial by Long (1993) used 35 participants (14 males and 21 females) with a mean age of 35.6 to compare the effects of running 3 times per week for 10 weeks (n = 12) vs stress inoculation for 10 weeks (n = 9) vs waiting list controls (n = 14), on anxiety and stress using the Cornell Medical Symptom Checklist as measurement. Although the exercise group was more likely to report using exercise to cope with stress, compared to the stress inoculation group, there was no significant differences found between groups on stress or coping classifications. There were also no significant difference of scores of the Cornell Medical Symptom Checklist between the aerobic conditioning and the stress inoculation treatment groups (F<1; M = 87.4, SD = 16.7; Ms = 86.2, SD = 13.5, respectively)[100].
**19**	Berger & Friedman (1998)	Impact of three jogs per week for a minimum of 20 minutes over 12 weeks on stress and mood	An American randomised controlled trial by Berger & Friedman (1998) used 387 undergraduate college students (188 women and 117 men) with an average age of 20.0 to compare how: jogging three times per week for a minimum of 20 minutes per session over 12 weeks (n=84) vs 12 weeks of relation response (n=96), 12 weeks of discussion groups (n=100) and a control group (n=107), impacted stress and mood using the Profile of Mood States as measurement. All three techniques were significantly more effective in reducing stress than the control activity (p<0.03): with joggers, students practicing the relaxation response, and discussion group members collectively reporting significantly greater stress reduction than the control group during October $F_{(18, 280)}$ =1.79, p<0.03, and November, $F_{(18, 280)}$ =1.85,

			$p<0.03$. However, jogging and practice of the relaxation response were significantly more beneficial in helping students reduce short-term stress than group support ($p<.04$), with joggers and members of the relaxation response group reporting larger and more numerous reductions in tension, depression, and anger than members of the discussion and control groups. Changes in vigor, fatigue, and confusion were sporadic. There were no long-term benefits observed[101].
**20**	Berger & Owen (1998)	Impact of twice weekly walking/ jogging for 14 weeks on mood and anxiety	An American pre-post with comparison study by Berger & Owen (1998) used 91 college students to compare how 14 weeks of twice weekly walking/jogging (n=67, 35 female and 32 male) vs a weekly health science class (n =24, 15 female and 9 men) impacted mood and anxiety using the Profile of Mood States and State-Trait Anxiety Inventory as measurements. The interaction between exercise intensity and pre-post mood benefits was not significant ($F_{(12,50)} = 1.27$, ns), however, joggers reported short-term mood benefits on the combined subscales of the Profile of Mood States, and each subscale contributed to the benefits. Regardless of their exercise intensities, the pre-post-test exercise effect was significant ($F_{(6,56)} = 4.87$, p < ,0005), with joggers reporting significant pre-post exercise mood changes on each of the six subscales of POMS: tension (F=15.67, p <.0002), depression (F=15.64, p< .0002), anger (F=12.77, p< .0007), vigor (F= 22.29, p<.00005), fatigue (F=20.14,p< .00005), and confusion (F=26.34, p<.00005). Regarding sex differences, the largest interaction was on the fatigue subscale with women's scores decreasing more after jogging than the men's (Fl,6,=9.85)', while the F ratios (1 and 61 df) for the other subscales were for tension 0.60, depression 1.17, anger 0.33, vigor 1.96, and confusion 1.50[102].
**21**	Szabo (1998)	Impact of running vs non-running days on anxiety and mood over 21 consecutive days	A UK based pre-post non-controlled observational cohort study by Szabo et al. (1998) used 40 members of an amateur running club (30 males with a mean age of 40.5, and 10 females with a mean age of 37) to assess how anxiety and mood (exhaustion, tranquillity, positive engagement and revitalization) varied on running vs non-running days over 21 consecutive days, using daily night time recording of the their own individual running time/distance on running days and the Commitment to running scale, Spielberger State Anxiety Inventory and Exercise induced Feeling Inventory as measurements. There were statistical differences, but small effect sizes, between average values for anxiety and mood on running and non-running days, with runners reporting lesser anxiety and better mood on running days in contrast to non-running days. Mean state anxiety on running days was 35.7 (SD=7.1) compared to 37.2 (SD=7.9) on non-running days, with a period main effect ($F_{(1,38)}$=5.22, p<0.03). All subscales for mood (exhaustion, tranquillity, revitalisation and positive engagement) were significantly different (p<0.05) on running days as compared to non-running days, with ($F_{(1,38)}$=4.34,p<0.04) for exhaustion; ($F_{(1,38)}$=5.56, p<0.02) for tranquillity; ($F_{(1,38)}$=18.32, p<0.001) for revitalisation and ($F_{(1,38)}$=11.79, p<0.001) for positive engagement. There were gender differences in the commitment to running ($F_{(1,36)}$=10.5, p<0.03) with males having a higher value than females[103].
**22**	Broman-Fulks (2004)	Impact of six 20 minute treadmill sessions of either high or low intensity aerobic exercise across 2 weeks on anxiety sensitivity in participants with elevated anxiety sensitivity scores	An American randomised non-controlled trial by Broman-Fulks et al. (2004) used 54 participants (41 women) with elevated anxiety sensitivity scores with a mean age of 21.17 to compare how six 20 minute treadmill exercise sessions across 2 weeks of either high intensity aerobic exercise (n=29) vs low intensity aerobic exercise (n=25) impacts anxiety sensitivity using the Anxiety Sensitivity Index, State-trait Anxiety Inventory and Body Sensations Questionnaire as measurements. Results indicated that both high- (34.17 to 25.03) and low-intensity (31.44 to 28.56), exercise reduced anxiety sensitivity. However, high-intensity exercise caused more rapid reductions in a global measure of anxiety sensitivity and produced more treatment responders than low-intensity exercise [x^2(1, N = 54) = 6.27, p = 0.01]. A significant simple effect for assessment session emerged for the high-intensity exercise group, $F_{(2, 56)}$ = 42.50, p <0.001, n^2 = 0.60. An assessment session effect was also found for the low-intensity comparison group, ($F_{(2, 48)}$ = 13.72, p < 0.001, n^2 = 0.36). State anxiety mean scores from pre and post-intervention decreased for high intensity (35.10 to 32.03) but increased from low intensity running (35.12 to 38.24), while trait anxiety decreased for both high (41.67 to 38.79) and low intensity running (42.72 to 42.32), however there were no significant effects for either state or trait anxiety. Only high-intensity exercise reduced fear of anxiety-related bodily sensations ($F_{(1, 52)}$ = 9.44, p <0.01, n^2 = 0.15) with mean BSQ scores for the high-intensity exercise group (M = 2.12, SD = 0.10) significantly lower on average compared to the low-intensity comparison group (M = 2.56, SD = 0.11)[104].
**23**	Haffmans (2006)	Impact of running therapy for 3 days per	A randomised controlled trial from the Netherlands by Haffmans et al. (2006) used 60 psychiatric patients (19 men, 41 women) in a day treatment programme for affective disorders who had a mean age of 39 and were all suffering from a

		week for 12 weeks on depression and self-efficacy in psychiatric patients all suffering from depression	depressive disorder. They compared the impact of running therapy 3 days per week for 12 weeks (n=20) vs physio training therapy (n=21) and a control (n=19) on depression and self-efficacy using the Hamilton Rating Scale for Depression (HRSD), Becks Depression Inventory (BDI), Self-Efficacy Scale and Physical Self-Efficacy Scale (PSES) as measurements. Although both groups were positive about the training programme, participants in the PT group gave a significantly higher evaluation than participants in the running group (p<0.05). After 6 weeks, no significant differences were found between both the training groups and the control group; however, after 12 weeks, the physio training group showed significant improvement on scores for blind-rated HRSD and BDI scores (p= 0.004 and p=0.002, respectively). The running group had no significant difference in depression scores from baseline (26.7) to 12 weeks(25.5). Regarding self-efficacy, the RT group scored significantly higher in PSES after 6 weeks (p=0.03), feelings of self-efficacy did not change significantly in either the running or the physio groups after 12 weeks. Running group feelings of self-efficacy was 46.6 at baseline and 49.1 at 12 weeks[105].
**24**	Thornton (2008)	Investigating the relationship between anxiety and marathon	An American cross sectional survey by Thornton et al. (2008) used 50 runners over age 18 to investigate the relationship between anxiety and marathon training using the Beck Anxiety Inventory as measurement. Mean anxiety scores decreased from baseline pre-training levels (0.9) compared to 2 months prior to marathon day (to 0.7, respectively, with 72% had no change from baseline pre-training levels on the Beck Anxiety Inventory (0.9) compared to 2 months prior to marathon day (0.7; 72% had no change from baseline, 22% were less anxious and 6% were more anxious than baseline). However, anxiety scores increased as race day approached: at 1 month prior to race day (1.4; 46% had no change from baseline, 19% were less anxious and 35% were more anxious than baseline) and 1 week prior to race (2.6; 22% had no change from baseline, 14% were less anxious and 64% were more anxious than baseline, respectively). Overall results found that marathon training decreased anxiety initially, but overall anxiety increased as race day approached[106].
**25**	Scholz (2008)	Impact of a 1 year marathon training programme on self-efficacy	A pre-post non-controlled non-experimental longitudinal study based in Switzerland by Scholz et al. (2008) used 30 untrained participants (26 women, 4 men) with a mean age of 41.2 to investigate how a 1 year marathon training running programme impacted self-efficacy using a 4 part author-created measurement. There were no statically significant differences in baseline level, trend or fluctuation of self-efficacy between the participants who successfully completed the marathon, and those who did not. Self-efficacy had a baseline level mean of 3.45 (p<0.01), a linear trend mean of -0.05 (p<0.05), a fluctuation mean of 0.33 (p<0.01) and an intra-class correlations of 0.46. Baseline level of self-efficacy was positively associated with baseline level in running (correlation analyses =.27;p<0.05 (95% confidence intervals = .00; .53) and fluctuation in self-efficacy correlated positively with fluctuation in running .39;p<0.05 (95% confidence interval .03;.74). A substantial correlation between the trend in running and self-efficacy emerged as well (.39, not significant but was associated with a very wide confidence band (95% Confidence interval -.10; .87) and was not significant. As this was a non-experimental longitudinal study, no causal statements can be drawn, hence cannot conclude that, self-efficacy leads to increased levels of exercise and vice versa[107].
**26**	Kalak (2012)	Impact of daily 30 minute morning runs on weekdays for 3 weeks (ie. 3x5 runs) on stress and mood	A Swiss randomised controlled trial by Kalak et al. (2012) used 51 adolescents (27 female and 24 male) with a mean age of 18.3 to compare the effects of a daily 30 minute morning run on weekdays for 3 weeks (ie. 3 x 5 runs) (n=27) vs a control group (n=24) on stress and mood using the Perceived Stress Scale, a daily mood log and a questionnaire assessing positive and negative comping strategies as measurements. Perceived stress and positive/ negative coping strategies did not differ significantly between the running and the control groups ($F_{(1,49)}$ =1.71, n^2=0.034, not significant) nor was there a statistically significant group x time interaction ($F_{(1,49)}$ =2.97, n^2=0.057, not significant). Mood in the morning was significantly higher in the running group than the control group ($F_{(5,245)}$ =4.42, n^2=0.083, p<0.001); the group x time interaction was also significant ($F_{(5,245)}$ = 6.32, n^2 = 0.114, p<0.01); mood in the morning also increased significantly over time in the RG compared with the CG ($F_{(5,245)}$ =16.08, n^2 = 0.247, p<0.05). Over time, irrespective of group, mood in the evening improved, but there was no difference of mood in the evening between groups and the group x time interaction was not significant[108].
**27**	Inoue (2013)	Impact of 10 organised runs on self-sufficiency in homeless people	An American pre-post non-controlled study by Inoue et al. (2013) used 148 homeless people involved in the "Back on my feet" programme who had an average age of 29.9 and 90.5% of whom were male. They examined the impact of 10 organised runs on self-sufficiency using an author-created scale to measure the psychological benefits of the program. Results suggested that increases in running

			involvement had a significant positive correlation with perceived self-sufficiency (r = .30, p < 0.01). The mean value of perceived self- sufficiency (M= 5.95) exceeded the midpoint (4.0), indicating on average participants agreed the program provided increased psychological benefits associated with self-sufficiency. Results suggested that the participants gained higher levels of perceived self-sufficiency as they became more involved with running during the program, with the regression model showing a significant proportion of the variation in perceived self-sufficiency (F = 3.39, p < .01, Adjusted R2 = .08), and increases in running involvement was the sole significant predictor of the outcome (β = .29, t = 3.57, p < .01)[109].
**28**	Samson (2013)	Impact of a 15 week marathon training program consisting of 3 group training days per week plus one run of 8-20 miles on the weekend, on general affect & self-efficacy	An American pre-post non-controlled study by Samson et al. (2013) used 39 Caucasian university students (11 males and 28 females) who all had running experience and a mean age of 20.5. They examined how a 15 week marathon training program, consisting of 3 group training days per week plus one training run of 8-20 miles during the weekend, impacted general affect and self-efficacy using the Positive and Negative Affect Scale (PANAS) and author-created measurements for self-efficacy. Results showed a significant increase in self-efficacy over the training programme ($F_{(12,444)}$=5.81, p<0.01, partial eta^2 =0.136). While there was a significant decrease of positive affect over time ($F_{(12,444)}$=8.35, p<0.01, partial eta^2=0.184), there was no significant change found for negative affect over the programme[110].
**29**	Doose (2015)	Impact of group walking/running 3 times per week for 8 weeks on depression	A German randomised controlled trial by Doose et al. (2015) used 46 outpatients aged 18-65 diagnosed with mild to severe depression to compare the impact of an 8 week, 3-times weekly, group walking/running exercise programme (n=30) vs wait list (n=16) on depression using the Hamilton Rating Scale and Beck Depression Inventory as measurements. Out of forty-six participants, 11 participants (24%) dropped out: 7 (23%) from the intervention group and 4 (25%) from the control group. While both the exercise intervention and control group had reductions in scores for the Hamilton Rating Scale for Depression (-9.48 and -1.2, respectively), results attributed a large and clinically significant change to the exercise intervention (Cohen's d= 1.8; mean change = 8.24; p = <0.0001). There were moderate changes in Becks Depression Inventory scores without clinical significance (Cohen's d: 0.50; mean change = 4.66; p=0.09), with the intervention group BDI scores reducing a mean of -8.20, while the control had a mean reduction of -3.54[111].
**30**	Von Haaren (2015)	Impact of a 20 week running training course on stress and mood during academic examinations	A German randomised controlled trial, within subject design by Von Haaren et al. (2015) used 61 inactive male university students with a mean age of 21.4 to compare how a 20 week aerobic running training course vs a waiting list control, impacted stress and mood during academic examinations using a shorten mood scale based on the Multidimensional Mood Questionnaire and a one item test for perceived control and stress as measurements. Results did not detail the number of participants in each group. Significant emotional stress reactivity was evident in both groups during both academic assessment episodes, with an increase of mean perceived stress of 0.23 in control group, and 0.21 in aerobic group in the first academic assessment. However, participants in the aerobic training group showed lower emotional stress reactivity compared with the control participants after the 20-week training programme, with perceived stress of the aerobic group remaining similar during both exam periods(2.27 to 2.24), however it increased further in the control group (2.43 to 2.51). After both academic assessment periods, the coefficient for the group by perceived stress interaction was higher (*B*= -0.18, p<0.001) compared with just the first academic assessment (*B*= -0.11, p<0.05), descriptively indicating a larger effect of the group x PS interaction at the end of the 20 week exercise programme[112].
**31**	Kahan (2018)	Impact of 20 running sessions alternating between game vs lap running on self-esteem and self-efficacy in children	An American pre-post with comparison study by Kahan et al. (2018) used 11 children (9 males and 2 females) aged 9 & 10 to compare the impact of 20 running sessions alternating between game vs lap running on self-esteem and self-efficacy using a 50-item, author-created questionnaire as measurement. High inherent interest participants (ie, higher MVPA% in the running laps condition) had statistically significant higher scores than low inherent interest participants on Recognition (p=0.01), Ego Orientation (p=0.03), and Expectancy Beliefs (p=0.03) subscales. No differences were detected between high and low response to treatment groups. Results for self-esteem: Cronbach's alpha score was 0.69 and self-esteem mean was 3.63 on a 5 point scale; while mean for task-efficacy was 4.16 on a 5 point scale. PA% (62.2% vs 76.1%, effect size [ES] = −0.65) was lower and moderate-vigorous physical activity (MVPA%) (33.3% vs 15.8%, ES = 0.75) and MVPA% of PA (53.6% vs 20.2%, ES = 0.91) were higher during game vs lap running conditions[113].

**32**	Keating (2018)	Impact of 12 weeks of twice weekly running in a group setting that offers social support supervised by clinical professionals, on stress, anxiety and depression	A Canadian pre-post non-controlled study by Keating et al. (2018) used 46 participants with complex mood disorders (11 males and 35 females) consisting of 29 youths (mean age 22.1) and 17 adults (mean age 45.2), to examine how 12 weeks of twice weekly running groups impacted stress, anxiety and depression using the Cohen's Perceived Stress Scale, Becks Depression Inventory, Becks Anxiety Inventory and Short Form Survey as measurements. Adults and youths with complex mood disorders benefited from the running therapy programme supervised by clinical professionals in a group setting that offers social support. There were significant decreases in depression ($F=4.5$, $df=11, 201$, $p<0.0001$), anxiety ($F=4.8$, $df=11, 186$, $p<0.0001$) and stress ($F=2.3$, $df=11, 186$, $p=0.01$) scores from baseline to the end of the study. Mean BDI scores from baseline to post-exercise intervention, decreased by 39% in adults from high (30.8) to low levels (18.8) and by 27% in youths from moderate (26.9) to reduced moderate levels (19.5). Younger participant age, younger age at onset of illness and higher perceived levels of friendship with other running group members (ps ≤ 0.04) were associated with lower end-of-study depression, anxiety and stress scores, while higher attendance was associated with decreasing depression and anxiety (ps ≤ 0.01) scores over time[114].
**33**	Nezlek (2018)	Impact of 3 months of self-prescribed running on psychological wellbeing, self-esteem, self-efficacy and affect	A Polish pre-post observational cohort study with no control by Nezlek et al. (2018) used 244 recreational runners with a mean age of 32.5 and 48% of whom were women, to investigate how the volume of recreational running could impact psychological wellbeing, self-esteem, self-efficacy and affect. Over 3 months the participants recorded weekly how far they had run and the psychological outcomes were measured using the Rosenberg Self-esteem Scale, the Satisfaction With Life Scale and a circumplex model that distinguishes the valence (positive or negative) and arousal (activated or deactivated) of affect. Results found a positive within-person relationships between how much people ran each week and self-reports of well-being. The more often and farther people ran during a week, the better they thought about themselves and their lives and the better they felt affectively. Results found that sex and age did not significantly moderate any of the relationships reported above (all ps > .13), however, how long people had been running significantly moderated the slope between number of days run each week PA ($\gamma_{11} = -.033$, $p < .05$). Self-efficacy was related to distance run, but not to frequency. When analyzed separately, both measures of running were significantly related to all measures of well-being, such that well-being was higher during weeks when individuals ran more often and further than it was during weeks when they ran less often and less far. By contrast, the average kilometers people ran each week moderated most relationships between running and well-being such that relationships between well-being and running were weaker for people who ran more than they were for people who ran less. For the kilometers people ran each week, significant moderation was found for weekly Satisfaction with Life Scale ($\gamma_{11} = -.0002$, $p = .013$), self-esteem ($\gamma_{11} = -.0002$, $p = .015$), positive activated affect ($\gamma_{11}=-.0003$, $p<.001$), positive deactivated affect ($\gamma_{11}=-.0008$, $p<.01$), negative activated affect ($\gamma_{11}= .0002$, $p = .046$), and negative deactivated affect ($\gamma_{11} = .0003$, $p = .01$). Satisfaction with progress fully mediated relationships between all measures of well-being and number of days run each week and between all measures of well-being and kilometers run each week. In all of these analyses including self-efficacy and self-esteem, the direct effect of the predictor (running) on the outcome (well-being) was not significant ($p>0.12$), whereas the direct effect of the mediator (satisfaction with progress) was ($p<0.001$), and the indirect effect of running was significant (the critical path for demonstrating mediation) ($p<0.001$)[115].
**34**	Kruisdijk (2019)	Impact of 6 months of running-walking for one hour twice a week on depression in subjects with major depressive disorder	A randomised controlled trial from The Netherlands by Kruisdijk et al. (2019) used 48 participants with major depressive disorder with a mean age of 41.6 to compare how 6 months of running-walking for one hour twice a week (n=25) vs a control group (n=23) impacted depression using the Hamilton Depression Scale as measurement. Depression on the HDS decreased in both the intervention and the control group on average by 2–3 points after 3 months, but there was no significant difference or effect on depression in favour of the intervention group (Cohen's $d < 0.2$, $F = .13$, $p = 0.73$). Conclusions about the anti-depressive effect of this exercise intervention were not possible due to only 9 participants (19%) completing the study, low statistical power and lack of follow-up at six and 12 months. An integrated lifestyle intervention may be more effective than a single add-on exercise intervention for patients with major depressive disorder, as these results found that the exercise intervention and study design weren't feasible in terms of motivation and compliance[116].

References

1. Wilson VE, Morley NC, Bird EI. Mood profiles of marathon runners, joggers and non-exercisers. *Percept Mot Skills*. 1980;50(1):117-118. doi:10.2466/pms.1980.50.1.117

2. Joesting J. Running and Depression. *Percept Mot Skills*. 1981;52(2):442-442. doi:10.2466/pms.1981.52.2.442

3. Jorgenson DE, Jorgenson CB. Perceived Effects of Running/Jogging: A Social Survey of Three Clubs. *Int Rev Sport Sociol*. 1981;16(3):75-85. doi:10.1177/101269028101600305

4. Valliant PM, Bennie FA, Valiant JJ. Do marathoners differ from joggers in personality profile: a sports psychology approach. *J Sports Med Phys Fitness*. 1981;21(1):62-67.

5. Francis KT, Carter R. Psychological characteristic of joggers. *J Sports Med Phys Fitness*. 1982;22(3):386-391.

6. Hailey BJ, Bailey LA. Negative addiction in runners: A quantitative approach. *J Sport Behav*. 1982;5(3):150-154.

7. Callen KE. Mental and emotional aspects of long-distance running. *Psychosomatics*. 1983;24(2):133-134, 139, 141, passim. doi:10.1016/S0033-3182(83)73239-1

8. Galle PC, Freeman EW, Galle MG, Huggins GR, Sondheimer SJ. Physiologic and psychologic profiles in a survey of women runners. *Fertil Steril*. 1983;39(5):633-639. doi:10.1016/s0015-0282(16)47058-5

9. Lobstein DD, Mosbacher BJ, Ismail AH. Depression as a powerful discriminator between physically active and sedentary middle-aged men. *J Psychosom Res*. 1983;27(1):69-76. doi:10.1016/0022-3999(83)90111-3

10. Rudy EB, Estok PJ. Intensity of jogging: its relationship to selected physical and psychosocial variables in women. *West J Nurs Res*. 1983;5(4):325-336. doi:10.1177/019394598300500406

11. Goldfarb LA, Plante TG. Fear of fat in runners: an examination of the connection between anorexia nervosa and distance running. *Psychol Rep*. 1984;55(1):296. doi:10.2466/pr0.1984.55.1.296

12. Guyot GW, Fairchild L, Nickens J. Death concerns of runners and nonrunners. *J Sports Med Phys Fitness*. 1984;24(2):139-143.

13. Rape RN. Running and Depression. *Percept Mot Skills*. 1987;64(3_suppl):1303-1310. doi:10.2466/pms.1987.64.3c.1303

14. Weight LM, Noakes TD. Is running an analog of anorexia?: A survey of the incidence of eating disorders in female distance runners. *Med Sci Sports Exerc*. 1987;19(3):213-217.

15. Chan CS, Grossman HY. Psychological effects of running loss on consistent runners. *Percept Mot Skills*. 1988;66(3):875-883. doi:10.2466/pms.1988.66.3.875

16. Frazier SE. Mood state profiles of chronic exercisers with differing abilities. *Int J Sport Psychol*. 1988;19(1):65-71.

17. Lobstein DD, Ismail AH, Rasmussen CL. Beta-endorphin and components of emotionality discriminate between physically active and sedentary men. *Biol Psychiatry*. 1989;26(1):3-14. doi:10.1016/0006-3223(89)90003-6

18. Lobstein DD, Rasmussen CL, Dunphy GE, Dunphy MJ. Beta-endorphin and components of depression as powerful discriminators between joggers and sedentary middle-aged men. *J Psychosom Res*. 1989;33(3):293-305. doi:10.1016/0022-3999(89)90020-2

19. Nouri S, Beer J. Relations of moderate physical exercise to scores on hostility, aggression, and aggression, and trait-anxiety. *Percept Mot Skills*. 1989;68(3 Pt 2):1191-1194. doi:10.2466/pms.1989.68.3c.1191

20. Chan DW, Lai B. Psychological aspects of long-distance running among Chinese male runners in Hong Kong. *Int J Psychosom Off Publ Int Psychosom Inst*. 1990;37(1-4):30-34.

21. Chapman CL, De Castro JM. Running addiction: measurement and associated psychological characteristics. *J Sports Med Phys Fitness*. 1990;30(3):283-290.

22. Guyot WG. Psychological and medical factors associated with pain running. *J Sports Med Phys Fitness*. 1991;31(3):452-460.

23. Maresh CM, Sheckley BG, Allen GJ, Camaione DN, Sinatra ST. Middle age male distance runners: physiological and psychological profiles. *J Sports Med Phys Fitness*. 1991;31(3):461-469.

24. Gleaves DH, Williamson DA, Fuller RD. Bulimia nervosa symptomatology and body image disturbance associated with distance running and weight loss. *Br J Sports Med*. 1992;26(3):157-160. doi:10.1136/bjsm.26.3.157

25. Coen SP, Ogles BM. Psychological characteristics of the obligatory runner: A critical examination of the anorexia analogue hypothesis. *J Sport Exerc Psychol*. 1993;15(3):338-354.

26. Furst DM, Germone K. Negative addiction in male and female runners and exercisers. *Percept Mot Skills*. 1993;77(1):192-194. doi:10.2466/pms.1993.77.1.192

27. Masters KS, Ogles BM, Jolton JA. The development of an instrument to measure motivation for marathon running: the Motivations of Marathoners Scales (MOMS). *Res Q Exerc Sport*. 1993;64(2):134-143. doi:10.1080/02701367.1993.10608790

28. Pierce EF, McGowan RW, Lynn TD. Exercise dependence in relation to competitive orientation of runners. *J Sports Med Phys Fitness*. 1993;33(2):189-193.

29. Klock SC, DeSouza MJ. Eating disorder characteristics and psychiatric symptomatology of eumenorrheic and amenorrheic runners. *Int J Eat Disord*. 1995;17(2):161-166. doi:10.1002/1098-108x(199503)17:2<161::aid-eat2260170209>3.0.co;2-r

30. Thornton EW, Scott SE. Motivation in the committed runner: correlations between self-report scales and behaviour. *Health Promot Int*. 1995;10(3):177-184. doi:10.1093/heapro/10.3.177

31. Powers PS, Schocken DD, Boyd FR. Comparison of habitual runners and anorexia nervosa patients. *Int J Eat Disord*. 1998;23(2):133-143. doi:10.1002/(sici)1098-108x(199803)23:2<133::aid-eat3>3.0.co;2-l

32. Slay HA, Hayaki J, Napolitano MA, Brownell KD. Motivations for running and eating attitudes in obligatory versus nonobligatory runners. *Int J Eat Disord*. 1998;23(3):267-275. doi:10.1002/(sici)1098-108x(199804)23:3<267::aid-eat4>3.0.co;2-h

33. Ryujin DH, Breaux C, Marks AD. Symptoms of eating disorders among female distance runners: can the inconsistencies be unraveled? *Women Health*. 1999;30(1):71-83. doi:10.1300/j013v30n01_05

34. Leedy G. Commitment to Distance Running: Coping Mechanisms or Addiction. *J Sport Behav Mob Ala*. 2000;23(3):255–270.

35. Edwards SD, Ngcobo HS, Edwards DJ, Palavar K. Exploring the relationship between physical activity, psychological well-being and physical self- perception in different exercise groups. *South Afr J Res Sport Phys Educ Recreat*. 2005;27(1):59-74-74.

36. Schnohr P, Kristensen TS, Prescott E, Scharling H. Stress and life dissatisfaction are inversely associated with jogging and other types of physical activity in leisure time--The Copenhagen City Heart Study. *Scand J Med Sci Sports*. 2005;15(2):107-112. doi:10.1111/j.1600-0838.2004.00394.x

37. Strachan SM, Woodgate J, Brawley LR, Tse A. The Relationship of Self-Efficacy and Self-Identity to Long-Term Maintenance of Vigorous Physical Activity. *J Appl Biobehav Res*. 2005;10(2):98-112. doi:10.1111/j.1751-9861.2005.tb00006.x

38. Galper DI, Trivedi MH, Barlow CE, Dunn AL, Kampert JB. Inverse association between physical inactivity and mental health in men and women. *Med Sci Sports Exerc*. 2006;38(1):173-178. doi:10.1249/01.mss.0000180883.32116.28

39. Luszczynska A, Mazurkiewicz M, Ziegelmann JP, Schwarzer R. Recovery self-efficacy and intention as predictors of running or jogging behavior: A cross-lagged panel analysis over a two-year period. *Psychol Sport Exerc*. 2007;8(2):247-260. doi:10.1016/j.psychsport.2006.03.010

40. Smith D, Wright C, Winrow D. Exercise dependence and social physique anxiety in competitive and non-competitive runners. *Int J Sport Exerc Psychol*. 2010;8(1):61-69. doi:10.1080/1612197X.2010.9671934

41. Gapin JI, Petruzzello SJ. Athletic identity and disordered eating in obligatory and non-obligatory runners. *J Sports Sci*. 2011;29(10):1001-1010. doi:10.1080/02640414.2011.571275

42. Wadas G, DeBeliso M. Disordered eating, eating attitudes, and reasons for exercise among male high school cross country runners. *Sport J*. 2014;17. Accessed July 15, 2020. https://www.cabdirect.org/cabdirect/abstract/20153072464

43. Download citation of The relationship between motivations, perceived control, and mental toughness among marathon runners. ResearchGate. Accessed July 15, 2020. https://www.researchgate.net/publication/325793259_The_relationship_between_motivations_perceived_control_and_mental_toughness_among_marathon_runners

44. Lucidi F, Pica G, Mallia L, et al. Running away from stress: How regulatory modes prospectively affect athletes' stress through passion. *Scand J Med Sci Sports*. 2016;26(6):703-711. doi:10.1111/sms.12496

45. Batmyagmar D, Kundi M, Ponocny-Seliger E, et al. High intensity endurance training is associated with better quality of life, but not with improved cognitive functions in elderly marathon runners. *Sci Rep*. 2019;9(1):4629. doi:10.1038/s41598-019-41010-w

46. Cleland V, Nash M, Sharman MJ, Claflin S. Exploring the Health-Promoting Potential of the "parkrun" Phenomenon: What Factors are Associated With Higher Levels of Participation? *Am J Health Promot AJHP*. 2019;33(1):13-23. doi:10.1177/0890117118770106

47. Lukács A, Sasvári P, Varga B, Mayer K. Exercise addiction and its related factors in amateur runners. *J Behav Addict*. 2019;8(2):343-349. doi:10.1556/2006.8.2019.28

48. Nowlis DP, Greenberg N. Empirical description of effects of exercise on mood. *Percept Mot Skills*. 1979;49(3):1001-1002. doi:10.2466/pms.1979.49.3.1001

49. Wilson VE, Berger BG, Bird EI. Effects of running and of an exercise class on anxiety. *Percept Mot Skills*. 1981;53(2):472-474. doi:10.2466/pms.1981.53.2.472

50. Markoff RA, Ryan P, Young T. Endorphins and mood changes in long-distance running. *Med Sci Sports Exerc*. 1982;14(1):11-15. doi:10.1249/00005768-198201000-00002

51. Thaxton L. Physiological and Psychological Effects of Short-term Exercise Addiction on Habitual Runners. *J Sport Exerc Psychol*. 1982;4(1):73-80. doi:10.1123/jsp.4.1.73

52. McGowan RW, Pierce EF, Jordan D. Mood alterations with a single bout of physical activity. *Percept Mot Skills*. 1991;72(3 Pt 2):1203-1209. doi:10.2466/pms.1991.72.3c.1203

53. Goode KT, Roth DL. Factor Analysis of Cognitions during Running: Association with Mood Change. *J Sport Exerc Psychol*. 1993;15(4):375-389. doi:10.1123/jsep.15.4.375

54. Morris M, Salmon P. Qualitative and quantitative effects of running on mood. *J Sports Med Phys Fitness*. 1994;34(3):284-291.

55. Rudolph DL, Butki BD. Self-efficacy and affective responses to short bouts of exercise. *J Appl Sport Psychol*. 1998;10(2):268-280. doi:10.1080/10413209808406393

56. Cox RH, Thomas TR, Davis JE. Positive and negative affect associated with an acute bout of aerobic exercise. *J Exerc Physiol Online*. 2001;4:13-20.

57. O'Halloran PD, Murphy GC, Webster KE. Measure of beliefs about improvements in mood associated with exercise. *Psychol Rep*. 2002;90(3 Pt 1):834-840. doi:10.2466/pr0.2002.90.3.834

58. Szabo A. The Acute Effects of Humor and Exercise on Mood and Anxiety. *J Leis Res*. 2003;35(2):152-162. doi:10.1080/00222216.2003.11949988

59. O'Halloran PD, Murphy GC, Webster K. Mood during a 60-minute treadmill run: Timing and type of mood change. *Int J Sport Psychol*. 2004;35:309-327.

60. Robbins LB, Pender NJ, Ronis DL, Kazanis AS, Pis MB. Physical activity, self-efficacy, and perceived exertion among adolescents. *Res Nurs Health*. 2004;27(6):435-446. doi:10.1002/nur.20042

61. Pretty J, Peacock J, Sellens M, Griffin M. The mental and physical health outcomes of green exercise. *Int J Environ Health Res*. 2005;15(5):319-337. doi:10.1080/09603120500155963

62. Hoffman MD, Hoffman DR. Exercisers achieve greater acute exercise-induced mood enhancement than nonexercisers. *Arch Phys Med Rehabil*. 2008;89(2):358-363. doi:10.1016/j.apmr.2007.09.026

63. Kwan BM, Bryan AD. Affective response to exercise as a component of exercise motivation: Attitudes, norms, self-efficacy, and temporal stability of intentions. *Psychol Sport Exerc*. 2010;11(1):71-79. doi:10.1016/j.psychsport.2009.05.010

64. Weinstein AA, Deuster PA, Francis JL, Beadling C, Kop WJ. The Role of Depression in Short-Term Mood and Fatigue Responses to Acute Exercise. *Int J Behav Med*. 2010;17(1):51-57. doi:10.1007/s12529-009-9046-4

65. Anderson RJ, Brice S. The mood-enhancing benefits of exercise: Memory biases augment the effect. *Psychol Sport Exerc*. 2011;12(2):79-82. doi:10.1016/j.psychsport.2010.08.003

66. Kane I, Robertson R, Fertman C, Nagle E, McConnaha W, Rabin B. Self-efficacy and enjoyment of middle school children performing the Progressive Aerobic Cardiovascular Endurance Run (PACER). *Percept Mot Skills*. 2013;117:470-483. doi:10.2466/29.25.PMS.117x23z3

67. Szabo A, Abrahám J. The psychological benefits of recreational running: a field study. *Psychol Health Med*. 2013;18(3):251-261. doi:10.1080/13548506.2012.701755

68. McDowell CP, Campbell MJ, Herring MP. Sex-Related Differences in Mood Responses to Acute Aerobic Exercise. *Med Sci Sports Exerc*. 2016;48(9):1798-1802. doi:10.1249/MSS.0000000000000969

69. Rogerson M, Brown DK, Sandercock G, Wooller J-J, Barton J. A comparison of four typical green exercise environments and prediction of psychological health outcomes. *Perspect Public Health*. 2016;136(3):171-180. doi:10.1177/1757913915589845

70. Edwards MK, Rhodes RE, Loprinzi PD. A Randomized Control Intervention Investigating the Effects of Acute Exercise on Emotional Regulation. *Am J Health Behav*. 2017;41(5):534-543. doi:10.5993/AJHB.41.5.2

71. Krotee ML. The Effects of Various Physical Activity Situational Settings on the Anxiety Level of Children. *J Sport Behav Mob Ala*. 1980;3(4):158–164.

72. Wildmann J, Krüger A, Schmole M, Niemann J, Matthaei H. Increase of circulating beta-endorphin-like immunoreactivity correlates with the change in feeling of pleasantness after running. *Life Sci*. 1986;38(11):997-1003. doi:10.1016/0024-3205(86)90233-x

73. O'Connor PJ, Carda RD, Graf BK. Anxiety and intense running exercise in the presence and absence of interpersonal competition. *Int J Sports Med*. 1991;12(4):423-426. doi:10.1055/s-2007-1024706

74. Nabetani T, Tokunaga M. The effect of short-term (10- and 15-min) running at self-selected intensity on mood alteration. *J Physiol Anthropol Appl Human Sci*. 2001;20(4):231-239. doi:10.2114/jpa.20.233

75. Bodin M, Hartig T. Does the outdoor environment matter for psychological restoration gained through running? *Psychol Sport Exerc*. 2003;4(2):141-153. doi:10.1016/S1469-0292(01)00038-3

76. Butryn TM, Furst DM. The effects of park and urban settings on the moods and cognitive strategies of female runners. *J Sport Behav*. 2003;26(4):335-355.

77. Kerr JH, Fujiyama H, Sugano A, Okamura T, Chang M, Onouha F. Psychological responses to exercising in laboratory and natural environments. *Psychol Sport Exerc*. 2006;7(4):345-359. doi:10.1016/j.psychsport.2005.09.002

78. Rose EA, Parfitt G. Exercise experience influences affective and motivational outcomes of prescribed and self-selected intensity exercise. *Scand J Med Sci Sports*. 2012;22(2):265-277. doi:10.1111/j.1600-0838.2010.01161.x

79. Reed K, Wood C, Barton J, Pretty JN, Cohen D, Sandercock GRH. A repeated measures experiment of green exercise to improve self-esteem in UK school children. *PloS One*. 2013;8(7):e69176. doi:10.1371/journal.pone.0069176

80. Harte JL, Eifert GH. The effects of running, environment, and attentional focus on athletes' catecholamine and cortisol levels and mood. *Psychophysiology*. 1995;32(1):49-54. doi:10.1111/j.1469-8986.1995.tb03405.x

81. Berger B, Owen D, Motl R, Parks L. Relationship between expectancy of psychological benefits and mood alteration in joggers. *Int J Sport Psychol*. 1998;29:1-16.

82. Markowitz SM, Arent SM. The exercise and affect relationship: evidence for the dual-mode model and a modified opponent process theory. *J Sport Exerc Psychol*. 2010;32(5):711-730. doi:10.1123/jsep.32.5.711

83. Lion LS. Psychological effects of jogging: a preliminary study. *Percept Mot Skills*. 1978;47(3 Pt 2):1215-1218. doi:10.2466/pms.1978.47.3f.1215

84. Blue FR. Aerobic running as a treatment for moderate depression. *Percept Mot Skills*. 1979;48(1):228. doi:10.2466/pms.1979.48.1.228

85. Young RJ. The effect of regular exercise on cognitive functioning and personality. *Br J Sports Med*. 1979;13(3):110-117. doi:10.1136/bjsm.13.3.110

86. Blumenthal JA, Williams RS, Needels TL, Wallace AG. Psychological changes accompany aerobic exercise in healthy middle-aged adults. *Psychosom Med*. 1982;44(6):529-536. doi:10.1097/00006842-198212000-00004

87. Trujillo CM. The effect of weight training and running exercise intervention programs on the self-esteem of college women. *Int J Sport Psychol*. 1983;14(3):162-173.

88. Tuckman BW, Hinkle JS. An experimental study of the physical and psychological effects of aerobic exercise on schoolchildren. *Health Psychol Off J Div Health Psychol Am Psychol Assoc*. 1986;5(3):197-207. doi:10.1037//0278-6133.5.3.197

89. Doyne EJ, Ossip-Klein DJ, Bowman ED, Osborn KM, McDougall-Wilson IB, Neimeyer RA. Running versus weight lifting in the treatment of depression. *J Consult Clin Psychol*. 1987;55(5):748-754. doi:10.1037//0022-006x.55.5.748

90. Fremont J, Craighead LW. Aerobic exercise and cognitive therapy in the treatment of dysphoric moods. *Cogn Ther Res*. 1987;11(2):241-251. doi:10.1007/BF01183268

91. Hannaford CP, Harrell EH, Cox K. Psychophysiological Effects of A Running Program on Depression and Anxiety in A Psychiatric Population. *Psychol Rec*. 1988;38(1):37-48. doi:10.1007/BF03395005

92. Long BC, Haney CJ. Long-Term Follow-up of Stressed Working Women: A Comparison of Aerobic Exercise and Progressive Relaxation. *J Sport Exerc Psychol*. 1988;10(4):461-470. doi:10.1123/jsep.10.4.461

93. Simons CW, Birkimer JC. An exploration of factors predicting the effects of aerobic conditioning on mood state. *J Psychosom Res*. 1988;32(1):63-75. doi:10.1016/0022-3999(88)90089-X

94. Moses J, Steptoe A, Mathews A, Edwards S. The effects of exercise training on mental well-being in the normal population: a controlled trial. *J Psychosom Res*. 1989;33(1):47-61. doi:10.1016/0022-3999(89)90105-0

95. Ossip-Klein DJ, Doyne EJ, Bowman ED, Osborn KM, McDougall-Wilson IB, Neimeyer RA. Effects of running or weight lifting on self-concept in clinically depressed women. *J Consult Clin Psychol*. 1989;57(1):158-161. doi:10.1037//0022-006x.57.1.158

96. Morris M, Steinberg H, Sykes EA, Salmon P. Effects of temporary withdrawal from regular running. *J Psychosom Res*. 1990;34(5):493-500. doi:10.1016/0022-3999(90)90023-w

97. Friedman E, Berger BG. Influence of gender, masculinity, and femininity on the effectiveness of three stress reduction techniques: Jogging, relaxation response, and group interaction. *J Appl Sport Psychol*. 1991;3(1):61-86. doi:10.1080/10413209108406435

98. Williams TJ, Krahenbuhl GS, Morgan DW. Mood state and running economy in moderately trained male runners. *Med Sci Sports Exerc*. 1991;23(6):727-731.

99. Kerr JH, Vlaswinkel EH. Self-reported mood and running under natural conditions. *Work Stress*. 1993;7(2):161-177. doi:10.1080/02678379308257058

100. Long BC. Aerobic conditioning (jogging) and stress inoculation interventions: an exploratory study of coping. *Int J Sport Psychol*. 1993;24(2):94-109.

101. Berger BG, Friedman E. Comparison of Jogging, the Relaxation Response, and Group Interaction for Stress Reduction. *J Sport Exerc Psychol*. 1988;10(4):431-447. doi:10.1123/jsep.10.4.431

102. Berger BG, Owen DR. Relation of low and moderate intensity exercise with acute mood change in college joggers. *Percept Mot Skills*. 1998;87(2):611-621. doi:10.2466/pms.1998.87.2.611

103. Szabo A, Frenkl R, Janek G, Kálmán L, Lászay D. Runners' anxiety and mood on running and non-running days: An in situ daily monitoring study. *Psychol Health Med*. 1998;3(2):193-199. doi:10.1080/13548509808402235

104. Broman-Fulks JJ, Berman ME, Rabian BA, Webster MJ. Effects of aerobic exercise on anxiety sensitivity. *Behav Res Ther*. 2004;42(2):125-136. doi:10.1016/S0005-7967(03)00103-7

105. Judith Haffmans PM, Kleinsman ACM, van Weelden C, Huijbrechts IPAM, Hoencamp E. Comparing running therapy with physiotraining therapy in the treatment of mood disorders. *Acta Neuropsychiatr*. 2006;18(3-4):173-176. doi:10.1111/j.1601-5215.2006.00115.x

106. Thornton E, Cronholm P, McCray L, Webner D. Does Marathon Training Adversely Affect Baseline Anxiety Levels? *AMAA J*. 2008;21(3):5-9.

107. Scholz U, Nagy G, Schüz B, Ziegelmann JP. The role of motivational and volitional factors for self-regulated running training: associations on the between- and within- person level. *Br J Soc Psychol*. 2008;47(Pt 3):421-439. doi:10.1348/014466607X266606

108. Kalak N, Gerber M, Kirov R, et al. Daily morning running for 3 weeks improved sleep and psychological functioning in healthy adolescents compared with controls. *J Adolesc Health Off Publ Soc Adolesc Med*. 2012;51(6):615-622. doi:10.1016/j.jadohealth.2012.02.020

109. Inoue Y, Funk D, Jordan JS. The role of running involvement in creating self-sufficiency for homeless individuals through a community-based running program. *J Sport Manag*. 2013;27(6):439-452. doi:10.1123/jsm.27.6.439

110. Samson A, Solmon M, Stewart L. Changes in self-efficacy and affect during a 15-week marathon training program. *Int J Sport Psychol*. 2013;44(1):55-68.

111. Doose M, Ziegenbein M, Hoos O, et al. Self-selected intensity exercise in the treatment of major depression: A pragmatic RCT. *Int J Psychiatry Clin Pract*. 2015;19(4):266-275. doi:10.3109/13651501.2015.1082599

112. von Haaren B, Haertel S, Stumpp J, Hey S, Ebner-Priemer U. Reduced emotional stress reactivity to a real-life academic examination stressor in students participating in a 20-week aerobic exercise training: A randomised controlled trial using Ambulatory Assessment. *Psychol Sport Exerc*. 2015;20:67-75. doi:10.1016/j.psychsport.2015.04.004

113. Kahan D, McKenzie TL. Physical Activity and Psychological Correlates During an After-School Running Club. *Am J Health Educ*. 2018;49(2):113-123. doi:10.1080/19325037.2017.1414646

114. Keating LE, Becker S, McCabe K, et al. Effects of a 12-week running programme in youth and adults with complex mood disorders. *BMJ Open Sport Exerc Med*. 2018;4(1). doi:10.1136/bmjsem-2017-000314

115. Nezlek JB, Cypryańska M, Cypryański P, et al. Within-Person Relationships Between Recreational Running and Psychological Well-Being. *J Sport Exerc Psychol*. 2018;40(3):146-152. doi:10.1123/jsep.2017-0244

116. Kruisdijk F, Hopman-Rock M, Beekman ATF, Hendriksen I. EFFORT-D: results of a randomised controlled trial testing the EFFect of running therapy on depression. *BMC Psychiatry*. 2019;19(1):170. doi:10.1186/s12888-019-2156-x
